# Genome-Guided Insights into the Plant Growth Promotion Capabilities of the Physiologically Versatile *Bacillus aryabhattai* Strain AB211

**DOI:** 10.3389/fmicb.2017.00411

**Published:** 2017-03-21

**Authors:** Chandrima Bhattacharyya, Utpal Bakshi, Ivy Mallick, Shayantan Mukherji, Biswajit Bera, Abhrajyoti Ghosh

**Affiliations:** ^1^Department of Biochemistry, Bose InstituteKolkata, India; ^2^Structural Biology and Bioinformatics Division, CSIR – Indian Institute of Chemical BiologyKolkata, India; ^3^Tea Board of India, Ministry of Commerce and IndustryKolkata, India

**Keywords:** *Bacillus aryabhattai*, next generation sequencing, bacterial genomics, comparative genomics, pangenome, plant growth-promoting rhizobacteria, root colonization

## Abstract

*Bacillus aryabhattai* AB211 is a plant growth promoting, Gram-positive firmicute, isolated from the rhizosphere of tea (*Camellia sinensis*), one of the oldest perennial crops and a major non-alcoholic beverage widely consumed all over the world. The whole genome of *B. aryabhattai* AB211 was sequenced, annotated and evaluated with special focus on genomic elements related to plant microbe interaction. It’s genome sequence reveals the presence of a 5,403,026 bp chromosome. A total of 5226 putative protein-coding sequences, 16 rRNA, 120 tRNA, 8 ncRNAs, 58 non-protein coding genes, and 11 prophage regions were identified. Genome sequence comparisons between strain AB211 and other related environmental strains of *B. aryabhattai*, identified about 3558 genes conserved among all *B. aryabhattai* genomes analyzed. Most of the common genes involved in plant growth promotion activities were found to be present within core genes of all the genomes used for comparison, illustrating possible common plant growth promoting traits shared among all the strains of *B. aryabhattai*. Besides the core genes, some genes were exclusively identified in the genome of strain AB211. Functional annotation of the genes predicted in the strain AB211 revealed the presence of genes responsible for mineral phosphate solubilization, siderophores, acetoin, butanediol, exopolysaccharides, flagella biosynthesis, surface attachment/biofilm formation, and indole acetic acid production, most of which were experimentally verified in the present study. Genome analysis and experimental evidence suggested that AB211 has robust central carbohydrate metabolism implying that this bacterium can efficiently utilize the root exudates and other organic materials as an energy source. Genes for the production of peroxidases, catalases, and superoxide dismutases, that confer resistance to oxidative stresses in plants were identified in AB211 genome. Besides these, genes for heat shock tolerance, cold shock tolerance, glycine-betaine production, and antibiotic/heavy metal resistance that enable bacteria to survive biotic/abiotic stress were also identified. Based on the genome sequence information and experimental evidence as presented in this study, strain AB211 appears to be metabolically diverse and exhibits tremendous potential as a plant growth promoting bacterium.

## Introduction

Plant growth promoting rhizobacteria (PGPR) are a heterogeneous group of bacteria that are present in the rhizosphere and exert beneficial effects on plant development (Kloepper et al., 1980; Lugtenberg and Kamilova, 2009). The rhizosphere is an extremely dynamic micro-niche in which complex interactions occur between plant roots and microorganisms (Molina et al., 2000; Uroz et al., 2010; Blom et al., 2011). Microorganisms compete for colonization on the plant roots and simultaneously function as phytostimulators, biofertilizers, and as antagonists (biopesticides) (Khalid et al., 2004; Vassilev et al., 2006; Spaepen et al., 2007; Van Wees et al., 2008; Lugtenberg and Kamilova, 2009; Matilla et al., 2010; Blom et al., 2011). A variety of rhizobacteria, including *Pseudomonas* and *Bacillus* spp. are commonly found in the rhizosphere of a wide variety of plant species and stimulate plant growth through direct or indirect mechanisms (Podile and Kishore, 2006). Direct plant growth promotion is often executed via increasing bioavailability of mineral nutrients such as nitrogen, phosphorous, and iron (Lugtenberg and Kamilova, 2009) or by providing amino acids and other nutritional factors (Simons et al., 1997; Compant et al., 2010; Vial et al., 2011) or by synthesis of plant growth regulating compounds such as indole acetic acid (IAA), gibberellins, acetoin (3-hydroxy-2-butanone), 2,3-butanediol, and cytokinin (Arshad and Frankenberger, 1991; Spaepen et al., 2007; Kang et al., 2014; Kudoyarova et al., 2014; Piechulla and Degenhardt, 2014). Besides, rhizobacteria often metabolize compounds like phenylacetic acid (PAA), the stress ethylene precursor 1-aminocyclopropane-1-carboxylic acid (ACC), and show chemotaxis toward the source of gamma-aminobutyrate (GABA) and together they contribute toward successful plant–microbe interaction (Glick et al., 1998; Shah et al., 1998; Glick, 2004; Onofre-Lemus et al., 2009; Reyes-Darias et al., 2015). There exist several indirect mechanisms as well, through which many rhizobacteria promote plant growth. Such mechanisms involve events like: synthesis of antibiotics, antifungals, and biopesticides (Hammer et al., 1997; Lugtenberg and Kamilova, 2009; Perez et al., 2011; Ahemad and Khan, 2012), production of biocides such as hydrogen cyanide and fungal cell wall degrading enzymes, e.g., chitinase and β-1,3-glucanase (Zhang and Yuen, 2000; Haas and Keel, 2003; Malfanova et al., 2011), and production of iron chelating small molecules, siderophores to compete for iron in the rhizospheric environment to achieve better selection (Lemanceau et al., 2009; Schalk et al., 2011). Among rhizobacteria, *Pseudomonas* spp. are the most widely studied in relation to plant growth promotion activities (Meyer and Linderman, 1986; Paulsen et al., 2005; Goswami et al., 2015; Godino et al., 2016). Several plant growth promoting *Pseudomonas* spp. have contributed significantly to understand the mechanisms that are involved in phytostimulation and disease suppression. However, studies have shown that biological preparations from spore-forming *Bacillus* spp. are preferred due to their persistent viability that supports commercialization (Haas and Defago, 2005). Compared to plant growth promoting *Pseudomonas*, relatively little is known about the growth promotion features of plant associated *Bacillus* spp. Despite their well established effect on plant growth promotion and their role as biocontrol agents, *Bacillus* spp. have been considered as typical soil bacteria for a long time (Kloepper et al., 2004; Compant et al., 2005). In recent years, researchers have demonstrated the great potential of various *Bacillus* isolates in plant growth promotion, in biocontrol as well as in systemic acquired resistance in plants (Chen et al., 2007; Wang et al., 2009).

The *Bacillus aryabhattai* was first isolated, and identified from cryotubes used to collect air samples at an altitude of 27 to 41 km in 2009 (Shivaji et al., 2009). Since then, some *B. aryabhattai* strains have been isolated from various environments like sugar cane and rice plantation soil (Tanamool et al., 2013; Pailan et al., 2015), rhizosphere of horseweed (common wild plant) (Lee et al., 2012) and *Spartina maritima* (Mesa et al., 2015), dense forest soil (Chanasit et al., 2014), an urban tunnel (Park et al., 2012), and from deep sea water (Wen et al., 2015). Preliminary plant growth promotion capacity of *B. aryabhattai* isolates (LS9, LS11, LS12, and LS15) was demonstrated earlier using *Xanthium italicum* as the model plant system (Lee et al., 2012). However, little is known about the genomic potential of these isolates on phytostimulation and biocontrol. The present study aims at the thorough elucidation of the plant growth promoting traits and to identify other metabolic features of *B. aryabhattai* AB211, rhizobacteria isolated from tea rhizosphere. To investigate the genomic potential, and to explore the habitat-specific variations in the gene repertoire of *B. aryabhattai* AB211, we performed genome sequencing and comparative genomics of the strain AB211 and other related environmental strains of *B. aryabhattai*. An exploration of the genome sequence has identified key attributes essential for possible colonization, establishment, and interaction of the strain AB211 with the host plant. We also performed a detailed biochemical/metabolic characterization, biofilm/root association, and plant growth promotion studies to consolidate on the genomic insights.

## Materials and Methods

### Strain and Culture Media

*Bacillus aryabhattai* AB211 was isolated using a functional screening based method described previously (Ghosh et al., 2007), from tea rhizosphere of Rohini, Darjeeling district, West Bengal, India. Strain AB211 was routinely grown in M9 minimal medium supplemented with glucose. The culture was incubated at 37°C on a rotary shaker (150 rpm) for desired period with or without amendments of antibiotic (tetracycline 35 μg ml^-1^).

To test the ability of *B. aryabhattai* AB211 to use inorganic and organic insoluble phosphate as a phosphorous source, Pikovskaya agar plates were used (Pikovskaya, 1948). The reaction was considered positive when a clear halo surrounding the bacterial colonies was observed after 3–7 days of incubation at 37°C. Furthermore, the ability of the strain AB211 to solubilize inorganic phosphate was quantitatively assessed. 50 μl of overnight grown culture was inoculated in 5 ml Pikovskaya’s broth and incubated for 7 days. Uninoculated medium was used as a control. Following incubation, the cell suspension was centrifuged, and the available phosphate content of the supernatant was estimated by malachite green method (Ghosh et al., 2011). The experiment was performed in triplicate.

Chrome azurol sulfonate (CAS) agar solid medium was used to screen siderophore production (Alexander and Zuberer, 1991). The reaction was considered positive when an orange halo surrounding the bacterial colony appeared due to the removal of iron from CAS by the siderophore. Quantification of siderophore production was estimated by following the formula: % siderophore unit = [(Ar-As)/Ar] × 100, where, Ar = absorbance of reference (minimal medium + CAS assay solution), As = absorbance of the sample.

To detect the production of ammonia, the strain was grown in 4% peptone broth and incubated for 7 days at 37°C. Following incubation, 0.5 mL of Nessler’s reagent was added to the bacterial suspension. The development of deep yellow color with brown precipitation indicated ammonia production.

Production of indole-3-acetic acid (IAA) by the strain AB211, was estimated by growing the isolate in M9 medium, supplemented with L-tryptophan as a precursor of IAA at different concentrations (0, 50, 100, 200, 300, 400, and 500 mg l^-1^), and incubated for 48 h. The supernatant of the culture fluid was mixed with Salkowski’s coloring reagent (50 ml of 35% HClO_4_, 1 ml of 0.5 M FeCl_3_) in the ratio of 2:1 and incubated in the dark for 30 min. The absorbance was measured at 540 nm.

The synthesis of exopolysaccharides (EPS) was estimated following the method described previously (Chiba et al., 2015). Quantification of proteins in the isolated EPS fractions was carried out using standard Bradford protein assay and total saccharide was estimated following phenol-sulfuric acid method.

### Identification and Characterization of *B. aryabhattai* AB211

Bacterial identification was conducted based on morphology and biochemical characterization. The morphological, cultural, and physiological characteristics of the isolated strain was compared with data from Bergey’s Manual of Determinative Bacteriology (Holtz, 1993). To identify the strain AB211 by carbon source utilization pattern, the GEN III MicroPlate^TM^ test (Biolog Inc., Hayward, CA, USA) was carried out, which provides a standardized micro-method using 94 biochemical tests to profile, and characterize a broad range of bacteria (Bochner, 1989). This technique analyzes a microorganism depending on phenotypic tests which include 71 carbon source utilization assays and 23 chemical sensitivity assays.

### Genomic DNA Isolation and Genome Sequencing

Total DNA was isolated from *B. aryabhattai* AB211 as described for *B. subtilis* according to the method of Bron and Venema (1972). Genome sequencing of *B. aryabhattai* AB211 was performed at Bionivid Technology Pvt. Ltd (Bengaluru, Karnataka, India) using an Illumina platform using HiSeq Illumina paired-end technology with 151 bp of reads. All reads were quality assessed using NGSQC toolkit. Primary genome assembly using Velvet (v 1.2.10) (Zerbino and Birney, 2008), and further scaffolding of primary assembly using SSPASE (v 3.0) (Boetzer et al., 2011) were performed. *De novo* genome validation and quality control were performed using Bowtie 2 (v 2.2.2) (Langmead et al., 2009).

### Genome Analysis and Annotation

Putative coding sequences (CDS) were identified by the RAST server (Aziz et al., 2008; Overbeek et al., 2014). All CDS identified were manually reviewed, and false CDS were flagged as “artifact.” The remaining CDS were then submitted to automatic functional annotation via BLAST searches against the UniProt databank to determine significant homology. Putative tRNAs were identified using ARAGORN (v 1.2.36) (Laslett and Canback, 2004), and rRNAs were identified using RNAmmer 1.2 server (Lagesen et al., 2007). The presence of plasmid derived sequence was verified using Webcutter (v 2.0), and Plasmid Finder (v1.3) (Carattoli et al., 2014). Detection of bacteriophage sequences was performed using PHAST (Zhou et al., 2011). The taxonomic identification was performed using MEGA6 (Tamura et al., 2013). Finally, genome finishing was carried out using CONTIGuator (V 2.7) (Galardini et al., 2011) applying closest homolog of the assembled genome as a reference.

### Genome Comparisons

For comparative genomic analysis of AB211 strain, genome sequences of seven other *B. aryabhattai* strains (**Table 1**) were downloaded from NCBI ^1^. For identification of reference genome, a whole genome nucleotide based alignment (BLAST) with strain AB211 in the NCBI ‘nr’ database was performed with >95% identity and coverage values. Identified highly similar genomes (**Table 1**) were then subjected to ANI analysis, and the most identical complete genome sequence, viz. *B. megaterium* Q3, also having close evolutionary relationship with strain AB211 as evident in phylogenetic analysis, was selected as reference genome for genome alignment of AB211. To arrange the genomic assemblies of the draft genome sequences, contigs were ordered and oriented by promer (Kurtz et al., 2004) based on their genomic alignments with the complete genome sequence of *B. megaterium* Q3. Ordered contigs were then pasted together to form a pseudo-chromosome, where contig boundaries were separated by a spacer sequence, as suggested previously (Tettelin et al., 2005).

**Table 1:** General features and characteristics of eight *Bacillus aryabhattai* genomes used for comparative genomics analysis.

Strain	BioSample	BioProject	Assembly	Level	Size (Mb)	GC%	WGS	Scaffolds	Gene	Protein
*B. aryabhattai* AB211	SAMN05425717	PRJNA224116	GCF_001858395.1	Scaffold	5.4	37.8	MCAN01	23	5468	5226
*B. aryabhattai* T61	SAMN02756826	PRJNA246347	GCA_001025015.1	Scaffold	5.3	38	JMTL01	15	5335	5142
*B. aryabhattai* GZ03	SAMN02909700	PRJNA255059	GCA_000785045.1	Contig	5.1	38.1	JPIE01	37	5114	5021
*B. aryabhattai* C765	SAMN03291005	PRJNA273363	GCA_000876465.1	Contig	5.5	37.7	JXRC01	49	5597	5410
*B. aryabhattai* B8W22	SAMN03159255	PRJNA211969	GCA_000956595.1	Contig	5.1	38	JYOO01	72	5077	4846
*B. aryabhattai* LK25	SAMN03751785	PRJNA277418	GCA_001038965.1	Contig	5.4	37.8	LDWN01	48	5487	5351
*B. aryabhattai* LK20	SAMN03751011	PRJNA285382	GCA_001043825.1	Contig	5.5	37.8	LDUV01	109	5536	5404
*B. aryabhattai* M46	SAMN04386399	PRJNA308054	GCA_001619595.1	Contig	5.0	38.2	LQQQ01	37	4992	4867
*B. megaterium* Q3^#^	SAMN03799770	PRJNA236009	GCA_001050455.1	Complete	5.2	38.2	–	–	5273	5056
*B. megaterium* QM B1551^#^	SAMN02603354	PRJNA30165	GCA_000025825.1	Complete	5.5	37.9	–	–	5548	5286
*B. megaterium* NBRC 15308 ATCC 14581^#^	SAMN03174779	PRJNA238207	GCA_000832985.1	Complete	5.7	37.8	–	–	5790	5532
*B. megaterium* DSM319^#^	SAMN02603894	PRJNA42425	GCA_000025805.1	Complete	5.0	38.1	–	–	5121	4858
*B. megaterium* WSH-002^#^	SAMN02603461	PRJNA71447	GCA_000225265.1	Complete	5.0	38.1	–	–	5114	4776

***^#^**For core and strain-specific gene extrapolation analysis part, these five *B. megaterium* strains, having high evolutionary and genomic similarities with *B. aryabhattai* strains, are also included in our analysis.*

### Core and Strain-Specific Gene Extrapolation

To identify the core and strain-specific genes of AB211, a pan-genome analysis of gene distribution of AB211 was carried out against other *B. aryabhattai* strains. In this study, five strains of *B. megaterium* (**Table 1**), having high genomic and evolutionary similarities with *B. aryabhattai* AB211, were also considered. The pan-genome analysis of these 13 genomes was carried out in BPGA pipeline (Chaudhari et al., 2016), with default criteria. The specific set of genes (present and/or absent) of AB211 was then subjected to Clusters of Orthologous Group (COG) category-wise classification scheme by performing PSI-BLAST against NCBI COG database in WebMGA analysis server with default parameters (Wu et al., 2011). The statistical significance of the abundance of individual COG categories was determined by a 2 × 2 contingency table in STATISTICA version 6 (StatSoft Inc., 2001).

### Identification of Recombination Specific Genes in *B. aryabhattai* AB211

To understand the genome-wise distribution of orthologous clusters and to find out whether recombination has played any role in the evolution of *B. aryabhattai* strains, a genome-wide synteny analysis was performed in GSV (Revanna et al., 2011) with various criteria. Multiple genome alignment utility in progressive Mauve (Darling et al., 2010) was also used for the same. The probable recombination regions were identified from these syntenic blocks. Individual gene sequences were extracted from these homologous clusters and further aligned with ClustalW in MEGA6 (Tamura et al., 2013) to search for any recombination signature present in them by RDP analysis (Martin et al., 2010).

### Phylogenetic Analysis

Based on the obtained 16S rRNA gene sequence of *B. aryabhattai* AB211, and multiple alignments, a phylogenetic tree was constructed by the Neighbor-Joining method, and confidence level was estimated for 1,000 replicates using MEGA6 (Molecular Evolutionary Genetics Analysis) (Tamura et al., 2013). Unaligned regions and gaps were excluded from the analyses. Sequences of representative *Bacillus* strains and out-group were obtained from NCBI GenBank^2^. Core and pan-genome phylogenetic studies were performed in BPGA pipeline with default criteria (Chaudhari et al., 2016).

### Biofilm Assay

The assay was performed in 96-well tissue culture plates (Nunclon Delta Surface, Thermo Scientific) to study the efficiency of the strain AB211 in biofilm formation under varied incubation time in M9 medium (Koerdt et al., 2011). After inoculation with the culture and incubation for 48, 72, and 96 h, respectively, the microtiter plates were cooled down to room temperature and the OD_600_ of the planktonic cells from each well was measured using a plate reader (FLUOstar, OPTIMA) at a wavelength of 600 nm. Culture supernatant was then removed from each well and the cells attached to the well were quantitatively estimated using crystal violet (CV). Ten microliter of a 0.5% solution of CV was added to each well and incubated at room temperature for 10 min. Subsequently, the liquid supernatant was removed from each well and the biofilm cells attached to the well were washed with water. Hundred percentage ethanol was added to release the CV from the biofilm. The absorbance of CV from each well was measured at a wavelength of 570 nm. The percentage of cells within the biofilm was calculated by determining the correlation between the growth of the cells (OD_600 nm_) and the absorbance of CV (OD_570 nm_). Each set was performed in triplicate.

### Scanning Electron Microscopic (SEM) Analysis of *B. aryabhattai* AB211 Planktonic Cells, Biofilm and Its Interaction with Maize Roots

A 16 h grown culture of AB211 was inoculated (1% v/v) in M9 minimal medium and incubated for 24 h at 37°C. After incubation, cells were harvested by centrifugation and washed thrice with sodium phosphate buffer (pH 7.4). Cells were then fixed with 0.5% glutaraldehyde solution and washed thrice with sodium phosphate buffer. Dehydration of cells was carried out in a gradient of ethanol and finally incubated in 100% ethanol for 1 h. Two–three microliter of fixed cells were placed on a small glass slide and dried (Focardi et al., 2010; Banerjee et al., 2011). SEM observation was performed using a scanning electron microscope (Zeiss-EVO18).

For biofilm study, 16 h grown culture of the strain AB211 was inoculated on cover slips (1% v/v) kept in M9 minimal medium and incubated for 48, 72, and 96 h, respectively, at 37°C without disturbance. The biofilm was fixed following the same protocol as mentioned earlier. After complete dehydration, the cover slips were dried and viewed under SEM.

To assess the ability of *B. aryabhattai* AB211 to colonize on maize roots, 50 ml of M9 minimal medium (supplemented with 0.5% w/v glucose), inoculated with the strain AB211 was incubated overnight at 37°C under shaking conditions. The cells were harvested by centrifugation at 6000 rpm for 5 min and were washed twice in M8 buffer (22 mM Na_2_HPO_4_, 22 mM KH_2_PO_4_, 100 mM NaCl, pH 7.0). Finally, the harvested cells were suspended in 250 ml of M8 buffer. Roots of the maize seedlings were washed thoroughly with sterile water to get rid of attached soil particles, and then soaked in bacterial suspension for 1 h under aseptic conditions, and transferred back to sterile bottles. Control was maintained by following the same protocol with M8 buffer, excluding the cells. Both the plants were transferred to plant growth chamber at 28°C with a 16 h light regimen. The roots were then cut and fixed in 2.5% glutaraldehyde in 0.075 M phosphate buffer overnight, and processed further for dehydration and visualization as described above.

### Pot Experiments: Plant Growth Promotion Assay Using Maize Seedlings

Based on the performance in the *in vitro* experiments, *B. aryabhattai* AB211 was further evaluated for its plant growth promoting potential on maize seedlings in pot trials. Surface sterilized maize seeds (variety: Early Golden Bantam) were sown in sterile pots (one seed/pot) filled with sterile soilrite (Keltech Energies Limited, Bangalore, India). After germination, rhizospheres of 7-day-old seedlings were inoculated with the isolate (inoculation with about 10^8^ cfu/ml of AB211 culture; and in soil, resulting cfu of AB211 was approximately 10^7^ cfu/gm). Control was maintained by applying sterile medium without culture on the plants. Sixteen replicates were maintained for both control and treatment sets. The plants were regularly irrigated. Observations were taken on the 15th day of treatment. Growth parameters such as total chlorophyll content, height, wet weight, dry weight of the shoot and root were measured and statistically analyzed.

### Accession Number

The whole-genome shotgun project of *B. aryabhattai* AB211 was deposited at DDBJ/EMBL/GenBank under the accession number MCAN00000000. The accession number for submitted 16S rRNA gene sequence is KP896525.1.

## Results and Discussion

### General Genomic Features

The main features of the *B. aryabhattai* AB211 genome have been summarized in **Table 1**. The circular chromosome (5,403,026 bp) was found to be somewhat smaller to that of the closely related *B. megaterium* (Arya et al., 2014; Wang et al., 2016). The whole genome sequence of strain AB211 was obtained by an Illumina platform using HiSeq Illumina paired-end technology with 151 bp reads. Primary genome assembly using Velvet (v 1.2.10) (Zerbino and Birney, 2008), and further scaffolding of primary assembly using SSPASE (v 3.0) (Boetzer et al., 2011) revealed that the draft genome consists of 23 scaffolds with an average genome length of ∼5.4 Mbp, G+C content of 37.82%, and N_50_ size of 4199117 (∼4.19 Mb) bp. Genome annotation by RAST server revealed that the draft genome has 5226 protein CDSs, 16 rRNA genes, 120 tRNAs, 8 ncRNAs, 58 non-protein coding genes, and 11 prophage regions. No plasmid was identified when analyzed using Webcutter (v 2.0) and Plasmid Finder (v1.3) (Carattoli et al., 2014). The taxonomic identification using MEGA6 (Tamura et al., 2013) revealed that the strain AB211 exhibits closest phylogenetic relationship to the *B. megaterium* strain Q3.

In RAST annotation, genes encoding for transport system, plant–bacterial interaction, secretion system, antibiotic resistance, surface appendages/exopolysaccharides synthesis, heavy metal resistance/mobilization, and stress response were observed. In general, *B. aryabhattai* AB211 genome revealed high metabolic diversity. In reference to plant-microbe interaction, the AB211 genome analysis revealed 300 proteins in carbohydrate metabolism, 54 proteins in flagella assembly, function and signaling; 19 proteins in nitrogen metabolism, 19 proteins in phosphorous metabolism, 29 proteins in siderophore biosynthesis and iron acquisition, 74 proteins in stress response, 43 proteins in antibiotic and heavy metal resistance, 17 proteins in plant hormone/volatile compound synthesis and 16 proteins in aromatic compound degradation pathway (Supplementary Table S1).

### Comparative Genomics of *B. aryabhattai* AB211

**FIGURE 1:**
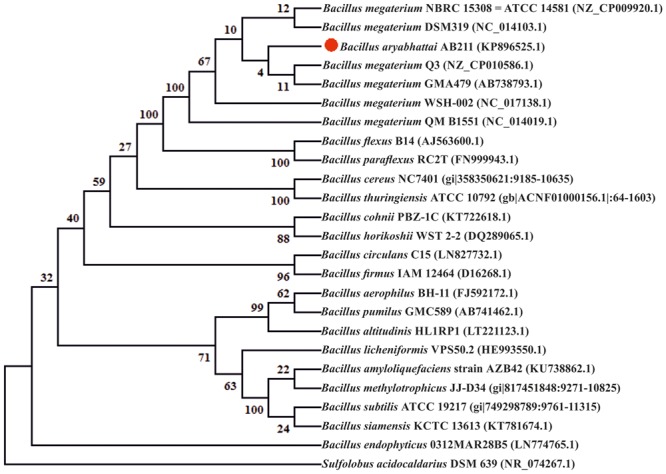
**Phylogenetic tree based on 16S rRNA gene sequences obtained by the neighbor-joining (NJ) method showing the phylogenetic relationship of the *Bacillus aryabhattai* AB211 with the related species.** The bootstrap consensus tree inferred from 1000 replicates is taken to represent the evolutionary history of the taxa analyzed. The percentage of replicate trees in which the associated taxa clustered together in the bootstrap test (1000 replicates) are shown next to the branches. The evolutionary distances were computed using the Jukes–Cantor method and are in the units of the number of base substitutions per site. Evolutionary analyses were conducted in MEGA6. Accession numbers are written after the species or strain name. The tree is rooted with thermoacidophilic crenarchaeon *Sulfolobus acidocaldarius* DSM639 (NR_074267.1).

To understand the evolutionary relationship of *B. aryabhattai* AB211 with other Bacilli, a 16S rRNA Neighbor-Joining phylogeny of AB211 with 24 other known *Bacillus* species was performed. Phylogenetic analysis revealed the close evolutionary relationship of AB211 with *B. megaterium* (**Figure 1**). To further illustrate the evolutionary relationship, both core and pan-genomic phylogeny of *B. aryabhattai* strains were constructed with those strains of *B. megaterium* that have high sequence similarities with *B. aryabhattai* AB211 (>95% identity and coverage) (**Table 1**). Core and pan-matrix phylogeny revealed that, in genome scale, there was very little difference in *B. aryabhattai* and *B. megaterium* strains. In fact, in both core and pan-genome phylogeny, few strains of *B. megaterium* were found to be more related to AB211 than other *B. aryabhattai* strains (Supplementary Figure S1). Among these strains of *B. megaterium*, complete genome sequence of strain Q3, having maximum genomic similarity with AB211, and also having close relationship in both core and pan-matrix phylogeny, was being selected as a reference genome for comparative analysis. Average Nucleotide Identity (Goris et al., 2007) (ANI) value also indicated a high degree of reciprocal sequence similarity (Average ANI = 96.35%) between these two genomes (Supplementary Figure S2). This close homology of *B. megaterium* with *B. aryabhattai* strains have been well reported in the previous analyses (Ray et al., 2012), emphasizing a common evolutionary path of these two species of Bacilli.

For genomic comparisons, seven other *B. aryabhattai* genome sequences were downloaded from NCBI (**Table 1**). Genomic alignments were performed for all of these draft genomes against a complete genome sequence of *B. megaterium* strain Q3. Contigs were reordered, and pseudochromosomes were constructed for each of the draft genomes. To check the consistency of the draft genome sequences of *B. aryabhattai* strains, a whole genome-wide synteny analysis was performed both with the complete genome of *B. megaterium* Q3 and with each other (Supplementary Figure S3), applying different orthology criteria. Synteny analysis of strain AB211 with *B. megaterium* Q3 revealed an overall consistent genomic arrangement in each of these closely related species (Supplementary Figure S3A). Locally Collinear Blocks (LCB) in progressive Mauve (Darling et al., 2010) also showed no major recombination chunks among the eight strains of *B. aryabhattai* (Supplementary Figure S3B). Furthermore, a genome synteny analysis among these strains was performed with different criteria of identifying probable recombination blocks (such as *e*-value, base pair length etc.) in GSV (Revanna et al., 2011). Probable recombination regions were marked, and individual gene sequences from these regions were extracted and aligned with ClustalW in MEGA6 (Tamura et al., 2013) for recombination study with RDP (Martin et al., 2005). Three different recombination detection methods were tested in these sequences, viz. GENECONV (Sawyer, 2000), Bootscan (Martin et al., 2005) and MaxChi (Smith, 1992), but none of the above mentioned program showed any evidence of recombination in these gene alignments.

**FIGURE 2:**
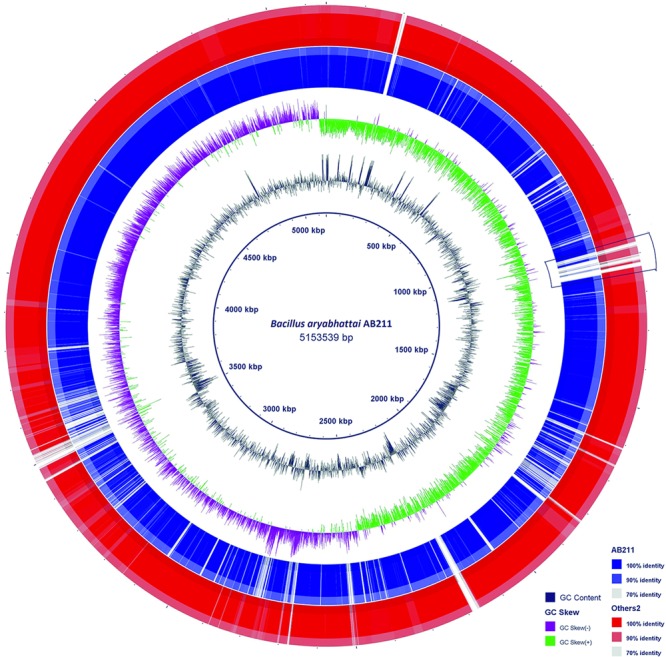
**Blast Ring Image Generator (BRIG) diagram is showing homologous chromosome segments of *B. aryabhattai* strains with *B. megaterium* strain Q3.** The *B. aryabhattai* strain AB211 is shown in the inner purple circle (AB211). The outer brown circle (others) contains combined genomic regions of other seven strains (see **Table 1**) of *B. aryabhattai* considered in our analysis. GC content and genomic GC skew are also shown in the figure. The marked area represents a stretch of exclusively present genes in AB211 when compared with other *B. aryabhattai* strains (see text for details).

By a comparative BLASTN analysis with BLAST Ring Image Generator (BRIG), the newly sequenced genome of *B. aryabhattai* strain AB211 was compared with partially assembled genomes of seven other *B. aryabhattai* strains (**Figure 2**). Complete genome sequence of *B. megaterium* strain Q3, being phylogenetically close and having maximum genome identity (Supplementary Table S2) with strain AB211 (beside type strain), was taken as a reference genome. The overall genome of AB211 has a high degree of sequence similarities with other *B. aryabhattai* strains. The GC content and overall GC skew, when compared with *B. megaterium* Q3, showed certain patches of atypical GC usage, possibly implying regions of horizontal gene transfer (HGT).

**FIGURE 3:**
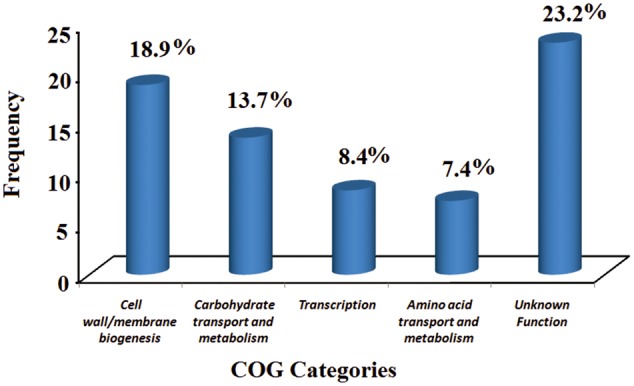
**Significantly abundant COG categories among exclusively present genes of *B. aryabhattai* AB211.** Significance levels are assessed using chi-square test (*p* < 0.05). *Y*-axis represents COG frequency values.

To further understand the gene usage of AB211, a pan-genomic analysis was carried out among eight *B. aryabhattai* strains and five evolutionary as well as genetically similar *B. megaterium* strains (**Table 1** and Supplementary Figure S4). In these 13 highly similar genomes, the core-genome constitutes of 3558 genes, whereas the pan-genome has 7392 genes (Supplementary Tables S3, S4). The power law regression in the BPGA pipeline estimated that the pan-genome of *B. aryabhattai* and *B. megaterium* was still open (Supplementary Table S4). The core genes, that were shared among all these strains, constituted of 3558 genes and the dispensable genes, which were shared by some but not all, were mainly responsible for differential characteristics of these strains. 158 gene families were found to be exclusively present in AB211 but absent in all other strains of *B. aryabhattai* (Supplementary Table S5). These 158 unique genes are probably foreign in nature and could be inherited by HGT events. GC% analyses of those genes showed that these genes have significantly lower G+C content than the genomic GC of strain AB211 (Supplementary Figure S5), supporting the probable HGT events from lower G+C content organism. Although most of these unique gene products were uncharacterized, the annotated ones were mostly related to transport of small molecules and ions, transcriptional regulators, and membrane proteins. Interestingly enough, when these genes were arranged in AB211 chromosome after contig re-ordering, they appeared to be contiguous in AB211 genome. Among these, a continuous stretch of ∼200 kbps region (**Figure 2**), encoding 85 proteins, were exclusively present in *B. aryabhattai* AB211. To functionally characterize these proteins, COGs analysis of the encoded proteins was performed, and their significant abundance levels were assessed with a 2 × 2 chi-square contingency table in STATISTICA 6.0 (StatSoft Inc., 2001). The significantly abundant COG categories (*p* < 0.05) have been shown in **Figure 3**. The most abundant COG categories among these exclusively present proteins of AB211, were COG category M (Cell wall/membrane/envelope biogenesis, *P* = 0.09), and COG category G (Carbohydrate transport and metabolism, *p* = 0.07). The other most abundant COG categories, although not statistically significant, were COG category K (Transcription), and COG category E (Amino acid transport and metabolism). Individual COG categories, and their relative abundances have been shown in Supplementary Table S6. Abundances of these specific functional categories indicate the unique functional attributes of AB211 within its distinct ecological niche, which require further thorough examination by biochemical studies.

### Biochemical Characterization of *B. aryabhattai* AB211

*Bacillus aryabhattai* AB211 is a Gram-positive rod shaped spore forming firmicute. Classical biochemical test evidenced that the strain AB211 was positive for MR- VP test and indole production, and negative for catalase, amylase and gelatinase test (Supplementary Figure S6A). Results of other biochemical tests have been summarized in Supplementary Figures S6A,B. Besides, strain AB211 showed positive motility through diffused cloudy growth away from the line of inoculation in semisolid M9 agar medium (Supplementary Figure S7D). It also showed resistance to the antibiotic tetracycline at 35 μg/ml concentration. The isolate was resistant against all the tested heavy metals at 5 mM concentration. It was found that the strain AB211 can grow in M9 medium supplemented with either of 0.5% (w/v) sucrose, D-glucose, D-fructose, D-maltose, D-galactose, L-arabinose, and D-mannitol as a sole source of carbon, which was further confirmed using GEN III MicroPlate^TM^ test. Identification of the strain AB211 on the basis of carbon source utilization pattern, using the GEN III MicroPlate^TM^ test revealed that the strain AB211 could utilize several other carbohydrates and amino acids, grow at high salt concentration (at 8%), and is resistant to several antibiotics like- rifamycin, troleandomycin, lincomycin, nalidixic acid, and aztreonam (Supplementary Figure S6B).

Phosphate solubilizing bacteria increases phosphorus uptake of crop plants by releasing insoluble and fixed forms of phosphorus from soil (Rodriguez and Fraga, 1999). After 7 days of incubation, the strain AB211 was found to form a clear zone around the point of inoculation on Pikovskaya’s agar plate, indicating phosphate solubilization. In the quantitative estimation, strain AB211 was found to solubilize 199.09 ± 0.18 μg/ml of inorganic phosphate. The pH of the broth was found to decline to 4.8 from 7.0 (control), due to the bacterial activity. Furthermore, quantitative estimation of exopolysaccharides revealed that the isolated EPS fraction from 72 h grown AB211 contained about 272.5 μg/ml of protein and 185 μg/ml of carbohydrate.

### Survival in the Plant Rhizosphere: Overview of *B. aryabhattai* AB211 Genomic Signature Linked to Experimental Evidence

The ability of *B. aryabhattai* AB211 to efficiently colonize on the surfaces of plant roots is a prerequisite for phytostimulation. Based on the genome analysis, *B. aryabhattai* AB211 seems well adopted to thrive in the plant rhizosphere as it encodes essential features required for its survival. In general, colonization of the root surfaces followed by phytostimulation involves different events: (a) movement (swimming) of bacteria toward plant root, (b) survival within the rhizospheric environment, e.g., survival in the presence of plant responses (oxidative stress and root exudates) and inter-species competition between microbial communities (antibiotic sensitivity), (c) adhesion and colonization of the root surfaces (biofilm), and finally (d) synergistic interactions with host plant (metabolic versatility) viz a viz plant growth promotion. An overview of each of these events as evident from genome analysis and supporting experimental results is presented in the subsequent section.

#### Movement (Swimming) of Bacteria toward Plant Root

In the very first step bacteria move toward plant roots either passively via soil water fluxes or actively via specific flagellar activity induced by plant-released compounds or root exudates (chemotaxis). *B. aryabhattai* AB211 is well equipped to actively move toward plant roots, the preferred site of active colonization. Its genome contains all the flagellar biosynthesis genes as well as gene products involved in chemotaxis (Supplementary Table S7). Under laboratory conditions, *B. aryabhattai* AB211 displayed a robust swimming phenotype (Supplementary Figure S7D). Its genome contains genomic islands encoding flagellar biosynthetic genes, which are very similar to those found in other *Bacillus* species (Chen et al., 2007). Inside the flagellar biosynthesis gene cluster, determinants involved in chemotaxis were identified. Interestingly, flagellar proteins are thought to elicit a host basal defense against the potential pathogen. In *B. amyloliquefaciens* FZB42, it was proposed that variation of flagellins and other exposed bacterial proteins during colonization of plant roots might enhance the ability of bacteria to tolerate unfavorable plant responses and thereby facilitate an increased competence in the rhizosphere (Chen et al., 2007). A similar scenario might be possible in case of *B. aryabhattai* AB211. Beside flagella, no other surface appendages such as pilus-like structures were evident from either the genome sequence analysis or from electron microscopic examination of strain AB211.

#### Survival within the Rhizospheric Environment

To survive within the rhizosphere, bacteria need to employ diverse defensive machinery that operate in an orchestrated manner. Plants use a variety of defense mechanisms against bacterial, viral, and fungal pathogens, including the production of reactive oxygen species (ROS) in the form of hydrogen peroxide, hydroperoxyl radicals, hydroxyl radicals, superoxide, nitric oxide, and phytoalexins (Hammond-Kosack and Jones, 1996; Zeidler et al., 2004). A prerequisite for bacterial colonization of root surfaces within such an oxidative rhizospheric environment is to mount specific, rapid and intense defense responses. In the rhizospheric environment, root exudates stimulate up-regulation of bacterial enzymes probably involved in combating oxidative stress generated by plant roots (Doornbos et al., 2012). The *B. aryabhattai* AB211 chromosome was observed to encode three superoxide dismutases (a Mn superoxide dismutase, a Fe superoxide dismutase, and a Cu-Zn superoxide dismutase), and one catalase (Supplementary Table S8). It also revealed the presence of an organic hydroperoxide resistant protein and its transcription regulator (Supplementary Table S8). Besides, a gene encoding alkyl hydroperoxide reductase subunit C, and peroxide stress regulator PerR were identified in the genome (Supplementary Table S8). *B. aryabhattai* AB211 seems to be able to detoxify free radical nitric oxide by the presence of a flavohemoprotein nitric oxide dioxygenase, and nitrate reduction gene cluster (Supplementary Tables S8, S9). Also, the AB211 chromosome seems to encode genes involved in heat shock responses, carbon starvation, osmoregulation, and other osmotic responses (Supplementary Table S8). To this end, we believe that *B. aryabhattai* AB211 genome is well equipped to thrive in the oxidative rhizospheric environment.

Beside the cross-talk with plant defense responses, a bacterium needs to cope with competing microorganisms in the plant rhizosphere. As *B. aryabhattai* AB211 colonizes plant root, it requires strategy to achieve positive selection either by inhibiting the growth of phytopathogenic bacteria or fungi by depriving them of the essential iron or by protecting itself from the action of antibiotic/bacteriocins secreted by competing microorganisms (Whipps, 2001; Berendsen et al., 2012). *B. aryabhattai* AB211 genome was observed to encode a robust framework for iron acquisition and siderophore biosynthesis (Supplementary Table S10 and Figure S7A). It also revealed the presence of a number of antibiotic resistance cassettes (bacitracin, vancomycin, tetracycline, fluoroquinolones, and beta-lactamase) to survive inter-species competition in the rhizospheric environment (Supplementary Table S11).

To survive within the native environment, bacteria acquire traits that help the organism to thrive, and its genome retains the signature for all these traits. *B. aryabhattai* AB211 was isolated from tea rhizosphere of Darjeeling district, West Bengal, India. As a pest, pathogen and weeds are severe constrains in the productivity and quality of tea, tea planters in this part of the world use a wide range of pesticides, fungicides, or microbicides to combat these problems for high quality and economic return (Bishnu et al., 2009). Though broad-spectrum chemicals offer powerful incentives, they have serious drawbacks on microbial resistance, pest resurgence, harmful effect on human health and environment, etc. Persistence of pesticides/fungicides or their undesirable residues within the tea growing soil and adjacent water bodies contribute toward shaping the resident microbial communities. Bacterial degradation of pesticides/fungicides has been reported in diverse agricultural soil (Bishnu et al., 2012; Verma et al., 2014). Also, a number of bacterial species have been isolated from soil and characterized on their potential in degrading harmful pesticide/fungicide residues (Ahmad et al., 2012; Lovecka et al., 2015; Pailan et al., 2015; Gilani et al., 2016). *B. aryabhattai* AB211 genome was found to encode several genes involved in degradation of aromatic compounds (Supplementary Table S12). Among these degradation pathways, identification of enzymes involved in biphenyl degradation, gentisate degradation, salicylate degradation, quinate degradation, etc. indicate a possible role of strain AB211 in pesticide and fungicide removal in tea rhizospheric soil (Supplementary Table S12).

The *B. aryabhattai* AB211 genome revealed genes that are putatively involved in copper (Cu), cadmium (Cd), Zinc (Zn), Cobalt (Co), and arsenic (As) resistance/mobilization (Supplementary Table S11). A genetic locus dedicated to copper resistance includes a P-type ATPase (CopA) (EC 3.6.3.4), putative copper resistance proteins CopC/CopD, and a multidrug resistance transporter of Bcr/CflA family involved in copper homeostasis (Supplementary Table S11). AB211 genome was also observed to encode an arsenic/arsenate resistance gene cassette (Supplementary Table S11). The presence of heavy metal resistance gene cassettes in strain AB211 is not unexpected, as the strain was isolated from Darjeeling tea rhizospheric soil, which was recently being shown to contain different heavy metals (Salahinejad and Aflaki, 2010; Lagad et al., 2012). The major source of heavy metals in tea soil is the irrigation with contaminated ground water. To this end, we believe that the presence of heavy metal resistance/mobilization cassettes in strain AB211 provides a selective advantage over other bacteria to survive in the rhizosphere, especially when these metals are present.

#### Adhesion and Colonization of the Root Surfaces

**FIGURE 4:**
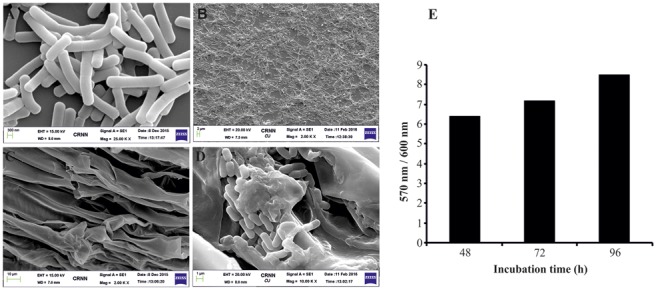
**Biofilm formation and maize root colonization of *B. aryabhattai* AB211. (A)** SEM micrograph of AB211 strain. **(B)** SEM micrograph of 96 h old static biofilm formed by AB211 strain. **(C)** SEM micrograph of control maize root **(D)** SEM micrograph of maize roots colonized by AB211 strain. **(E)** *In vitro* static biofilm formation by AB211 strain for 48, 72, and 96 h of incubation in M9 medium. The graph shows the correlation of the measured crystal violet (CV) absorbance of attached cells (OD570 nm) and the growth of the planktonic cells (OD600 nm) to emphasize some cells in a sessile lifestyle at the tested condition. Each point and the standard deviation is the mean of three independent samples per condition.

Surface attachment is a prerequisite for successful colonization of bacteria to root surfaces. In general, root colonization is believed to occur in two steps: non-specific adhesion (surface attachment), followed by firm anchoring (biofilm formation). Bacterial surface adhesion (attachment) relies on a variety of cell surface factors that allow adhesion to the host surfaces. Among the cell surface factors pili, flagella and extracellular polysaccharides play a major role in initial surface adhesion followed by biofilm formation of bacteria (Bogino et al., 2013). The *B. aryabhattai* AB211 genome does not encode proteins involved in pili-biosynthesis. However, it encoded components of flagella assembly system and for chemotaxis (Supplementary Table S7). Furthermore, AB211 genome encoded genes involved in extracellular polysaccharide biosynthesis. Flagella have been suggested to contribute in overcoming surface repulsive forces and, possibly, to alleviate in spreading of cells along a surface (Friedlander et al., 2013). Additionally, extracellular polysaccharides help to develop biofilm morphology (Irie et al., 2012). Our experimental results indicated that strain AB211 is capable of forming *in vitro* static biofilm on glass surfaces (**Figures 4A,B,E**). Furthermore, we could show that strain AB211 adheres to plant root surfaces and possibly forms biofilm-like structures (**Figures 4C,D**). Besides our biochemical analysis confirms that strain AB211 synthesizes extracellular polysaccharides. To this end, we believe strain AB211 is well equipped with machineries that help in establishing root surface association by this bacterium.

#### Synergistic Interactions with Host Plant (Metabolic Versatility) viz a viz Plant Growth Promotion

Plant roots release a wide range of carbon-containing compounds, including carbohydrates, amino acids, organic acids, phenolic compounds, fatty acids, sterol, vitamins, enzymes, purines/nucleotides as well as inorganic molecules such as HCO_3_^-^, that are collectively known as rhizodeposits (Dakora and Phillips, 2002; Carvalhais et al., 2011). To achieve a synergistic interaction with host plant, a bacterium needs to possess the metabolic potential to deal with available nutrients within the rhizospheric environment. The *B. aryabhattai* AB211 genome was found to encode various pathways of central carbohydrate metabolism, including the tricarboxylic acid cycle, the Entner-Doudoroff, the Embden-Meyerhof-Parnas and the pentose phosphate pathway (Supplementary Table S13). Also, it revealed the presence of proteins involved in the anaerobic fermentation process, and for photorespiration (Supplementary Table S13). The strain AB211 showed the ability to utilize a large variety of plant-derived compounds such as D-mannitol, sucrose, salicin, trehalose, D-mannose, L-arabinose, maltose, xylose, glucose, etc. (Supplementary Figure S6). The genome of strain AB211 does not encode proteins involved in cellulose degradation, which is consistent with its non-pathogenic behavior.

Unlike many other rhizobacteria, *B. aryabhattai* AB211 is unable to fix nitrogen and lacks required *nif* genes. However, it contained genes required for assimilatory nitrate reduction pathways (Supplementary Table S9). Genes involved in denitrification and ammonia assimilation were evident in the genome sequence as well (Supplementary Table S9). Besides, the strain AB211 seems capable in generation of nitrosative stress (Supplementary Table S9).

Microorganisms play an important role in the natural phosphorous cycling by solubilizing fixed and precipitated phosphorous in soil. In general, the phenomena of fixation and precipitation of phosphorous in agricultural soil is dependent on pH and soil type. Thus, in acid soils, e.g., in a tea plantation, phosphorus is fixed by free oxides and hydroxides of aluminum and iron, while in alkaline soils, e.g., in rice plantation, it is fixed by calcium, thereby causing a low availability of soluble phosphate (Mahdi et al., 2012). Biological solubilization of insoluble phosphate has recently attracted immense attention. Several enzymes have been shown to be involved in making insoluble phosphorous compounds available for cellular growth (Ohtake et al., 1996; Richardson and Hadobas, 1997; McGrath et al., 1998; Skraly and Cameron, 1998; Rodriguez and Fraga, 1999). These processes are achieved via the action of phosphatases, phytases, phosphonoacetate hydrolases, D-α-glycerophosphatases, and C-P lyases. The *B. aryabhattai* AB211 genome encodes potential candidates representing exopolyphosphatase (EC 3.6.1.11), manganese-dependent inorganic pyrophosphatase (EC 3.6.1.1) and an alkaline phosphatase (EC 3.1.3.1) (Supplementary Table S14). Besides, some genes involved in transport and assimilation of inorganic phosphate (Pho regulon) were identified as well (Supplementary Table S14). Experiments in our group have confirmed that strain AB211 is capable of solubilizing insoluble inorganic phosphate compounds, such as tri-calcium phosphate and rock phosphate.

Plant growth promoting rhizobacteria often enhance plant growth through the synthesis of the plant auxin IAA. In general, biosynthesis of bacterial IAA occurs either by tryptophan-dependent or independent manners. Biosynthesis of IAA from tryptophan has been documented in many different bacterial strains (Spaepen and Vanderleyden, 2011). It follows three major alternative pathways: indole pyruvate, tryptamine, and indole-3-acetamide (Spaepen and Vanderleyden, 2011). Experimental results showed that *B. aryabhattai* AB211 synthesizes IAA with and without the addition of tryptophan in the medium (Supplementary Figure S7B). While IAA production was higher in the presence of tryptophan, measurable quantity of IAA was also detected in the absence of tryptophan (Supplementary Figure S7B). The *B. aryabhattai* AB211 genome encodes all the genes required for biosynthesis of tryptophan from chorismate (Supplementary Figure S8 and Table S15). However, no downstream genes possibly involved in IAA production from tryptophan were identified (Supplementary Figure S8). Furthermore, analysis of the genome revealed the presence of a putative nitrilase (EC 3.5.5.1) indicating the existence of a possible tryptophan-independent IAA biosynthetic pathway in strain AB211 (Supplementary Figure S8 and Table S15). We believe that in the presence of tryptophan in the medium, being a feedback inhibitor of its biosynthesis, an excess amount of tryptophan available in the cell possibly channelizes anthranilate toward biosynthesis of IAA via production of indole-3-acetonitrile (Supplementary Figure S8). However, further studies are necessary to confirm such a proposal.

**FIGURE 5:**
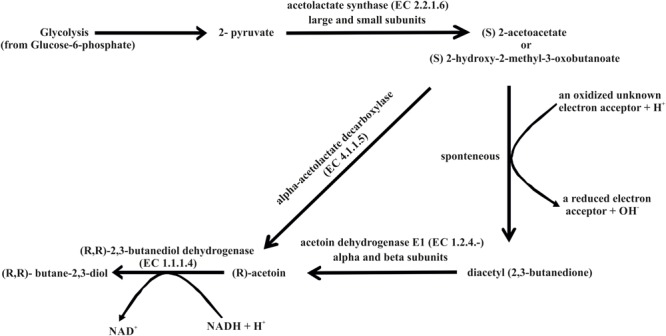
**Biosynthesis of acetoin and butane-2,3-diol based on the annotation of the AB211 genome**.

A blend of volatile compounds, especially 3-hydroxy-2-butanone (acetoin) and 2,3-butanediol, are disembogued by some of the most efficient PGPR to enhance plant growth (Ryu et al., 2003). Previous plant growth promotion studies using *B. subtilis* GB03 and *B. amyloliquefaciens* IN937a revealed that both these strains were capable of promoting plant growth utilizing volatile compounds such as 3-hydroxy-2-butanone (acetoin) and 2,3-butanediol (Ryu et al., 2003). Besides, these volatiles have been implicated in eliciting induced systemic resistance by both these *Bacillus* species (Ryu et al., 2003). The *B. aryabhattai* AB211 genome carries all the necessary components essential for the biosynthesis of both these volatile compounds (Supplementary Table S15). The major pathway for the production of acetoin and 2,3-butanediol by strain AB211 is via formation of (S) 2-acetolactate (**Figure 5**). Depending on availability of oxygen, (S) 2-acetolactate is converted either directly to (R) acetoin using enzyme alpha-acetolactate decarboxylase (EC 4.1.1.5) or is spontaneously converted into diacetyl (2,3-butanedione) which in turn can be converted into acetoin by acetoin dehydrogenase (EC 1.2.4.-) (**Figure 5**). Acetoin is either released by the bacteria or subsequently converted into 2,3-butanediol by the action of (R, R)-2,3-butanediol dehydrogenase (EC 1.1.1.4) (**Figure 5**). Although the major pathway for biosynthesis of 3-hydroxy-2-butanone (acetoin) and 2,3-butanediol is identified, further biochemical analysis is required to understand their roles in plant growth promotion by *B. aryabhattai* AB211.

The plant growth promotion studies employing *B. aryabhattai* AB211 revealed that the application of strain AB211 increased plant growth parameters of maize seedling and was found to be statistically significant (*P* = ±0.05) (**Table 2** and Supplementary Figure S7C). Experiments showed an increase in all the parameters like shoot length (73%), root length (50%), fresh weight (122%), and dry weight (70%), total chlorophyll content (136%) over the uninoculated control. Furthermore, the ability of strain AB211 to survive in soil was assessed primarily based on the cumulative cfu data recorded as on day-7 and 15 from the plating on NA (Nutrient Agar). The survival of AB211 was assessed based on the difference in cfu keeping the day-0 cfu in soil as the reference point (Supplementary Figure S9).

**Table 2:** Effect of inoculation of *B. aryabhattai* AB211 on maize seedlings.

Plant sample	Root length (cm)	Shoot length (cm)	Wet weight (gm)	Dry weight (gm)	Total chlorophyll content (mg)
Control^£^	22.83 ± 1.34	22.8 ± 1.14	1.27 ± 0.23	0.15 ± 0.02	277.61 ± 11.7
Treated^£^	34.27 ± 0.50	39.43 ± 0.49	2.83 ± 0.11	0.27 ± 0.02	656.35 ± 39.73

*^£^Values are means of sixteen independent replicates ± standard deviation. Control was used without culture.*

## Conclusion

Plant roots host a wide variety of microorganisms, many of them cooperating with the plant by providing support for plant nutrition, stress tolerance, and health. Several different modes of action are documented in these PGPR. *B. aryabhattai* AB211 genome contains many of the signature genes that are functionally linked to the plant growth promotion trait. In general, genome analyses, as well as experimental studies, confirm that *B. aryabhattai* AB211 can solubilize inorganic phosphate, synthesize siderophores, and produce IAA. Besides, genome analysis also confirms its ability to survive the oxidative, heavy metal, and antibiotic stresses imposed within the rhizospheric micro-niche. Furthermore, the AB211 genome encodes necessary arsenal required for adhesion to host root surfaces. Our experimental studies have confirmed that strain AB211 is capable of adhering to root surfaces and promotes plant growth by synthesizing IAA and other volatiles. AB211 forms biofilm under static condition and also produces extracellular polysaccharides (EPS) necessary for optimal colonization. Besides, strain AB211 possesses a complete set of chemotaxis genes and metabolic versatility to utilize plant root exudates. Our comparative genome analysis revealed that strain AB211 shares about 3558 conserved genes with other *B. aryabhattai* strains. All the genes related to plant growth promotion attributes were found to be conserved across all the *B. aryabhattai* genomes. *B. aryabhattai* strain AB211 has 158 exclusively present genes, most of them have uncharacterized function but few of these gene products were found to be involved in the transport of small molecules and ions, transcriptional regulators and membrane proteins, etc. To this end, we believe plant growth promotion trait is common for all the *B. aryabhattai* strains, but only tested for a few. Together, the presence of these features makes *B. aryabhattai* an excellent microorganism for utilization in agriculture. More studies are necessary to firmly establish the molecular mechanism of plant growth promotion by strain AB211 and its possible usefulness in a less controlled environment.

## Author Contributions

AG conceived the project and collected the rhizosphere soil sample. CB isolated the bacterial strain, performed biochemical, microbiological and plant growth promotion experiments, and prepared the strain for the sequencing analysis. IM and SM helped in electron microscopic analysis and plant growth promotion studies. AG, UB, and BB performed the comparative genomics analysis. AG, CB, and UB wrote the manuscript. All authors read and approved the final manuscript.

## Conflict of Interest Statement

The authors declare that the research was conducted in the absence of any commercial or financial relationships that could be construed as a potential conflict of interest.

## Footnotes

1 ftp.ncbi.nlm.nih.gov/

2 http://www.ncbi.nlm.nih.gov/nucleotide
