# Supplementary material for: Genome-Guided Insights into the Plant Growth Promotion Capabilities of the Physiologically Versatile Bacillus aryabhattai Strain AB211
Source: Front Microbiol. 2017 Mar 21;8:411. doi: 10.3389/fmicb.2017.00411 (PMC5359284; doi:10.3389/fmicb.2017.00411)
Supplement: Supplementary file 1 [file Data_Sheet_1.pdf]

Genome-guided insights into the plant growth promotion capabilities of the physiologically versatile *Bacillus aryabhattai* strain AB211

Chandrima Bhattacharyya, Utpal Bakshi, Ivy Mallick, Shayantan Mukherji, Biswajit Bera, Abhrajyoti Ghosh

Supplementary Figures

Content

Supplementary Figure S1: Phylogenetic analysis using A) Core genome SNP and B) Pan-genomic binary data matrix. Core and Pan Genome phylogeny are constructed in BPGA pipeline with default setting. Bootstrap values are also indicated in both trees.

Supplementary Figure S2: Average nucleotide identity (ANI) using A) best hits (one-way ANI) and B) reciprocal best hits (two-way ANI) between genome sequences of *Bacillus aryabhattai* AB211 and *Bacillus megaterium* Q3. Mean and median values of identity and bit score distribution are also shown in the figure.

Supplementary Figure S3: Genome wide syntany analysis of A) *Bacillus aryabhattai* AB211 with complete genome sequences of *Bacillus megaterium* Q3. The different window length and parameters chosen for identifying syntanic blocks are also given; and B) LCB analysis in progressive MAUVE showing locally collinear blocks among genomes of *Bacillus aryabhattai*.

Supplementary Figure S4: The Core and Pan-Genomes of *B. aryabhattai* as compared with *B. megaterium strains.* Number of shared or core genes (pink) and total number of genes or pan-genome (green) curve for 13 strains. The upper and lower edges of the boxes indicate the first quartile (25th percentile of the data) and third quartile (75th percentile), respectively, of 100 random different input orders of the genomes. The central horizontal line indicates the sample median (50th percentile). At 13 sequenced genomes, the core-genome constitutes of 3558 genes, whereas the pan-genome has 7392 total genes.

Supplementary Figure S5: GC% of *B. aryabhattai* strain AB211. GC% of individual genes are shown in blue line, whereas the red line indicate genomic GC% of *B. aryabhattai* strain AB211.

Supplementary Figure S6: (A) Landscape of metabolic versatility of *Bacillus aryabhattai* AB211. Qualitative characteristics: carbon source/ e^- donor, antibiotics, heavy metals, phosphate solublization, siderophore production, EPS synthesis, IAA production, motility and other biochemical characteristics as per

Bergey's manual. (B) Biolog assay for strain AB211. 71 carbon sources and 23 chemical utilization characteristics were examined in this study. Presence of a positive reaction was assigned with the colour green, and negative reaction was assigned a colour red.

Supplementary Figure S7: (A) Siderophore production by *B. aryabhattai* AB211. (B) IAA production at different concentrations of tryptophan by strain AB211. (C) Plant growth promotion activity of strain AB211 using maize seedlings. (D) Motility of strain AB211.

Supplementary Figure S8: Synthesis of IAA based on the annotation of the AB211 genome.

Supplementary Figure S9: Extent of *B. aryabhattai* AB211 cfu in rhizospheric soil mix on different sampling dates commencing from the date of AB211 application (day-0) as per the cfu estimations on selective NA (cumulative of cfu recorded on day-0, day-7, and day-15; vertical bars indicate standard deviation).

Supplementary Figure S1: Phylogenetic analysis using A) Core genome SNP and B) Pan-genomic binary data matrix. Core and Pan Genome phylogeny are constructed in BPGA pipeline with default setting. Bootstrap values are also indicated in both trees.

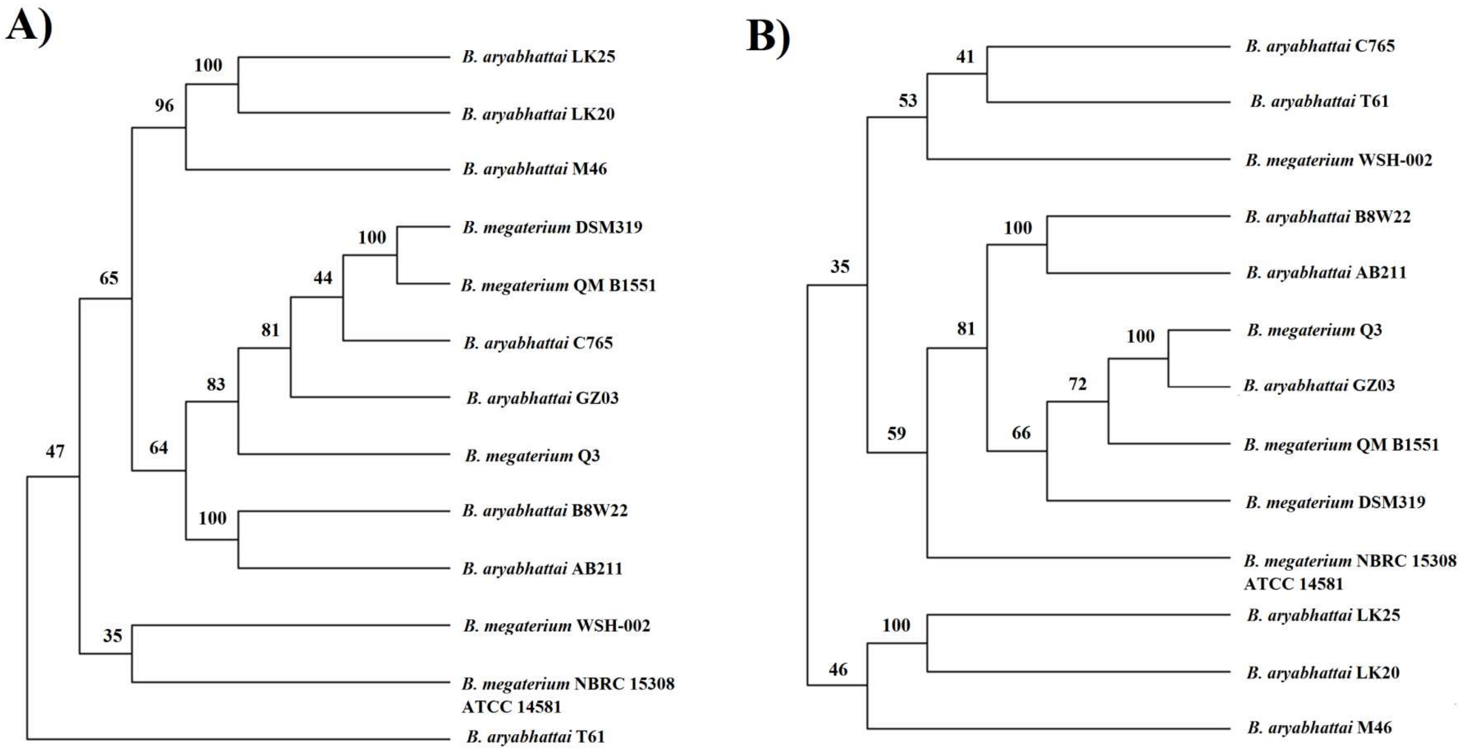

Supplementary Figure S2: Average nucleotide identity (ANI) using A) best hits (one-way ANI) and B) reciprocal best hits (two-way ANI) between genome sequences of *Bacillus aryabhattai* AB211 and *Bacillus megaterium* Q3. Mean and median values of identity and bit score distribution are also shown in the figure.

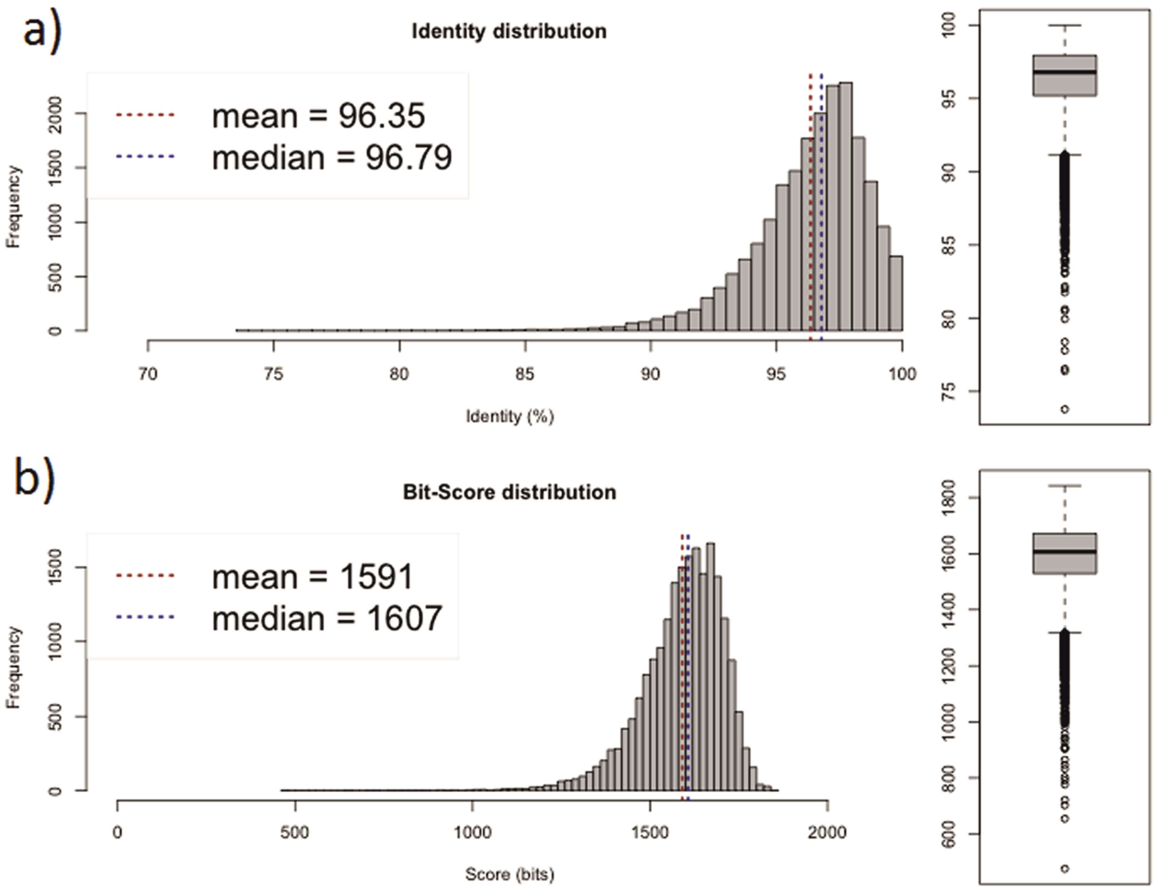

Supplementary Figure S3: Genome wide syntany analysis of A) *Bacillus aryabhattai* AB211 with complete genome sequences of *Bacillus megaterium* Q3. The different window length and parameters chosen for identifying syntanic blocks are also given; and B) LCB analysis in progressive MAUVE showing locally collinear blocks among genomes of *Bacillus aryabhattai*.

Supplementary Figure S3A

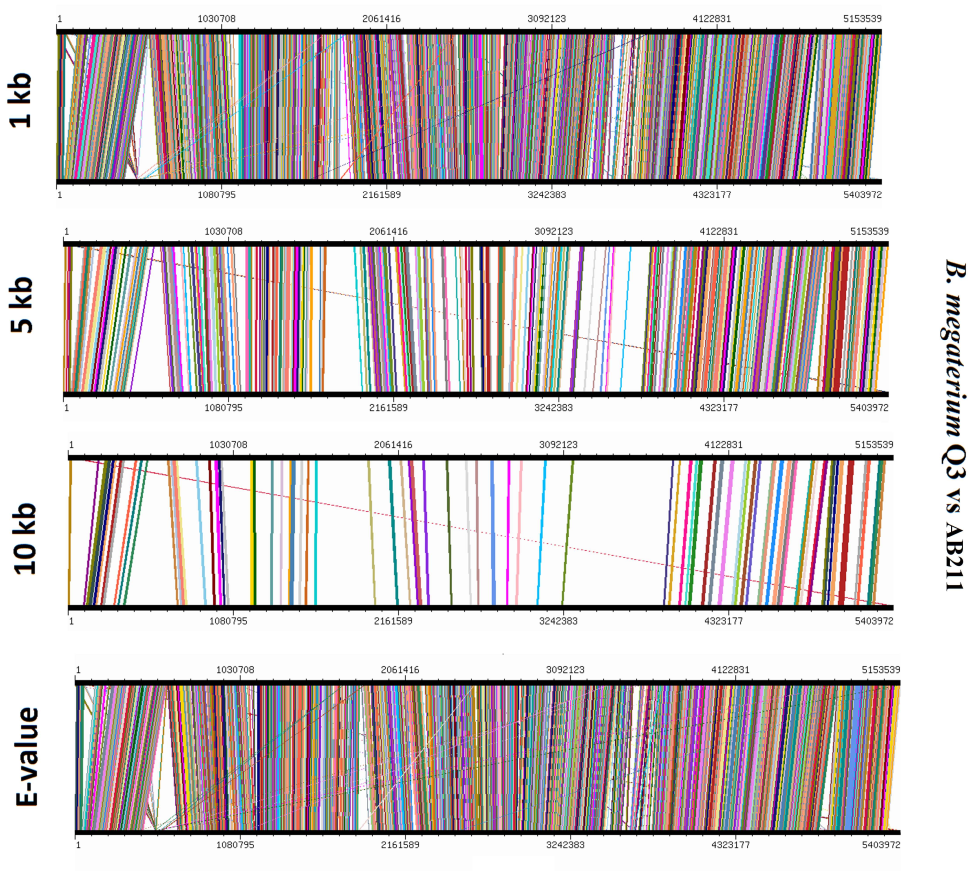

Supplementary Figure S3B

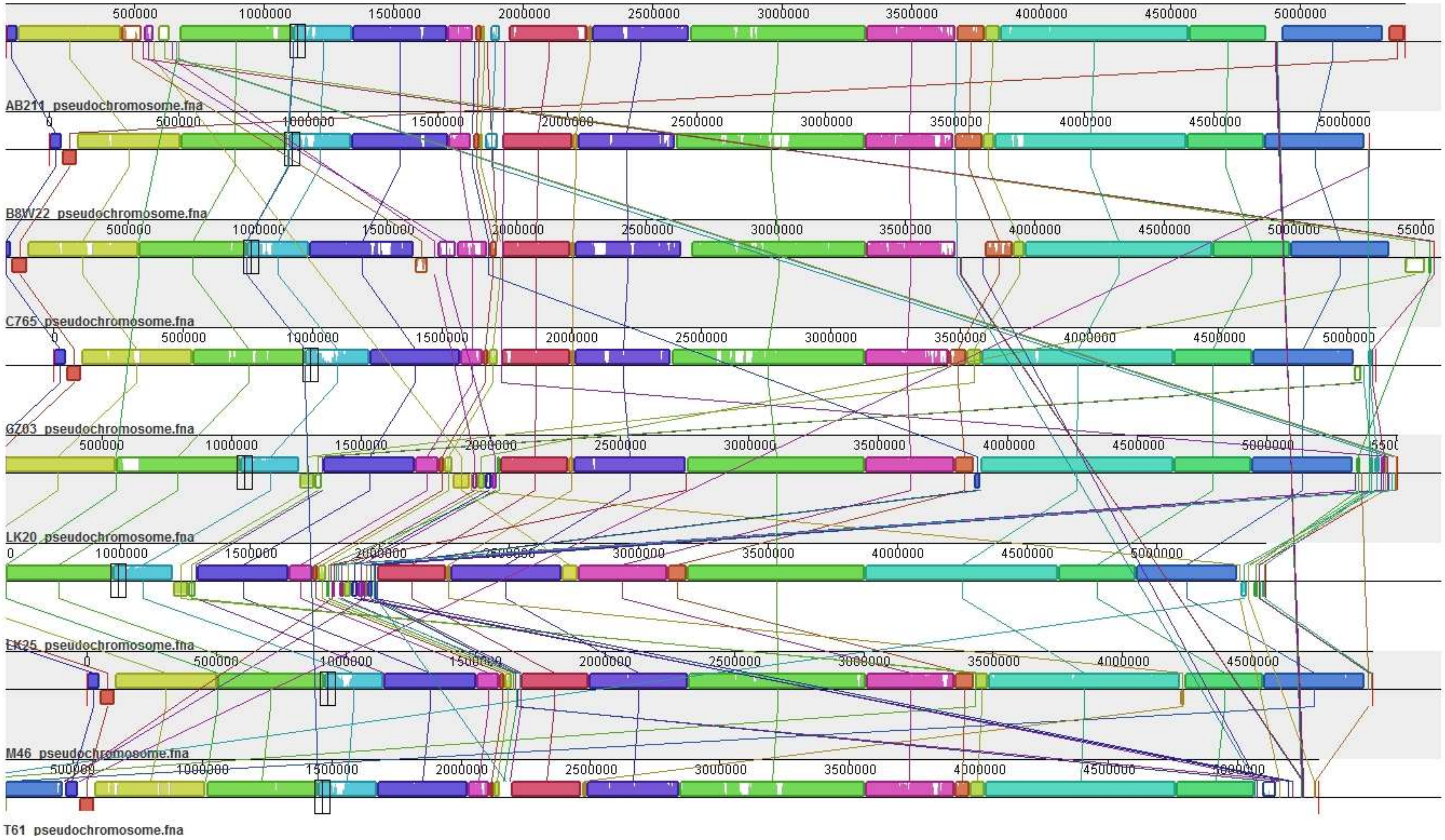

Supplementary Figure S4: The Core and Pan-Genomes of *B. aryabhattai* as compared with *B. megaterium strains.* Number of shared or core genes (pink) and total number of genes or pan-genome (green) curve for 13 strains. The upper and lower edges of the boxes indicate the first quartile (25th percentile of the data) and third quartile (75th percentile), respectively, of 100 random different input orders of the genomes. The central horizontal line indicates the sample median (50th percentile). At 13 sequenced genomes, the core-genome constitutes of 3558 genes, whereas the pan-genome has 7392 total genes.

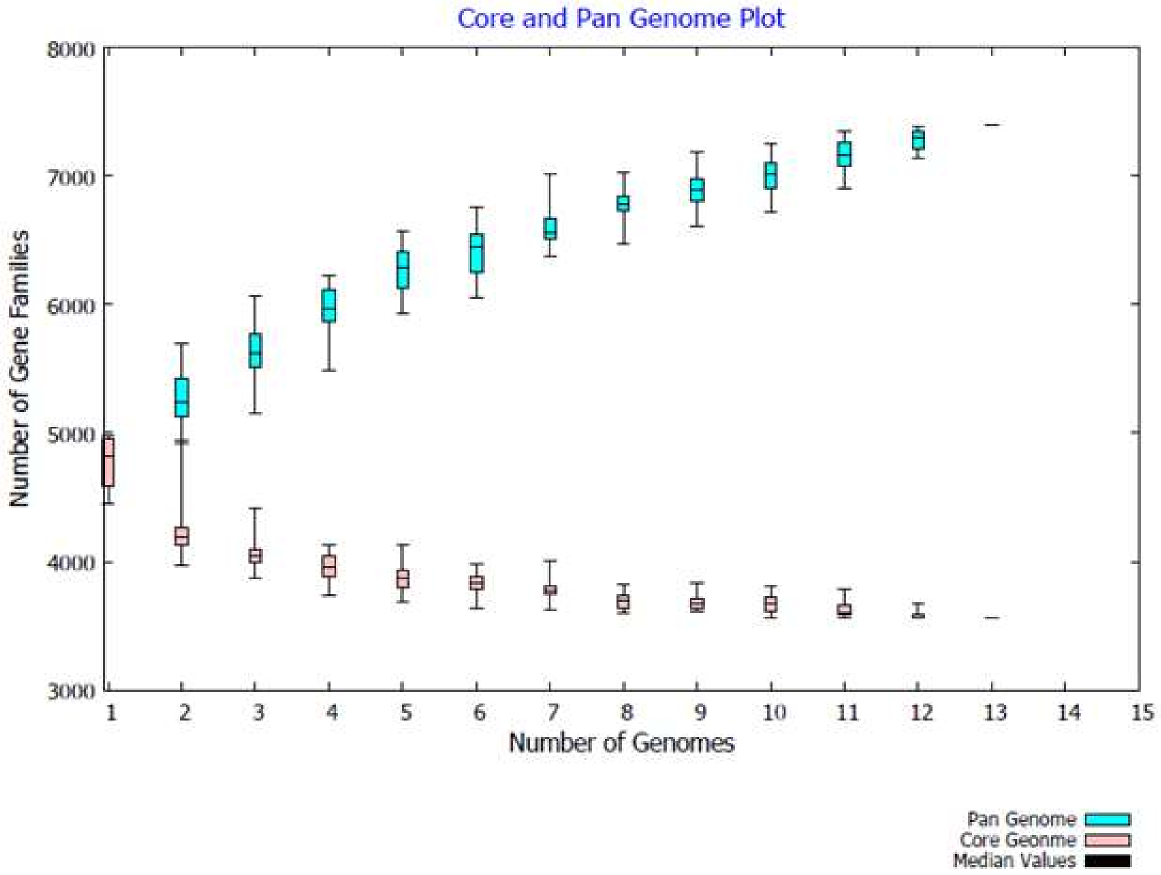

Supplementary Figure S5: GC% of *B. aryabhattai* strain AB211. GC% of individual genes are shown in blue line, whereas the red line indicate genomic GC% of *B. aryabhattai* strain AB211

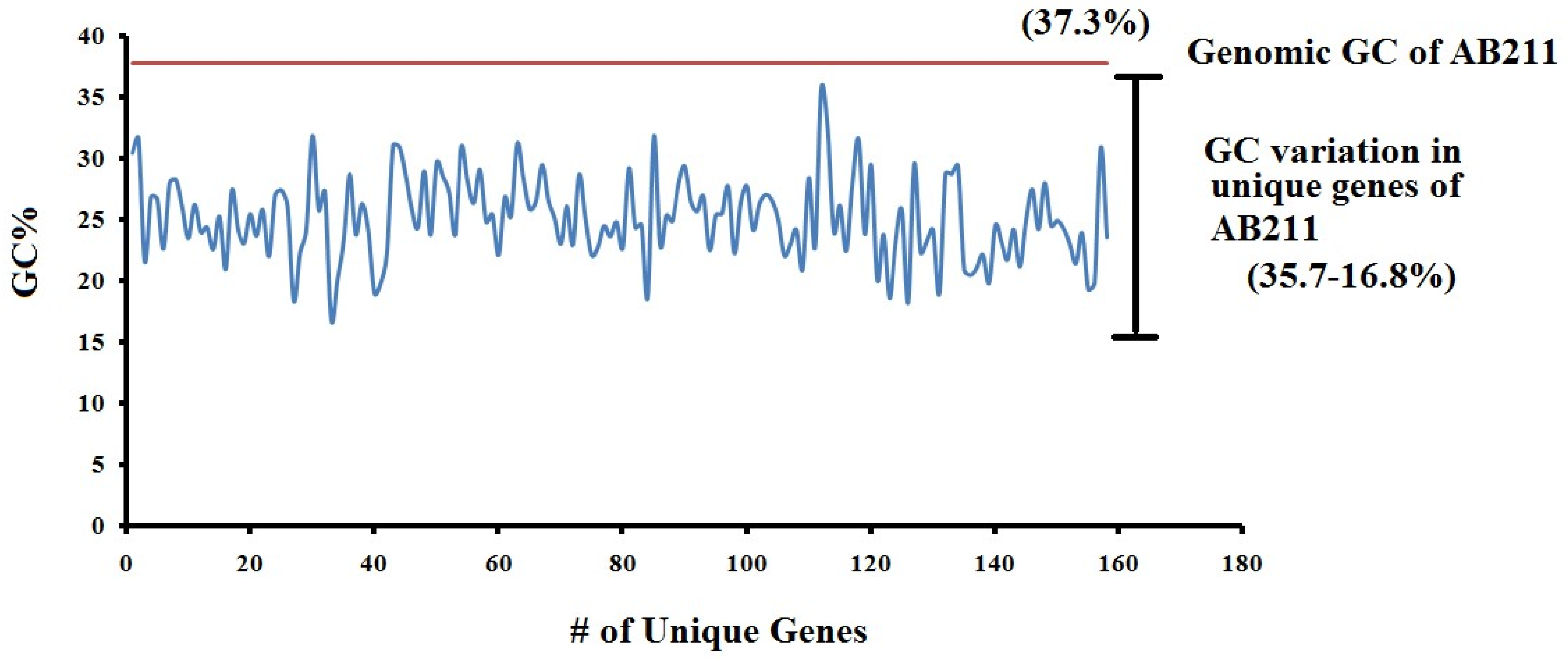

Supplementary Figure S6: (A) Landscape of metabolic versatility of *Bacillus aryabhattai* AB211. Qualitative characteristics: carbon source/ e^- donor, antibiotics, heavy metals, phosphate solublization, siderophore production, EPS synthesis, IAA production, motility and other biochemical characteristics as per Bergey's manual. (B) Biolog assay for strain AB211. 71 carbon sources and 23 chemical utilization characteristics were examined in this study. Presence of a positive reaction was assigned with the colour green, and negative reaction was assigned a colour red.

A

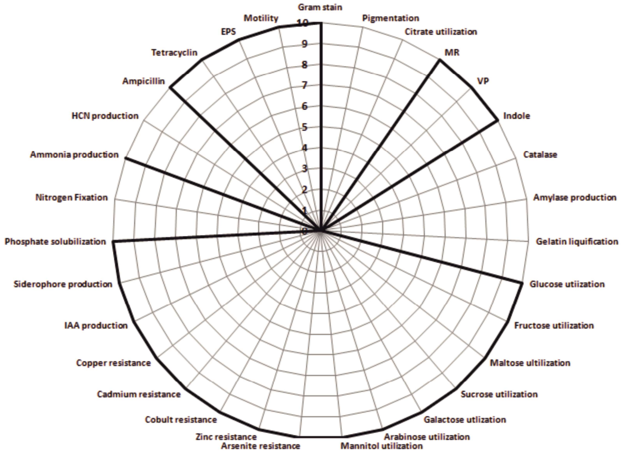

B

Negative control	Dextrin	D-Maltose	D-Trehalose	D-Cellobiose	Gentiobiose	Sucrose	D-Turanose	Stachyose	Positive Control	pH 6	pH 5
D-Raffinose	α–D-Lactose	D-Melibiose	β-Methyl-D-Glucoside	D-Salicin	N-Acetyl-D-Glucosamine	N-Acetyl-β-D-Mannosamine	N-Acetyl-D-Galactosamine	N-Acetyl-Neuraminic Acid	1%NaCl	4% NaCl	8% NaCl
α-D-Glucose	D-Mannose	D-Fructose	D-Galactose	3-Methyl Glucose	D-Fucose	L-Fucose	L-Rhamnose	Inosine	1% Sodium Lactate	Fusidic Acid	D-Serine
D-Sorbitol	D-Mannitol	D-Arabitol	Myo-Inositol	Glycerol	D-Glucose-6-PO4	D-Fructose-6-PO4	D-Aspartic Acid	D-Serine	Troleandomycin	Rifamycin SV	Minocycline
Gelatin	Glycyl-L—Proline	L-Alanine	L-Arginine	L-Aspartic Acid	L-Glutamic Acid	L-Histidine	L-Pyroglutamic Acid	L-Serine	Lincomycin	Guanidine HCl	Niaproof 4
Pectin	D-Galacturonic Acid	L-Galactonic Acid Lactone	D-Gluconic Acid	D-Glucuronic Acid	Glucuronamide	Mucic Acid	Quinic Acid	D-Saccharic Acid	Vancomycin	Tetrazolium Violet	Tetrazolium Blue
p-Hydroxy-Phenylacetic acid	Methyl Pyruvate	D-Lactic acid Methyl Ester	L-Lactic Acid	Citric Acid	α-Keto-Glutaric Acid	D-Malic Acid	L-Malic Acid	Bromo-Succinic Acid	Nalidixic Acid	Lithum Chloride	Pottasium Tellurite
Tween-40	Γ-Amino-Butyric Acid	α-Hydroxy-Butyric Acid	β-Hydroxy-D,L-Butyric Acid	A-Keto-Butyric Acid	Acetoacetic Acid	Propionic Acid	Acetic Acid	Formic Acid	Aztreonam	Sodium Butyrate	Sodium Bromate

No growth

Weak growth

Growth

Supplementary Figure S7: (A) Siderophore production by *B. aryabhattai* AB211. (B) IAA production at different concentrations of tryptophan by strain AB211. (C) Plant growth promotion activity of strain AB211 using maize seedlings. (D) Motility of strain AB211.

A

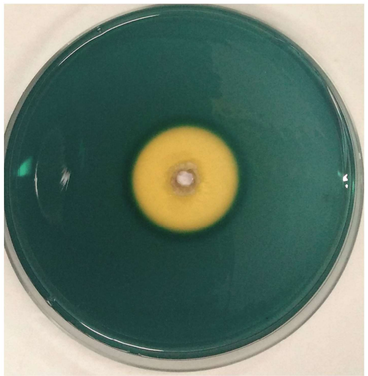

B

L-tryptophan concentration (μg/ml)	Amount of IAA produced (μg/ml)
Trp0	6.55±0.42
Trp50	10.46±0.89
Trp100	14.16±2.78
Trp200	17.68333±3.7
Trp400	33.7±4.62
Trp500	43.98±9.45

C

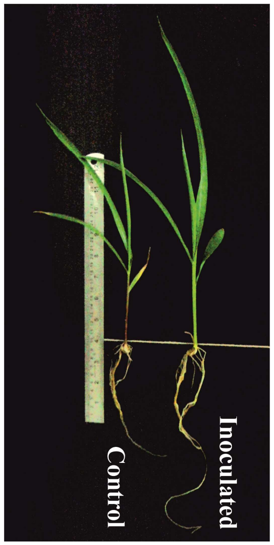

D

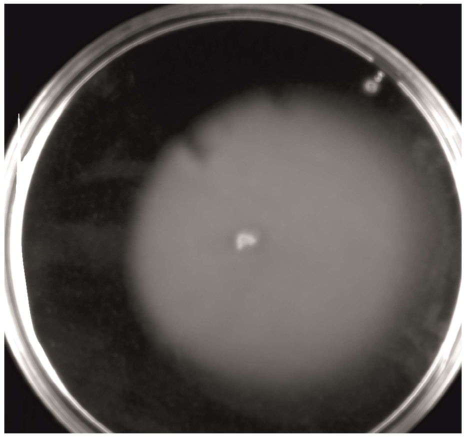

Supplementary Figure S8: Synthesis of IAA based on the annotation of the AB211 genome

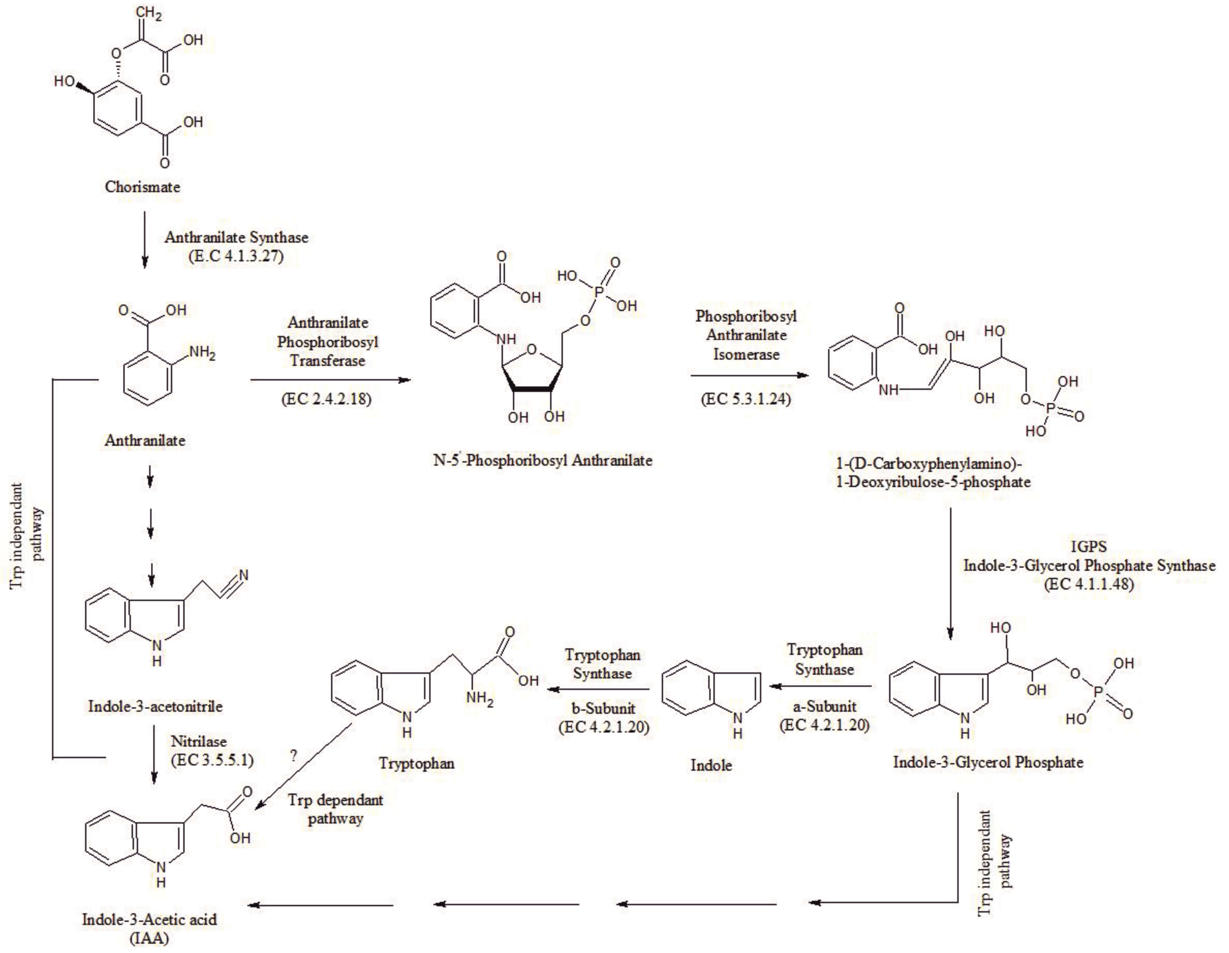

Supplementary Figure S9: Extent of *B. aryabhattai* AB211 cfu in rhizospheric soil mix on different sampling dates commencing from the date of AB211 application (day-0) as per the cfu estimations on selective NA (cumulative of cfu recorded on day-0, day-7, and day-15; vertical bars indicate standard deviation).

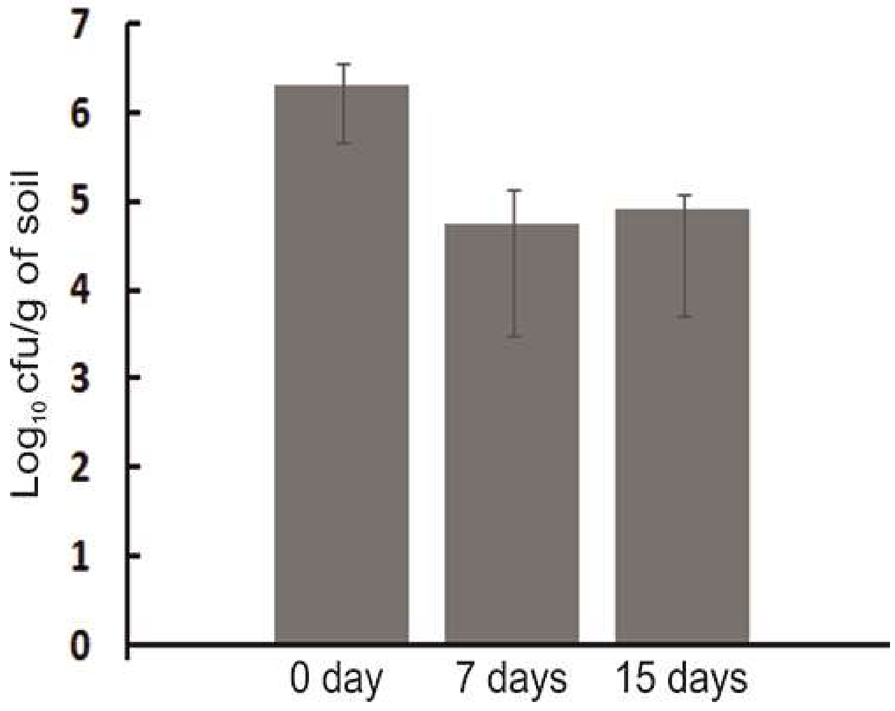

Supplementary Tables

Content

Supplementary Table S1: Metabolic diversity with respect to plant-microbe interaction of *B. aryabhattai* AB211

Supplementary Table S2: Average Nucleotide Identity (ANI) values during genomic comparison of B. *aryabhattai* strain AB211 with other homologous strains

Supplementary Table S3: Number of core, accessory, unique and exclusively absent genes as obtained from Pan-genome analysis of 13 strain of *B. aryabhattai and B. megaterium.*

Supplementary Table S4: Power and exponential fit law in BPGA pipeline to find out the estimated size of core genome and open/close nature of pan-genome

Supplementary Table S5: Exclusively present proteins of *Bacillus aryabhattai* AB211

Supplementary Table S6: COG category wise classification of 84 unique proteins of *B.aryabhattai* AB211

Supplementary Table S7: Motility and signaling genes in *B.aryabhattai* AB211

Supplementary Table S8: Stress response genes in *B.aryabhattai* AB211

Supplementary Table S9: Nitrogen metabolism genes in *B.aryabhattai* AB211

Supplementary Table S10: Siderophores and iron acquisition genes in *B.aryabhattai* AB211

Supplementary Table S11: Antibiotic and heavy metal resistance genes in *Bacillus aryabhattai* AB211

Supplementary Table S12: Aromatic compound degradation pathway genes in *B.aryabhattai* AB211

Supplementary Table S13: Carbohydrate metabolism in *B.aryabhattai* AB211

Supplementary Table S14: Phosphorous metabolism genes in *B.aryabhattai* AB211

Supplementary Table S15: Tryptophan/Auxin/Acetoin/2,3-butandiol biosynthesis genes in *B.aryabhattai* AB211

Table S1. Metabolic diversity with respect to plant-microbe interaction of *B. aryabhattai* AB211

No.	Metabolic pathways	Number of genes
1	Carbohydrate metabolism	300
2	Flagella assembly, function and signaling	54
3	Stress response	74
4	Nitrogen metabolism	19
5	Siderophore biosynthesis and iron acquisition	29
6	Antibiotic and heavy metal resistance	43
7	Aromatic compound degradation	16
8	Phosphorous metabolism	19
9	Plant growth promoting hormones and volatiles	17

Supplementary Table S2: Average Nucleotide Identity (ANI) values during genomic comparison of B. *aryabhattai* strain AB211 with other homologous strains

		B. aryabhattai AB211
Sl.No.	Strains	ANI [1]
1	*B. aryabhattai* B8W22	97.78
2	*B. aryabhattai* C765	95.21
3	*B. aryabhattai* GZ03	96.34
4	*B. aryabhattai* LK20	96.25
5	*B. aryabhattai* LK25	96.21
6	*B. aryabhattai* M46	96.31
7	*B. aryabhattai* T61	96.31
8	*B. megaterium* Q3	96.35
9	*B. megaterium* QM B1551	95.47
10	*B. megaterium* NBRC 15308 ATCC 14581	95.48
11	*B. megaterium* DSM319	95.45
12	*Bacillus megaterium* WSH-002	95.45

1. Rodriguez-R, Luis M., and Konstantinos T. Konstantinidis. "Bypassing cultivation to identify bacterial species." Microbe 9.3 (2014): 111-8.

Supplementary Table S3: Number of core, accessory, unique and exclusively absent genes as obtained from Pan-genome analysis of 13 strains of *B. aryabhattai and B. megaterium.*

Sl. No.	Organism name	# core genes	# accessory genes	# unique genes	# exclusively absent genes
1	*B. aryabhattai* AB211	3558	1173	158	11
2	*B. aryabhattai* B8W22	3558	866	130	104
3	*B. aryabhattai* C765	3558	1188	232	10
4	*B. aryabhattai* GZ03	3558	1117	80	5
5	*B. aryabhattai* LK20	3558	1394	35	1
6	*B. aryabhattai* LK25	3558	1391	2	1
7	*B. aryabhattai* M46	3558	985	40	8
8	*B. aryabhattai* T61	3558	1113	151	8
9	*B. megaterium* Q3	3558	1159	249	9
10	*B. megaterium* QM B1551	3558	998	71	28
11	*B. megaterium* NBRC 15308 ATCC 14581	3558	1043	214	32
12	*B. megaterium* DSM319	3558	938	44	63
13	*B. megaterium WSH-002*	3558	798	92	114

Supplementary Table S4: Power and exponential fit law in BPGA pipeline to find out the estimated size of core genome and open/close nature of pan-genome

	PAN GENOME	CORE GENOME
Fit law	POWER	EXPONENTIAL
Equation	f(x)=a.x^b	f1(x)=c.e^(d.x)
Parameters	a= 4688.9	c= 4368.1
	b= 0.175829	d= -0.0190245
Expected Size	7392	0
Estimated Size	7360.96	3411.01

The parameter 'b' = 0.175829 , The pan genome is still open but may be closed soon.

Supplementary Table S5: Exclusively present proteins of *Bacillus aryabhattai* AB211

**Protein_ID**	**Description**
WP_013081988.1	MULTISPECIES: MFS transporter
WP_028412222.1	MULTISPECIES: CoA transferase
WP_071271916.1	hypothetical protein
WP_071272047.1	alpha/beta hydrolase
WP_071272118.1	aspartate racemase
WP_071272227.1	Phosphoribosyl-amino-imidazole carboxylase
WP_071272272.1	hypothetical protein
WP_071273744.1	hypothetical protein
WP_071273749.1	hypothetical protein
WP_071273871.1	hypothetical protein
WP_071275060.1	hypothetical protein
WP_071275063.1	hypothetical protein
WP_071275066.1	hypothetical protein
WP_071275069.1	hypothetical protein
WP_071275072.1	hypothetical protein
WP_071275075.1	hypothetical protein
WP_071275078.1	hypothetical protein
WP_071275081.1	hypothetical protein
WP_071275084.1	hypothetical protein
WP_071275087.1	hypothetical protein
WP_071275090.1	hypothetical protein
WP_071275093.1	hypothetical protein
WP_071275096.1	hypothetical protein
WP_071275426.1	hypothetical protein
WP_071275428.1	sigma-54-dependent Fis family transcriptional regulator
WP_071275430.1	hypothetical protein

WP_071275466.1	hypothetical protein
WP_071275485.1	hypothetical protein
WP_071275487.1	hypothetical protein
WP_071275534.1	nuclear export factor GLE1
WP_071275536.1	copper transporter
WP_071275556.1	transcriptional regulator
WP_071275599.1	hypothetical protein
WP_071275949.1	hypothetical protein
WP_071275954.1	hypothetical protein
WP_071275958.1	aspartate racemase
WP_071275960.1	LysR family transcriptional regulator
WP_071276429.1	hypothetical protein
WP_071276430.1	hypothetical protein
WP_071276431.1	hypothetical protein
WP_071276432.1	hypothetical protein
WP_071276433.1	hypothetical protein
WP_071276603.1	AP endonuclease
WP_071276607.1	hydroxymethylglutaryl-CoA lyase
WP_071276609.1	shikimate dehydrogenase
WP_071277104.1	hypothetical protein
WP_071277163.1	hypothetical protein
WP_071277168.1	hypothetical protein
WP_071277170.1	hypothetical protein
WP_071277180.1	transcriptional regulator
WP_071277182.1	PTS sorbitol transporter subunit IIA
WP_071277184.1	PTS sorbitol transporter subunit IIC
WP_071277186.1	transcriptional regulator
WP_071277188.1	NAD(P)-dependent oxidoreductase
WP_071277190.1	NUDIX hydrolase

WP_071277191.1	hypothetical protein
WP_071277193.1	hypothetical protein
WP_071277198.1	hypothetical protein
WP_071277213.1	hypothetical protein
WP_071277217.1	hypothetical protein
WP_071277222.1	MFS transporter
WP_071277225.1	hypothetical protein
WP_071277226.1	hypothetical protein
WP_071277227.1	transcriptional regulator
WP_071277231.1	hypothetical protein
WP_071277232.1	nitrite reductase
WP_071277233.1	MFS transporter
WP_071277234.1	PTS beta-glucoside transporter subunit EIIBCA
WP_071277236.1	hypothetical protein
WP_071277237.1	transcription antiterminator BglG
WP_071277238.1	hypothetical protein
WP_071277239.1	hypothetical protein
WP_071277241.1	nitroreductase A
WP_071277246.1	hypothetical protein
WP_071277251.1	hypothetical protein
WP_071277252.1	hypothetical protein
WP_071277253.1	hypothetical protein
WP_071277254.1	hypothetical protein
WP_071277255.1	hypothetical protein
WP_071277256.1	hypothetical protein
WP_071277257.1	hypothetical protein
WP_071277258.1	hypothetical protein
WP_071277259.1	hypothetical protein
WP_071277280.1	hypothetical protein

WP_071277293.1	PTS sorbitol transporter subunit IIB
WP_071277296.1	hypothetical protein
WP_071277303.1	hypothetical protein
WP_071277304.1	DNA polymerase subunit beta
WP_071277307.1	hypothetical protein
WP_071277308.1	hypothetical protein
WP_071277311.1	antibiotic biosynthesis monooxygenase
WP_071277312.1	hypothetical protein
WP_071277320.1	hypothetical protein
WP_071277322.1	hypothetical protein
WP_071277323.1	hypothetical protein
WP_071277324.1	spore coat protein
WP_071277330.1	pseudaminic acid synthase
WP_071277331.1	UDP-4-amino-4,6-dideoxy-N-acetyl-beta-L-altrosamine N-acetyltransferase
WP_071277332.1	UDP-2,4-diacetamido-2,4,6-trideoxy-beta-L-altropyranose hydrolase
WP_071277333.1	gluconate 5-dehydrogenase
WP_071277334.1	oxidoreductase
WP_071277335.1	spore coat protein
WP_071277336.1	UDP-4-amino-4,6-dideoxy-N-acetyl-beta-L-altrosamine transaminase
WP_071277337.1	NAD-dependent dehydratase
WP_071277338.1	hypothetical protein
WP_071277339.1	hypothetical protein
WP_071277342.1	hypothetical protein
WP_071277343.1	hypothetical protein
WP_071277347.1	glucose-1-phosphate thymidylyltransferase
WP_071277349.1	hypothetical protein
WP_071277350.1	hypothetical protein
WP_071277351.1	hypothetical protein
WP_071277353.1	hypothetical protein

WP_071277368.1	hypothetical protein
WP_071277372.1	UDP-glucose 4-epimerase
WP_071277373.1	glycosyl transferase
WP_071277374.1	hypothetical protein
WP_071277377.1	hypothetical protein
WP_071277378.1	hypothetical protein
WP_071277382.1	hypothetical protein
WP_071277383.1	hypothetical protein
WP_071277384.1	hypothetical protein
WP_071277385.1	hypothetical protein
WP_071277386.1	hypothetical protein
WP_071277387.1	hypothetical protein
WP_071277389.1	peptidase S8
WP_071277391.1	hypothetical protein
WP_071277395.1	hypothetical protein
WP_071277399.1	hypothetical protein
WP_071277400.1	hypothetical protein
WP_071277401.1	hypothetical protein
WP_071277406.1	hypothetical protein
WP_071277407.1	hypothetical protein
WP_071277408.1	hypothetical protein
WP_071277409.1	2,4-diacetylphloroglucinol biosynthesis protein
WP_071277410.1	hypothetical protein
WP_071277411.1	hypothetical protein
WP_071277412.1	hypothetical protein
WP_071277413.1	hypothetical protein
WP_071277414.1	hypothetical protein
WP_071277415.1	hypothetical protein
WP_071277421.1	hypothetical protein

WP_071277423.1	hypothetical protein
WP_071277431.1	hypothetical protein
WP_071277437.1	TetR family transcriptional regulator
WP_071277440.1	hypothetical protein
WP_071277444.1	hypothetical protein
WP_071277446.1	hypothetical protein
WP_071277448.1	hypothetical protein
WP_071277450.1	hypothetical protein
WP_071277454.1	hypothetical protein
WP_071277456.1	recombinase
WP_071277458.1	hypothetical protein
WP_071277462.1	hypothetical protein
WP_071277464.1	hypothetical protein
WP_071277466.1	hypothetical protein
WP_071277468.1	hypothetical protein
WP_071277470.1	hypothetical protein
WP_071277472.1	hypothetical protein
WP_071277478.1	hypothetical protein

Table S6. COG category wise classification of 84 unique proteins of *B.aryabhattai* AB211*

COGs	Description	Number (Category)	%	Category
J	Translation, ribosomal structure and biogenesis	1	1.05	Information storage and processing
K	Transcription	8	8.42	
L	Replication, recombination and repair	2	2.11	
D	Cell cycle control, cell division, chromosome partitioning	0	0.00	Cellular processes and signaling
V	Defense mechanisms	4	4.21	
T	Signal transduction mechanisms	4	4.21	
M	Cell wall/membrane/envelope biogenesis	18	18.95	
N	Cell motility	0	0.00	
U	Intracellular trafficking, secretion, and vesicular transport	2	2.11	
O	Posttranslational modification, protein turnover, chaperones	1	1.05	
C	Energy production and conversion	2	2.11	Metabolism
G	Carbohydrate transport and metabolism	13	13.68	
E	Amino acid transport and metabolism	7	7.37	
F	Nucleotide transport and metabolism	1	1.05	
H	Coenzyme transport and metabolism	1	1.05	
I	Lipid transport and metabolism	4	4.21	
P	Inorganic ion transport and metabolism	2	2.11	
Q	Secondary metabolites biosynthesis, transport and catabolism	3	3.16	
R	General function prediction only	11	11.58	Poorly characterized
S	Function unknown	11	11.58	

* Although total COG Number is 84 but total COG category number is 95, as multi-category COGs are counted multiple times.

Table S7. Motility and signaling genes in *B.aryabhattai* AB211

No	Contig_id/Size/start-end	Subcategory	Subsystem	Function (EC No.)
1	scaffold11|size4199117_700953_701954	Motility and Chemotaxis - no subcategory	Bacterial Chemotaxis	Flagellar motor switch protein FliM
2	scaffold11|size4199117_3478388_3477483	Motility and Chemotaxis - no subcategory	Bacterial Chemotaxis	Chemotaxis protein CheV (EC 2.7.3.-)
3	scaffold11|size4199117_714072_714500	Motility and Chemotaxis - no subcategory	Bacterial Chemotaxis	Positive regulator of CheA protein activity (CheW)
4	scaffold11|size4199117_701944_703074	Motility and Chemotaxis - no subcategory	Bacterial Chemotaxis	Flagellar motor switch protein FliN
5	scaffold11|size4199117_712062_714041	Motility and Chemotaxis - no subcategory	Bacterial Chemotaxis	Signal transduction histidine kinase CheA (EC 2.7.3.-)
6	scaffold11|size4199117_2312476_2311190	Motility and Chemotaxis - no subcategory	Bacterial Chemotaxis	Maltose/maltodextrin ABC transporter, substrate binding periplasmic protein MalE
7	scaffold11|size4199117_692182_693201	Motility and Chemotaxis - no subcategory	Bacterial Chemotaxis	Flagellar motor switch protein FliG
8	scaffold11|size4199117_711004_712047	Motility and Chemotaxis - no subcategory	Bacterial Chemotaxis	Chemotaxis response regulator protein-glutamate methylesteraseCheB (EC 3.1.1.61)
9	scaffold11|size4199117_554970_555752; scaffold11|size4199117_2425822_2429382	Motility and Chemotaxis - no subcategory	Bacterial Chemotaxis	Chemotaxis protein methyltransferaseCheR (EC 2.1.1.80)
10	scaffold11|size4199117_714551_715045	Motility and Chemotaxis - no subcategory	Bacterial Chemotaxis	Chemotaxis protein CheD
11	scaffold11|size4199117_703096_703458	Motility and Chemotaxis - no subcategory	Bacterial Chemotaxis	Chemotaxis regulator - transmits chemoreceptor signals to flagelllar motor components CheY
12	scaffold11|size4199117_689776_690225	Flagellar motility in Prokaryota	Flagellum in Campylobacter	Flagellar basal-body rod protein FlgC
13	scaffold11|size4199117_3701896_3702624	Flagellar motility in Prokaryota	Flagellum in Campylobacter	Flagellin protein FlaA
14	Scaffold8|size138119_10229_12400	Flagellar motility in Prokaryota	Flagellum in Campylobacter	Cell division protein FtsI [Peptidoglycan synthetase] (EC 2.4.1.129)
15	scaffold11|size4199117_690238_690528	Flagellar motility in Prokaryota	Flagellum in Campylobacter	Flagellar hook-basal body complex protein FliE

16	scaffold11|size4199117_689384_689773	Flagellar motility in Prokaryota	Flagellum in Campylobacter	Flagellar basal-body rod protein FlgB
17	scaffold11|size4199117_710125_710994	Flagellar motility in Prokaryota	Flagellum	Flagellar synthesis regulator FleN
18	scaffold11|size4199117_360549_361682	Flagellar motility in Prokaryota	Flagellum	RNA polymerase sigma factor RpoD
19	scaffold11|size4199117_700501_700932	Flagellar motility in Prokaryota	Flagellum	Flagellar biosynthesis protein FliL
20	Scaffold10|size375231_171775_169955	Flagellar motility in Prokaryota	Flagellum	Flagellar hook-associated protein FliD
21	Scaffold10|size375231_183747_182236	Flagellar motility in Prokaryota	Flagellum	Flagellar hook-associated protein FlgK
22	scaffold11|size4199117_693194_693982	Flagellar motility in Prokaryota	Flagellum	Flagellar assembly protein FliH
23	scaffold11|size4199117_705227_705883	Flagellar motility in Prokaryota	Flagellum	Flagellar biosynthesis protein FliR
24	scaffold11|size4199117_704156_704824	Flagellar motility in Prokaryota	Flagellum	Flagellar biosynthesis protein FliP
25	scaffold11|size4199117_699458_700252	Flagellar motility in Prokaryota	Flagellum	Flagellar hook protein FlgE
26	scaffold11|size4199117_690578_692170	Flagellar motility in Prokaryota	Flagellum	Flagellar M-ring protein FliF
27	scaffold11|size4199117_699004_699432	Flagellar motility in Prokaryota	Flagellum	Flagellar basal-body rod modification protein FlgD
28	scaffold11|size4199117_689776_690225	Flagellar motility in Prokaryota	Flagellum	Flagellar basal-body rod protein FlgC
29	Scaffold10|size375231_184503_184249	Flagellar motility in Prokaryota	Flagellum	Negative regulator of flagellin synthesis
30	scaffold11|size4199117_705886_706968	Flagellar motility in Prokaryota	Flagellum	Flagellar biosynthesis protein FlhB
31	scaffold11|size4199117_690238_690528	Flagellar motility in Prokaryota	Flagellum	Flagellar hook-basal body complex protein FliE
32	scaffold11|size4199117_689384_689773	Flagellar motility in Prokaryota	Flagellum	Flagellar basal-body rod protein FlgB
33	scaffold11|size4199117_703522_704163	Flagellar motility in Prokaryota	Flagellum	Flagellar biosynthesis protein FliZ
34	Scaffold10|size375231_169938_169537;	Flagellar motility in Prokaryota	Flagellum	Flagellar biosynthesis protein FliS

	Scaffold10|size375231_189359_188925			
35	Scaffold10|size375231_211288_210464	Flagellar motility in Prokaryota	Flagellum	Flagellar basal-body rod protein FlgF
36	scaffold11|size4199117_3701896_3702624	Flagellar motility in Prokaryota	Flagellum	Flagellin protein FlaA
37	scaffold11|size4199117_701944_703074	Flagellar motility in Prokaryota	Flagellum	Flagellar motor switch protein FliN
38	scaffold11|size4199117_695726_696358	Flagellar motility in Prokaryota	Flagellum	Flagellar protein FlbB
39	Scaffold10|size375231_210440_209619	Flagellar motility in Prokaryota	Flagellum	Flagellar basal-body rod protein FlgG
40	Scaffold10|size375231_124523_123213; scaffold11|size4199117_3908800_3910134	Flagellar motility in Prokaryota	Flagellum	RNA polymerase sigma-54 factor RpoN
41	scaffold11|size4199117_84890_85501; scaffold11|size4199117_2710660_2709827	Flagellar motility in Prokaryota	Flagellum	Flagellar motor rotation protein MotB
42	scaffold11|size4199117_695303_695716	Flagellar motility in Prokaryota	Flagellum	Flagellar protein FliJ
43	Scaffold10|size375231_182233_181352	Flagellar motility in Prokaryota	Flagellum	Flagellar hook-associated protein FlgL
44	scaffold11|size4199117_706999_709035	Flagellar motility in Prokaryota	Flagellum	Flagellar biosynthesis protein FlhA
45	scaffold11|size4199117_693957_695270	Flagellar motility in Prokaryota	Flagellum	Flagellum-specific ATP synthase FliI
46	scaffold11|size4199117_704835_705104	Flagellar motility in Prokaryota	Flagellum	Flagellar biosynthesis protein FliQ
47	scaffold11|size4199117_696376_698994; scaffold11|size4199117_3611015_3609321	Flagellar motility in Prokaryota	Flagellum	Flagellar hook-length control protein FliK
48	scaffold11|size4199117_700953_701954	Flagellar motility in Prokaryota	Flagellum	Flagellar motor switch protein FliM
49	scaffold11|size4199117_709035_710132	Flagellar motility in Prokaryota	Flagellum	Flagellar biosynthesis protein FlhF

50	scaffold11|size4199117_84052_84855; scaffold11|size4199117_2711447_2710653	Flagellar motility in Prokaryota	Flagellum	Flagellar motor rotation protein MotA
51	scaffold11|size4199117_692182_693201	Flagellar motility in Prokaryota	Flagellum	Flagellar motor switch protein FliG
52	scaffold11|size4199117_700288_700497	Flagellar motility in Prokaryota	Flagellum	Flagellar protein FlbD
53	scaffold11|size4199117_715102_715863	Flagellar motility in Prokaryota	Flagellum	RNA polymerase sigma factor for flagellar operon
54	Scaffold8|size138119_70287_69856; scaffold11|size4199117_1568244_1567765	Flagellar motility in Prokaryota	Flagellum	Rrf2 family transcriptional regulator

Table S8. Stress response genes in *B.aryabhattai* AB211

No.	Contig_id/Size/start-end	Subcategory	Subsystem	Function (EC No.)
1	Scaffold8|size138119_79808_78981	Osmotic stress	Osmoregulation	Glycerol uptake facilitator protein
2	scaffold11|size4199117_3246007_3245126	Osmotic stress	Choline and Betaine Uptake and Betaine Biosynthesis	Glycine betaine ABC transport system, permease/glycine betaine-binding protein OpuABC
3	scaffold11|size4199117_3256625_3256939; scaffold11|size4199117_3257042_3257185; scaffold12|size137365_43435_43995	Osmotic stress	Choline and Betaine Uptake and Betaine Biosynthesis	Choline binding protein A
4	Scaffold10|size375231_178568_179770	Osmotic stress	Choline and Betaine Uptake and Betaine Biosynthesis	Alcohol dehydrogenase GbsB (type III), essential for the utilization of choline (EC 1.1.1.1)
5	scaffold11|size4199117_959557_960507; scaffold11|size4199117_3248148_3246895	Osmotic stress	Choline and Betaine Uptake and Betaine Biosynthesis	Glycine betaine ABC transport system, ATP-binding protein OpuAA (EC 3.6.3.32)
6	scaffold11|size4199117_2324781_2323252	Osmotic stress	Choline and Betaine Uptake and Betaine Biosynthesis	Choline-sulfatase (EC 3.1.6.6)
7	Scaffold10|size375231_177062_178546; scaffold11|size4199117_3943342_3941855	Osmotic stress	Choline and Betaine Uptake and Betaine Biosynthesis	Betaine aldehyde dehydrogenase (EC 1.2.1.8)
8	scaffold11|size4199117_3442398_3443918	Osmotic stress	Choline and Betaine Uptake and Betaine Biosynthesis	Glycine betaine transporter OpuD
9	scaffold11|size4199117_960507_962030	Osmotic stress	Choline and Betaine Uptake and Betaine Biosynthesis	L-proline glycine betaine binding ABC transporter protein ProX (TC 3.A.1.12.1)
10	scaffold11|size4199117_3944308_3944748	Osmotic stress	Choline and Betaine Uptake and Betaine Biosynthesis	Glycine betaine ABC transport system, permease protein OpuAB
11	scaffold11|size4199117_3246892_3246029	Osmotic stress	Choline and Betaine Uptake and Betaine Biosynthesis	L-proline glycine betaine ABC transport system permease protein ProW (TC 3.A.1.12.1)
12	scaffold11|size4199117_379142_379750	Oxidative stress	Protection from Reactive Oxygen Species	Manganese superoxide dismutase (EC 1.15.1.1)

13	Scaffold10|size375231_26915_26391; scaffold11|size4199117_2595477_2594887	Oxidative stress	Protection from Reactive Oxygen Species	Superoxide dismutase [Cu-Zn] precursor (EC 1.15.1.1)
14	scaffold11|size4199117_2002990_2003874	Oxidative stress	Protection from Reactive Oxygen Species	Superoxide dismutase [Fe] (EC 1.15.1.1)
15	Scaffold10|size375231_317129_318589; scaffold11|size4199117_949496_947454; scaffold11|size4199117_1723636_1721942	Oxidative stress	Protection from Reactive Oxygen Species	Catalase (EC 1.11.1.6)
16	scaffold11|size4199117_30945_31385; scaffold11|size4199117_1748746_1748294	Oxidative stress	Oxidative stress	Iron-binding ferritin-like antioxidant protein
17	scaffold11|size4199117_2436893_2437342	Oxidative stress	Oxidative stress	Organic hydroperoxide resistance transcriptional regulator
18	scaffold11|size4199117_3461814_3461266; scaffold11|size4199117_3814712_3814170	Oxidative stress	Oxidative stress	Alkyl hydroperoxidereductase subunit C-like protein
19	scaffold11|size4199117_372124_372537	Oxidative stress	Oxidative stress	Zinc uptake regulation protein ZUR
20	scaffold11|size4199117_30945_31385; scaffold11|size4199117_1748746_1748294	Oxidative stress	Oxidative stress	Non-specific DNA-binding protein Dps
21	Scaffold3|size19195_5953_6591	Oxidative stress	Oxidative stress	Redox-sensitive transcriptional regulator (AT-rich DNA-binding protein)
22	Scaffold9|size151310_2307_1873	Oxidative stress	Oxidative stress	Peroxide stress regulator PerR, FUR family
23	scaffold11|size4199117_491299_491751	Oxidative stress	Oxidative stress	Ferric uptake regulation protein FUR
24	Scaffold9|size151310_51017_51466	Oxidative stress	Oxidative stress	Nitrite-sensitive transcriptional repressor NsrR
25	scaffold11|size4199117_4146135_4146833	Oxidative stress	Oxidative stress	transcriptional regulator, Crp/Fnr family
26	Scaffold9|size151310_94119_93700; scaffold11|size4199117_2437824_2437405	Oxidative stress	Oxidative stress	Organic hydroperoxide resistance protein
27	scaffold11|size4199117_3033077_3032652	Oxidative stress	Glutathione: Non-redox reactions	Lactoylglutathionelyase (EC 4.4.1.5)
28	scaffold11|size4199117_397164_397796	Oxidative stress	Glutathione: Non-redox reactions	Similar to Hydroxyacylglutathione hydrolase, but in an organism lacking glutathione biosynthesis

29	scaffold11|size4199117_480806_481762	Oxidative stress	Glutathione: Non-redox reactions	Hydroxyacylglutathione hydrolase (EC 3.1.2.6)
30	scaffold11|size4199117_2530326_2528857; scaffold11|size4199117_3556461_3554941	Oxidative stress	Redox-dependent regulation of nucleus processes	Nicotinatephosphoribosyltransferase (EC 2.4.2.11)
31	scaffold11|size4199117_145946_146977	Oxidative stress	Redox-dependent regulation of nucleus processes	NADPH-dependent glyceraldehyde-3-phosphate dehydrogenase (EC 1.2.1.13)
32	Scaffold2|size15766_4636_5178; scaffold11|size4199117_2530898_2530350	Oxidative stress	Redox-dependent regulation of nucleus processes	Nicotinamidase (EC 3.5.1.19)
33	scaffold11|size4199117_900664_901383	Oxidative stress	Redox-dependent regulation of nucleus processes	NAD-dependent protein deacetylase of SIR2 family
34	Scaffold10|size375231_121743_120736	Oxidative stress	Redox-dependent regulation of nucleus processes	NAD-dependent glyceraldehyde-3-phosphate dehydrogenase (EC 1.2.1.12)
35	Scaffold8|size138119_57254_57874; Scaffold9|size151310_50800_49619; scaffold11|size4199117_2920628_2919408	Oxidative stress	Glutaredoxins	Flavohemoprotein (Hemoglobin-like protein) (Flavohemoglobin) (Nitric oxide dioxygenase) (EC 1.14.12.17)
36	Scaffold10|size375231_53739_54740	Oxidative stress	Glutaredoxins	Cell wall endopeptidase, family M23/M37
37	scaffold11|size4199117_1716714_1717973	Oxidative stress	Glutathionylspermidine and Trypanothione	Similarity with glutathionylspermidine synthase (EC 6.3.1.8), group 2
38	scaffold11|size4199117_1593488_1593685; scaffold11|size4199117_3335212_3335415; scaffold11|size4199117_3376983_3376780; scaffold11|size4199117_3817415_3817612	Cold shock	Cold shock, CspA family of proteins	Cold shock protein CspD
39	scaffold11|size4199117_1061505_1061705; scaffold11|size4199117_1236763_1236963; scaffold11|size4199117_1433933_1434136; scaffold11|size4199117_1435007_1434807; scaffold11|size4199117_2014664_2014464; scaffold11|size4199117_2019057_2018857; scaffold11|size4199117_3078844_3079044; scaffold11|size4199117_3081492_3081289	Cold shock	Cold shock, CspA family of proteins	Cold shock protein CspA
40	scaffold11|size4199117_1360036_1359824	Cold shock	Cold shock, CspA family of proteins	Cold shock protein CspE
41	scaffold11|size4199117_334939_336078	Heat shock	Heat shock dnaK gene cluster	Hypothetical radical SAM

			extended	family enzyme in heat shock gene cluster, similarity with CPO of BS HemN-type
42	scaffold11|size4199117_336170_337198	Heat shock	Heat shock dnaK gene cluster extended	Heat-inducible transcription repressor HrcA
43	scaffold11|size4199117_337895_339712	Heat shock	Heat shock dnaK gene cluster extended	Chaperone protein DnaK
44	scaffold11|size4199117_342915_344273	Heat shock	Heat shock dnaK gene cluster extended	tRNA-t(6)A37 methylthiotransferase
45	scaffold11|size4199117_339948_341078	Heat shock	Heat shock dnaK gene cluster extended	Chaperone protein DnaJ
46	scaffold11|size4199117_342157_342909	Heat shock	Heat shock dnaK gene cluster extended	Ribosomal RNA small subunit methyltransferase E (EC 2.1.1.-)
47	Scaffold10|size375231_111476_111009	Heat shock	Heat shock dnaK gene cluster extended	tmRNA-binding protein SmpB
48	scaffold11|size4199117_337304_337864	Heat shock	Heat shock dnaK gene cluster extended	Heat shock protein GrpE
49	Scaffold6|size49359_44506_44141	Heat shock	Heat shock dnaK gene cluster extended	DNA replication intiation control protein YabA
50	scaffold11|size4199117_332940_334763	Heat shock	Heat shock dnaK gene cluster extended	Translation elongation factor LepA
51	Scaffold6|size49359_19973_19707	Heat shock	Heat shock dnaK gene cluster extended	Ribosome-associated heat shock protein implicated in the recycling of the 50S subunit (S4 paralog)
52	scaffold11|size4199117_202305_202898	Heat shock	Heat shock dnaK gene cluster extended	Nucleoside 5-triphosphatase RdgB (dHAPTP, dITP, XTP-specific) (EC 3.6.1.15)
53	scaffold11|size4199117_341196_342137	Heat shock	Heat shock dnaK gene cluster extended	Ribosomal protein L11 methyltransferase (EC 2.1.1.-)
54	Scaffold6|size49359_45348_44521	Heat shock	Heat shock dnaK gene cluster extended	Signal peptidase-like protein
55	scaffold11|size4199117_202914_203426	Heat shock	Heat shock dnaK gene cluster extended	FIG009886: phosphoesterase
56	scaffold11|size4199117_201549_202295	Heat shock	Heat shock dnaK gene cluster extended	Ribonuclease PH (EC 2.7.7.56)
57	Scaffold6|size49359_43064_42192	Heat shock	Heat shock dnaK gene cluster	rRNA small subunit

			extended	methyltransferase I
58	scaffold11|size4199117_2606026_2604953	Detoxification	Uptake of selenate and selenite	Sulfate and thiosulfate import ATP-binding protein CysA (EC 3.6.3.25)
59	Scaffold10|size375231_79735_78686	Stress Response - no subcategory	Flavohaemoglobin	ABC-type Fe3+-siderophore transport system, permease 2 component
60	Scaffold5|size51851_8309_7953	Stress Response - no subcategory	SigmaB stress responce regulation	RsbS, negative regulator of sigma-B
61	Scaffold5|size51851_6135_5650	Stress Response - no subcategory	SigmaB stress responce regulation	Serine-protein kinase RsbW (EC 2.7.11.1)
62	Scaffold5|size51851_7534_6524; scaffold11|size4199117_3238648_3239790	Stress Response - no subcategory	SigmaB stress responce regulation	Serine phosphatase RsbU, regulator of sigma subunit
63	Scaffold5|size51851_9148_8312; Scaffold9|size151310_78883_78131	Stress Response - no subcategory	SigmaB stress responce regulation	RsbR, positive regulator of sigma-B
64	Scaffold5|size51851_5684_4890	Stress Response - no subcategory	SigmaB stress responce regulation	RNA polymerase sigma factor SigB
65	Scaffold5|size51851_6464_6132; scaffold11|size4199117_496685_497035	Stress Response - no subcategory	SigmaB stress responce regulation	Anti-sigma B factor antagonist RsbV
66	scaffold11|size4199117_4160054_4158849	Stress Response - no subcategory	Dimethylarginine metabolism	Ornithine aminotransferase (EC 2.6.1.13)
67	scaffold11|size4199117_2416620_2415760	Stress Response - no subcategory	Dimethylarginine metabolism	NG,NG-dimethylargininedimethylaminohydrolase 1 (EC 3.5.3.18)
68	scaffold11|size4199117_1047708_1049423; scaffold11|size4199117_1355489_1357138; scaffold11|size4199117_2365420_2363696; scaffold11|size4199117_3316388_3313620; scaffold11|size4199117_3709491_3707041; scaffold11|size4199117_3714227_3711696; scaffold11|size4199117_3753056_3751083	Stress Response - no subcategory	Bacterial hemoglobins	diguanylatecyclase/phosphodiesterase (GGDEF & EAL domains) with PAS/PAC sensor(s)
69	scaffold11|size4199117_2301076_2300714	Stress Response - no subcategory	Bacterial hemoglobins	Cyanoglobin
70	scaffold11|size4199117_4089899_4090309	Stress Response - no subcategory	Bacterial hemoglobins	Hemoglobin-like protein HbO
71	scaffold11|size4199117_788390_789652	Stress Response - no subcategory	Hfl operon	GTP-binding protein HflX

72	scaffold11|size4199117_786100_786324	Stress Response - no subcategory	Hfl operon	RNA-binding protein Hfq
73	Scaffold10|size375231_180824_180594	Stress Response - no subcategory	Carbon Starvation	Carbon storage regulator
74	Scaffold10|size375231_315046_316854	Stress Response - no subcategory	Carbon Starvation	Carbon starvation protein A

Table S9. Nitrogen metabolism genes in *B.aryabhattai* AB211

No.	Contig_id/Size/start-end	Subcategory	Subsystem	Function (EC No.)
1	scaffold11|size4199117_379142_379750	Nitrogen Metabolism - no subcategory	Nitric oxide synthase	Manganese superoxide dismutase (EC 1.15.1.1)
2	scaffold11|size4199117_1003897_1005111; scaffold11|size4199117_2672748_2671537; scaffold11|size4199117_2887694_2888926	Nitrogen Metabolism - no subcategory	Nitric oxide synthase	putative cytochrome P450 hydroxylase
3	Scaffold9|size151310_52904_51819	Nitrogen Metabolism - no subcategory	Nitric oxide synthase	Nitric oxide synthase oxygenase (EC 1.-.-.-)
4	Scaffold9|size151310_51017_51466	Nitrogen Metabolism - no subcategory	Nitrosative stress	Nitrite-sensitive transcriptional repressor NsrR
5	scaffold11|size4199117_4036943_4034793	Nitrogen Metabolism - no subcategory	Nitrate and nitrite ammonification	Assimilatory nitrate reductase large subunit (EC:1.7.99.4)
6	scaffold11|size4199117_3144449_3143118; scaffold11|size4199117_4033563_4034744	Nitrogen Metabolism - no subcategory	Nitrate and nitrite ammonification	Nitrate/nitrite transporter
7	Scaffold5|size51851_19236_19748	Nitrogen Metabolism - no subcategory	Nitrate and nitrite ammonification	PolyferredoxinNapH (periplasmic nitrate reductase)
8	scaffold11|size4199117_2215840_2216958; scaffold11|size4199117_3271795_3270575	Nitrogen Metabolism - no subcategory	Nitrate and nitrite ammonification	Nitrate/nitrite sensor protein (EC 2.7.3.-)
9	scaffold11|size4199117_3637966_3637640	Nitrogen Metabolism - no subcategory	Nitrate and nitrite ammonification	Nitrite reductase [NAD(P)H] small subunit (EC 1.7.1.4)
10	scaffold11|size4199117_3640399_3637985; scaffold11|size4199117_4039308_4036963	Nitrogen Metabolism - no subcategory	Nitrate and nitrite ammonification	Nitrite reductase [NAD(P)H] large subunit (EC 1.7.1.4)
11	scaffold11|size4199117_791603_792937	Nitrogen Metabolism - no subcategory	Ammonia assimilation	Glutamine synthetase type I (EC 6.3.1.2)
12	scaffold11|size4199117_2629942_2628461	Nitrogen Metabolism - no subcategory	Ammonia assimilation	Glutamate synthase [NADPH] small chain (EC 1.4.1.13)
13	scaffold11|size4199117_3649858_3648278	Nitrogen Metabolism - no subcategory	Ammonia assimilation	Ferredoxin-dependent glutamate synthase (EC 1.4.7.1)

14	Scaffold6|size49359_46708_46379; scaffold11|size4199117_1815523_1815885; scaffold11|size4199117_4166551_4166210	Nitrogen Metabolism - no subcategory	Ammonia assimilation	Nitrogen regulatory protein P-II
15	scaffold11|size4199117_3171103_3169058	Nitrogen Metabolism - no subcategory	Ammonia assimilation	Nitrogen regulation protein NR(I)
16	scaffold11|size4199117_1814282_1815508; scaffold11|size4199117_2101215_2102498; scaffold11|size4199117_4166551_4166210	Nitrogen Metabolism - no subcategory	Ammonia assimilation	Ammonium transporter
17	scaffold11|size4199117_2634512_2629959; scaffold11|size4199117_3590446_3591243	Nitrogen Metabolism - no subcategory	Ammonia assimilation	Glutamate synthase [NADPH] large chain (EC 1.4.1.13)
18	scaffold11|size4199117_2566218_2564302	Denitrification	Denitrifying reductase gene clusters	Nitric oxide reductase activation protein NorD
19	scaffold11|size4199117_2567120_2566230	Denitrification	Denitrifying reductase gene clusters	Nitric oxide reductase activation protein NorQ

Table S10. Siderophores and iron acquisition genes in *B.aryabhattai* AB211

No.	Contig_id/Size/start-end	Subcategory	Subsystem	Function (EC No.)
1	scaffold11|size4199117_828645_830399	Siderophores	Siderophore assembly kit	Siderophoresynthetase large component, acetyltransferase
2	scaffold11|size4199117_825781_827145; scaffold11|size4199117_3726269_3724953; scaffold11|size4199117_3865838_3864519	Siderophores	Siderophore assembly kit	Siderophore biosynthesis diaminobutyrate--2-oxoglutarate aminotransferase (EC 2.6.1.76)
3	scaffold11|size4199117_827147_828667; scaffold11|size4199117_2921055_2922182	Siderophores	Siderophore assembly kit	Siderophore biosynthesis L-2,4-diaminobutyrate decarboxylase
4	scaffold11|size4199117_833412_835217	Siderophores	Siderophore assembly kit	Siderophoresynthetase component, ligase
5	Scaffold10|size375231_80916_81902	Siderophores	Siderophore assembly kit	ABC-type Fe3+-siderophore transport system, periplasmic iron-binding component
6	scaffold11|size4199117_3334345_3333104	Siderophores	Siderophore assembly kit	Siderophore transport protein
7	scaffold14|size51411_36790_39498	Siderophores	Siderophore assembly kit	Siderophore biosynthesis non-ribosomal peptide synthetase modules
8	Scaffold10|size375231_78659_77832; scaffold11|size4199117_3308550_3307765	Siderophores	Siderophore assembly kit	ABC-type Fe3+-siderophore transport system, ATPase component
9	Scaffold10|size375231_79735_78686	Siderophores	Siderophore assembly kit	ABC-type Fe3+-siderophore transport

				system, permease 2 component
10	scaffold11|size4199117_830396_830989	Siderophores	Siderophore assembly kit	Siderophoresynthetase small component, acetyltransferase
11	Scaffold10|size375231_80736_79732; scaffold11|size4199117_3278700_3279710; scaffold11|size4199117_3935726_3934692;	Siderophores	Siderophore assembly kit	ABC-type Fe3+-siderophore transport system, permease component
12	scaffold11|size4199117_830968_832257	Siderophores	Siderophore assembly kit	Siderophore biosynthesis protein, monooxygenase
13	scaffold11|size4199117_832261_833415	Siderophores	Siderophore assembly kit	Siderophore related permease
14	scaffold11|size4199117_1584854_1585849	Siderophores	SiderophoreAnthrachelin	Iron compound ABC uptake transporter substrate-binding protein
15	scaffold11|size4199117_1584114_1584872	Siderophores	SiderophoreAnthrachelin	Uncharacterized iron compound ABC uptake transporter, ATP-binding protein
16	scaffold11|size4199117_1582230_1583180; scaffold11|size4199117_1583173_1584120	Siderophores	SiderophoreAnthrachelin	Iron compound ABC uptake transporter permease protein
17	scaffold11|size4199117_1530119_1530793	Iron acquisition and metabolism - no subcategory	Heme, hemin uptake and utilization systems in GramPositives	Two-component response regulator colocalized with HrtAB transporter
18	scaffold11|size4199117_3310555_3309548	Iron acquisition and metabolism - no subcategory	Heme, hemin uptake and utilization systems in GramPositives	Heme ABC type transporter HtsABC, permease protein HtsB
19	scaffold11|size4199117_3311596_3310646; scaffold11|size4199117_3936750_3935806	Iron acquisition and metabolism - no subcategory	Heme, hemin uptake and utilization systems in GramPositives	Heme ABC type transporter HtsABC, heme-binding protein
20	Scaffold10|size375231_94155_92800; Scaffold10|size375231_342545_340638	Iron acquisition and metabolism - no subcategory	Heme, hemin uptake and utilization systems in	Two-component sensor kinase SA14-24

			GramPositives	
21	scaffold11|size4199117_1530790_1532172; scaffold11|size4199117_2611555_2610176	Iron acquisition and metabolism - no subcategory	Heme, hemin uptake and utilization systems in GramPositives	Sensor histidine kinase colocalized with HrtAB transporter
22	scaffold11|size4199117_3309551_3308571; scaffold11|size4199117_3934685_3933669	Iron acquisition and metabolism - no subcategory	Heme, hemin uptake and utilization systems in GramPositives	Heme ABC type transporter HtsABC, permease protein HtsC
23	Scaffold10|size375231_343259_342552	Iron acquisition and metabolism - no subcategory	Heme, hemin uptake and utilization systems in GramPositives	Two-component response regulator SA14-24
24	scaffold11|size4199117_1533330_1534025	Iron acquisition and metabolism - no subcategory	Heme, hemin uptake and utilization systems in GramPositives	Heme efflux system ATPase HrtA
25	scaffold11|size4199117_1532245_1533330	Iron acquisition and metabolism - no subcategory	Heme, hemin uptake and utilization systems in GramPositives	Heme efflux system permeaseHrtB
26	Scaffold10|size375231_92053_92766	Iron acquisition and metabolism - no subcategory	Ferrous iron transporter EfeUOB, low-pH-induced	Ferrous iron transport permeaseEfeU
27	scaffold11|size4199117_175181_175954	Iron acquisition and metabolism - no subcategory	Heme, hemin uptake and utilization systems in GramNegatives	Electron transfer flavoprotein, beta subunit
28	scaffold11|size4199117_1590111_1589497	Iron acquisition and metabolism - no subcategory	Heme, hemin uptake and utilization systems in GramNegatives	Flavinreductase (EC 1.5.1.30)
29	scaffold11|size4199117_3276900_3277694	Iron acquisition and metabolism - no subcategory	Heme, hemin uptake and utilization systems in GramNegatives	Ferrichrome transport ATP-binding protein FhuC (TC 3.A.1.14.3)

Table S11. Antibiotic and heavy metal resistance genes in *Bacillus aryabhattai* AB211

No	Contig_id/Size/start-end	Subcategory	Subsystem	Function (EC No.)
1	scaffold11|size4199117_1014685_1 15356; scaffold11|size4199117_1497004_1496312	Bacteriocins, ribosomally synthesized antibacterial peptides	Bacitracin Stress Response	Two-component response regulator BceR
2	scaffold11|size4199117_3979521_3980390	Bacteriocins, ribosomally synthesized antibacterial peptides	Bacitracin Stress Response	Conserved protein LiaG in B. subtilis in Lia cluster
3	scaffold11|size4199117_3364384_3363758	Bacteriocins, ribosomally synthesized antibacterial peptides	Bacitracin Stress Response	Response regulator LiaR
4	scaffold11|size4199117_1016833_1017600; scaffold11|size4199117_1495214_1494453	Bacteriocins, ribosomally synthesized antibacterial peptides	Bacitracin Stress Response	Bacitracin export ATP-binding protein BceA
5	scaffold11|size4199117_1494463_1492517	Bacteriocins, ribosomally synthesized antibacterial peptides	Bacitracin Stress Response	Bacitracin export permease protein BceB
6	scaffold11|size4199117_1015358_1016392; scaffold11|size4199117_1496319_1495315	Bacteriocins, ribosomally synthesized antibacterial peptides	Bacitracin Stress Response	Two-component sensor histidine kinase BceS
7	Scaffold9|size151310_39899_41818; scaffold11|size4199117_1625736_1623178; scaffold11|size4199117_2806918_2804501; scaffold11|size4199117_2964713_2962596; scaffold12|size137365_55382_57805	Resistance to antibiotics and toxic compounds	Copper homeostasis	Copper-translocating P-type ATPase (EC 3.6.3.4)
8	scaffold11|size4199117_1751353_1751913	Resistance to antibiotics and toxic compounds	Copper homeostasis	Copper resistance protein CopC
9	Scaffold9|size151310_107510_106290	Resistance to antibiotics and toxic compounds	Copper homeostasis	Multidrug resistance transporter, Bcr/CflA family
10	scaffold11|size4199117_2973022_2971418	Resistance to antibiotics and toxic compounds	Copper homeostasis	Copper resistance protein D
11	Scaffold4|size36871_26265_25369; Scaffold8|size138119_89273_88398; Scaffold9|size151310_38982_38056; scaffold11|size4199117_3007851_3007714	Resistance to antibiotics and toxic compounds	Cobalt-zinc-cadmium resistance	Cobalt-zinc-cadmium resistance protein
12	Scaffold9|size151310_38982_38056; scaffold11|size4199117_3007851_3007714	Resistance to antibiotics and toxic compounds	Cobalt-zinc-cadmium resistance	Cobalt-zinc-cadmium resistance protein CzcD
13	scaffold11|size4199117_2661165_2660731	Resistance to antibiotics and toxic compounds	Cobalt-zinc-cadmium resistance	Heavy metal resistance transcriptional regulator HmrR

14	Scaffold9|size151310_108399_107578	Resistance to antibiotics and toxic compounds	Cobalt-zinc-cadmium resistance	Transcriptional regulator, MerR family
15	scaffold11|size4199117_2265586_2264624; scaffold11|size4199117_2469212_2470150	Resistance to antibiotics and toxic compounds	Resistance to Vancomycin	Vancomycin B-type resistance protein VanW
16	scaffold11|size4199117_2906442_2904379	Resistance to antibiotics and toxic compounds	Zinc resistance	Sensor protein of zinc sigma-54-dependent two-component system
17	scaffold11|size4199117_2845131_2843212; scaffold11|size4199117_3727713_3726343	Resistance to antibiotics and toxic compounds	Zinc resistance	Response regulator of zinc sigma-54-dependent two-component system
18	scaffold11|size4199117_2040497_2041129; scaffold11|size4199117_3284294_3283656; scaffold11|size4199117_3896528_3895890	Resistance to antibiotics and toxic compounds	Multidrug Resistance, 2-protein version Found in Gram-positive bacteria	Multidrug resistance protein [function not yet clear]
19	Scaffold8|size138119_108027_108539; scaffold11|size4199117_2005328_2006752; scaffold11|size4199117_3283622_3281997; scaffold11|size4199117_3895847_3894222	Resistance to antibiotics and toxic compounds	Multidrug Resistance, 2-protein version Found in Gram-positive bacteria	Membrane component of multidrug resistance system
20	scaffold11|size4199117_1165204_1165836; scaffold11|size4199117_1833801_1833193; scaffold11|size4199117_3004504_3003896	Resistance to antibiotics and toxic compounds	Multidrug Resistance, 2-protein version Found in Gram-positive bacteria	TetR family regulatory protein of MDR cluster
21	Scaffold7|size59084_29442_27364	Resistance to antibiotics and toxic compounds	Tetracycline resistance, ribosome protection type	Translation elongation factor G
22	scaffold11|size4199117_2253717_2255678	Resistance to antibiotics and toxic compounds	Tetracycline resistance, ribosome protection type	Ribosome protection-type tetracycline resistance related proteins, group 2
23	scaffold11|size4199117_1635934_1636476; scaffold11|size4199117_2556276_2556827	Resistance to antibiotics and toxic compounds	Aminoglycoside adenylyltransferases	Aminoglycoside N6'-acetyltransferase (EC 2.3.1.82)
24	Scaffold10|size375231_370381_372297	Resistance to antibiotics and toxic compounds	Resistance to fluoroquinolones	DNA gyrase subunit B (EC 5.99.1.3)
25	Scaffold10|size375231_372424_374934	Resistance to antibiotics and toxic compounds	Resistance to fluoroquinolones	DNA gyrase subunit A (EC 5.99.1.3)
26	scaffold11|size4199117_2173143_2175119	Resistance to antibiotics and toxic compounds	Resistance to fluoroquinolones	Topoisomerase IV subunit B (EC 5.99.1.-)
27	scaffold11|size4199117_2175116_2177542	Resistance to antibiotics and toxic compounds	Resistance to fluoroquinolones	Topoisomerase IV subunit A (EC 5.99.1.-)

28	scaffold11|size4199117_2795693_2795343;scaffold11|size4199117_3033457_3033128	Resistance to antibiotics and toxic compounds	Arsenic resistance	Arsenical resistance operon repressor
29	scaffold11|size4199117_2795326_2794028; scaffold11|size4199117_3034228_3035526; scaffold11|size4199117_3731144_3732499	Resistance to antibiotics and toxic compounds	Arsenic resistance	Arsenic efflux pump protein
30	Scaffold10|size375231_69815_69459; scaffold11|size4199117_3035551_3035973	Resistance to antibiotics and toxic compounds	Arsenic resistance	Arsenate reductase (EC 1.20.4.1)
31	scaffold11|size4199117_2540763_2540335	Resistance to antibiotics and toxic compounds	Fosfomycin resistance	Fosfomycin resistance protein FosB
32	scaffold11|size4199117_1655232_1656839	Resistance to antibiotics and toxic compounds	Multidrug Resistance Operon mdtRP of Bacillus	Multidrug efflux transporter MdtP
33	scaffold11|size4199117_1654757_1655209; scaffold11|size4199117_3281926_3281507; scaffold11|size4199117_3893079_3892654	Resistance to antibiotics and toxic compounds	Multidrug Resistance Operon mdtRP of Bacillus	MdtR transcriptional regulator, MarR family
34	Scaffold7|size59084_29442_27364	Resistance to antibiotics and toxic compounds	Tetracycline resistance, ribosome protection type, too	Translation elongation factor G
35	scaffold11|size4199117_2253717_2255678	Resistance to antibiotics and toxic compounds	Tetracycline resistance, ribosome protection type, too	Ribosome protection-type tetracycline resistance related proteins, group 2
36	scaffold11|size4199117_1471941_1472873	Resistance to antibiotics and toxic compounds	Beta-lactamase	Beta-lactamase class A
37	scaffold11|size4199117_3042191_3041163; scaffold11|size4199117_3318782_3318006	Resistance to antibiotics and toxic compounds	Beta-lactamase	Beta-lactamase (EC 3.5.2.6)
38	Scaffold8|size138119_4728_3997; scaffold11|size4199117_2305326_2304547	Resistance to antibiotics and toxic compounds	Beta-lactamase	Metal-dependent hydrolases of the beta-lactamase superfamily I
39	scaffold11|size4199117_1626127_1625768	Resistance to antibiotics and toxic compounds	Cadmium resistance	Cadmium efflux system accessory protein
40	scaffold11|size4199117_2041283_2042677; scaffold11|size4199117_2737257_2738630; scaffold11|size4199117_2933727_2932342	Resistance to antibiotics and toxic compounds	Multidrug Resistance Efflux Pumps	Multi antimicrobial extrusion protein (Na(+)/drug antiporter), MATE family of MDR efflux pumps
41	Scaffold10|size375231_85507_82397; scaffold11|size4199117_1641659_1644697; scaffold11|size4199117_3721682_3718653	Resistance to antibiotics and toxic compounds	Multidrug Resistance Efflux Pumps	Acriflavin resistance protein
42	scaffold11|size4199117_1026905_1025670;	Resistance to antibiotics and toxic	Multidrug Resistance Efflux	Multidrug and toxin

	scaffold11|size4199117_1595930_1595604	compounds	Pumps	extrusion (MATE) family efflux pump YdhE/NorM, homolog
43	scaffold11|size4199117_2137682_2136405	Resistance to antibiotics and toxic compounds	Multidrug Resistance Efflux Pumps	Multidrug-efflux transporter, major facilitator superfamily (MFS) (TC 2.A.1)

Table S12. Aromatic compound degradation pathway genes in *B.aryabhattai* AB211

No	Contig_id/Size/start-end	Subcategory	Subsystem	Function (EC No.)
1	scaffold11|size4199117_1835207_1836721	Peripheral pathways for catabolism of aromatic compounds	Salicylate ester degradation	Salicylate hydroxylase (EC 1.14.13.1)
2	scaffold11|size4199117_418568_419005	Peripheral pathways for catabolism of aromatic compounds	Quinate degradation	3-dehydroquinate dehydratase II (EC 4.2.1.10)
3	scaffold11|size4199117_3836324_3835428	Peripheral pathways for catabolism of aromatic compounds	Quinate degradation	Shikimate/quinate 5-dehydrogenase I beta (EC 1.1.1.282)
4	scaffold11|size4199117_3853469_3852684	Peripheral pathways for catabolism of aromatic compounds	Quinate degradation	3-dehydroquinate dehydratase I (EC 4.2.1.10)
5	scaffold11|size4199117_1301332_1300952	Peripheral pathways for catabolism of aromatic compounds	Biphenyl Degradation	biphenyl-2,3-diol 1,2-dioxygenase III-related protein
6	scaffold11|size4199117_1276248_1277165	Peripheral pathways for catabolism of aromatic compounds	Biphenyl Degradation	Acetaldehyde dehydrogenase, acetylating, (EC 1.2.1.10) in gene cluster for degradation of phenols, cresols, catechol
7	scaffold11|size4199117_1193388_1194917	Peripheral pathways for catabolism of aromatic compounds	Biphenyl Degradation	Acetaldehyde dehydrogenase (EC 1.2.1.10)
8	scaffold11|size4199117_1277155_1278180; scaffold11|size4199117_3045726_3046685	Peripheral pathways for catabolism of aromatic compounds	Biphenyl Degradation	4-hydroxy-2-oxovalerate aldolase (EC 4.1.3.39)
9	scaffold11|size4199117_3223019_3221727	Peripheral pathways for catabolism of aromatic compounds	Benzoate degradation	benzoate MFS transporter BenK
10	scaffold11|size4199117_486815_487504; scaffold11|size4199117_2347840_2347178	Metabolism of central aromatic intermediates	Catechol branch of beta-ketoadipate pathway	Succinyl-CoA:3-ketoacid-coenzyme A transferase subunit A (EC 2.8.3.5)
11	scaffold11|size4199117_487525_488193; scaffold11|size4199117_2347840_2347178	Metabolism of central aromatic intermediates	Catechol branch of beta-ketoadipate pathway	Succinyl-CoA:3-ketoacid-coenzyme A transferase subunit B (EC 2.8.3.5)
12	scaffold11|size4199117_1835207_1836721	Metabolism of central aromatic intermediates	Salicylate and gentisate catabolism	Salicylate hydroxylase (EC 1.14.13.1)
13	scaffold11|size4199117_1865502_1866278; scaffold11|size4199117_3955350_3954718	Metabolism of central aromatic intermediates	Salicylate and gentisate catabolism	Fumarylacetoacetate hydrolase family protein
14	scaffold11|size4199117_1269121_1270599; scaffold11|size4199117_2300574_2299138	Metabolism of Aromatic Compounds - no subcategory	Aromatic Amin Catabolism	4-hydroxyphenylacetate 3-monooxygenase (EC 1.14.13.3)

15	scaffold11|size4199117_3885013_3884402	Metabolism of Aromatic Compounds - no subcategory	Aromatic Amin Catabolism	Nitrilotriacetatemonooxygenase component B (EC 1.14.13.-)
16	scaffold11|size4199117_1865502_1866278; scaffold11|size4199117_3955350_3954718	Metabolism of Aromatic Compounds - no subcategory	Gentisate degradation	Fumarylacetoacetate hydrolase family protein

Table S13. Carbohydrate metabolism in *B.aryabhattai* AB211

No.	Contig_id/Size/start-end	Subcategory	Subsystem	Function (EC No.)
1.	scaffold11|size4199117_3411152_3410754	Central carbohydrate metabolism	Methylglyoxal Metabolism	Methylglyoxal synthase (EC 4.2.3.3)
2.	scaffold11|size4199117_3033077_3032652	Central carbohydrate metabolism	Methylglyoxal Metabolism	Lactoylglutathione lyase (EC 4.4.1.5)
3.	scaffold11|size4199117_818145_819569; scaffold11|size4199117_2343307_234185; scaffold11|size4199117_3347959_3346535	Central carbohydrate metabolism	Methylglyoxal Metabolism	Aldehyde dehydrogenase B (EC 1.2.1.22)
4.	scaffold11|size4199117_1459375_1458071; scaffold11|size4199117_2457891_2456443; scaffold11|size4199117_2475755_2474229; scaffold11|size4199117_2590559_2589159; scaffold11|size4199117_2715819_2714326; scaffold11|size4199117_2842083_2840617; scaffold11|size4199117_3219860_3218421	Central carbohydrate metabolism	Methylglyoxal Metabolism	Aldehyde dehydrogenase (EC 1.2.1.3)
5.	scaffold11|size4199117_480806_481762	Central carbohydrate metabolism	Methylglyoxal Metabolism	Hydroxyacylglutathione hydrolase (EC 3.1.2.6)
6.	scaffold11|size4199117_2478524_2477091	Central carbohydrate metabolism	Methylglyoxal Metabolism	Aldehyde dehydrogenase A (EC 1.2.1.22)
7.	scaffold11|size4199117_2014947_2016647; scaffold11|size4199117_3651623_3649902	Central carbohydrate metabolism	Pyruvate metabolism II: acetyl-CoA, acetogenesis from pyruvate	Pyruvate oxidase [ubiquinone, cytochrome] (EC 1.2.2.2)
8.	scaffold11|size4199117_3747202_3745739; scaffold11|size4199117_4002377_4003876	Central carbohydrate metabolism	Pyruvate metabolism II: acetyl-CoA, acetogenesis from pyruvate	Acetate permease ActP (cation/acetate symporter)
9.	scaffold11|size4199117_1193388_1194917	Central carbohydrate metabolism	Pyruvate metabolism II: acetyl-CoA, acetogenesis from pyruvate	Acetaldehyde dehydrogenase (EC 1.2.1.10)
10.	scaffold11|size4199117_88283_89998	Central carbohydrate metabolism	Pyruvate metabolism II: acetyl-CoA, acetogenesis from pyruvate	Acetyl-coenzyme A synthetase (EC 6.2.1.1)
11.	scaffold11|size4199117_3445873_3444461	Central carbohydrate metabolism	Pyruvate metabolism II: acetyl-CoA, acetogenesis from pyruvate	Dihydrolipoamide dehydrogenase of pyruvate dehydrogenase complex (EC 1.8.1.4)
12.	scaffold11|size4199117_3449377_3448262	Central carbohydrate	Pyruvate metabolism II:	Pyruvate dehydrogenase E1 component alpha

		metabolism	acetyl-CoA, acetogenesis from pyruvate	subunit (EC 1.2.4.1)
13.	scaffold11|size4199117_900664_901383	Central carbohydrate metabolism	Pyruvate metabolism II: acetyl-CoA, acetogenesis from pyruvate	NAD-dependent protein deacetylase of SIR2 family
14.	scaffold11|size4199117_86694_85531	Central carbohydrate metabolism	Pyruvate metabolism II: acetyl-CoA, acetogenesis from pyruvate	NAD-independent protein deacetylase AcuC
15.	scaffold11|size4199117_3448258_3447281	Central carbohydrate metabolism	Pyruvate metabolism II: acetyl-CoA, acetogenesis from pyruvate	Pyruvate dehydrogenase E1 component beta subunit (EC 1.2.4.1)
16.	scaffold11|size4199117_3447178_3445877	Central carbohydrate metabolism	Pyruvate metabolism II: acetyl-CoA, acetogenesis from pyruvate	Dihydrolipoamide acetyltransferase component of pyruvate dehydrogenase complex (EC 2.3.1.12)
17.	Scaffold10|size375231_274835_273864	Central carbohydrate metabolism	Pyruvate metabolism II: acetyl-CoA, acetogenesis from pyruvate	Phosphate acetyltransferase (EC 2.3.1.8)
18.	scaffold11|size4199117_108680_109870	Central carbohydrate metabolism	Pyruvate metabolism II: acetyl-CoA, acetogenesis from pyruvate	Acetate kinase (EC 2.7.2.1)
19.	scaffold11|size4199117_87998_87366	Central carbohydrate metabolism	Pyruvate metabolism II: acetyl-CoA, acetogenesis from pyruvate	Acetyltransferase AcuA, acetyl-CoA synthetase inhibitor
20.	Scaffold9|size151310_26289_24946; scaffold11|size4199117_938218_936848	Central carbohydrate metabolism	Pyruvate Alanine Serine Interconversions	D-serine/D-alanine/glycine transporter
21.	scaffold11|size4199117_657973_658860	Central carbohydrate metabolism	Pyruvate Alanine Serine Interconversions	L-serine dehydratase, alpha subunit (EC 4.3.1.17)
22.	scaffold11|size4199117_2205327_2206400	Central carbohydrate metabolism	Pyruvate Alanine Serine Interconversions	Branched-chain amino acid aminotransferase (EC 2.6.1.42)
23.	scaffold11|size4199117_657212_657874	Central carbohydrate metabolism	Pyruvate Alanine Serine Interconversions	L-serine dehydratase, beta subunit (EC 4.3.1.17)
24.	scaffold11|size4199117_2327809_2326508	Central carbohydrate metabolism	Pyruvate Alanine Serine Interconversions	Valine--pyruvate aminotransferase (EC 2.6.1.66)
25.	Scaffold5|size51851_11333_10143	Central carbohydrate metabolism	Pyruvate Alanine Serine Interconversions	Alanine racemase (EC 5.1.1.1)
26.	scaffold11|size4199117_2367088_2365970	Central carbohydrate metabolism	Pyruvate Alanine Serine Interconversions	D-amino acid dehydrogenase small subunit (EC 1.4.99.1)
27.	scaffold11|size4199117_1728909_1730159	Central carbohydrate	Pyruvate Alanine Serine	L-alanine:glyoxylate aminotransferase (EC

		metabolism	Interconversions	2.6.1.44)
28.	Scaffold10|size375231_284246_285379; scaffold11|size4199117_114674_115795	Central carbohydrate metabolism	Pyruvate Alanine Serine Interconversions	Alanine dehydrogenase (EC 1.4.1.1)
29.	scaffold11|size4199117_2665899_2667257	Central carbohydrate metabolism	Pyruvate Alanine Serine Interconversions	D-serine dehydratase (EC 4.3.1.18)
30.	scaffold11|size4199117_861559_860714	Central carbohydrate metabolism	Pyruvate Alanine Serine Interconversions	D-alanine aminotransferase (EC 2.6.1.21)
31.	scaffold11|size4199117_1863217_1865394	Central carbohydrate metabolism	Glyoxylate bypass	Malate synthase G (EC 2.3.3.9)
32.	scaffold11|size4199117_136342_137280; scaffold11|size4199117_1197291_1198247	Central carbohydrate metabolism	Glyoxylate bypass	Malate dehydrogenase (EC 1.1.1.37)
33.	scaffold11|size4199117_133827_134945; scaffold11|size4199117_1692443_1693546	Central carbohydrate metabolism	Glyoxylate bypass	Citrate synthase (si) (EC 2.3.3.1)
34.	scaffold11|size4199117_4196263_4194980	Central carbohydrate metabolism	Glyoxylate bypass	Isocitrate lyase (EC 4.1.3.1)
35.	scaffold11|size4199117_2163739_2166459	Central carbohydrate metabolism	Glyoxylate bypass	Aconitate hydratase (EC 4.2.1.3)
36.	scaffold11|size4199117_3027809_3026046	Central carbohydrate metabolism	Dihydroxyacetone kinases	Dihydroxyacetone kinase, ATP-dependent (EC 2.7.1.29)
37.	scaffold11|size4199117_1437089_1439017	Central carbohydrate metabolism	Glycolysis and Gluconeogenesis	Fructose-1,6-bisphosphatase, Bacillus type (EC 3.1.3.11)
38.	scaffold11|size4199117_898965_899924; scaffold11|size4199117_3143016_3142054	Central carbohydrate metabolism	Glycolysis and Gluconeogenesis	6-phosphofructokinase (EC 2.7.1.11)
39.	Scaffold10|size375231_119066_118311	Central carbohydrate metabolism	Glycolysis and Gluconeogenesis	Triosephosphate isomerase (EC 5.3.1.1)
40.	Scaffold10|size375231_244519_243662; scaffold11|size4199117_1371867_1372736	Central carbohydrate metabolism	Glycolysis and Gluconeogenesis	Fructose-bisphosphate aldolase class II (EC 4.1.2.13)
41.	Scaffold10|size375231_118311_116779	Central carbohydrate metabolism	Glycolysis and Gluconeogenesis	2,3-bisphosphoglycerate-independent phosphoglycerate mutase (EC 5.4.2.1)
42.	Scaffold10|size375231_13581_12232; scaffold12|size137365_12642_11305	Central carbohydrate metabolism	Glycolysis and Gluconeogenesis	Glucose-6-phosphate isomerase (EC 5.3.1.9)
43.	scaffold11|size4199117_145946_146977	Central carbohydrate metabolism	Glycolysis and Gluconeogenesis	NADPH-dependent glyceraldehyde-3-phosphate dehydrogenase (EC 1.2.1.13)
44.	scaffold11|size4199117_2897091_2895640	Central carbohydrate metabolism	Glycolysis and Gluconeogenesis	Non-phosphorylating glyceraldehyde-3-phosphate dehydrogenase (NADP) (EC 1.2.1.9)
45.	scaffold11|size4199117_394760_395728	Central carbohydrate metabolism	Glycolysis and Gluconeogenesis	Glucokinase (EC 2.7.1.2)

46.	scaffold11|size4199117_129507_131267; scaffold11|size4199117_3244996_3243944	Central carbohydrate metabolism	Glycolysis and Gluconeogenesis	Pyruvate kinase (EC 2.7.1.40)
47.	scaffold11|size4199117_1335286_1336179; scaffold11|size4199117_1336202_1337836; scaffold11|size4199117_2918440_2915834; scaffold11|size4199117_3153525_3151012	Central carbohydrate metabolism	Glycolysis and Gluconeogenesis	Phosphoenolpyruvate synthase (EC 2.7.9.2)
48.	Scaffold10|size375231_120426_119242	Central carbohydrate metabolism	Glycolysis and Gluconeogenesis	Phosphoglycerate kinase (EC 2.7.2.3)
49.	Scaffold10|size375231_116754_115459	Central carbohydrate metabolism	Glycolysis and Gluconeogenesis	Enolase (EC 4.2.1.11)
50.	Scaffold10|size375231_121743_120736	Central carbohydrate metabolism	Glycolysis and Gluconeogenesis	NAD-dependent glyceraldehyde-3-phosphate dehydrogenase (EC 1.2.1.12)
51.	scaffold11|size4199117_1223432_1224385	Central carbohydrate metabolism	Entner-Doudoroff Pathway	2-dehydro-3-deoxygluconate kinase (EC 2.7.1.45)
52.	Scaffold10|size375231_279403_280905; scaffold11|size4199117_1538005_1539507; scaffold11|size4199117_2786869_2785283; scaffold11|size4199117_3254796_3255167	Central carbohydrate metabolism	Entner-Doudoroff Pathway	Glucose-6-phosphate 1-dehydrogenase (EC 1.1.1.49)
53.	scaffold11|size4199117_1224403_1225065; scaffold11|size4199117_1786832_1787476	Central carbohydrate metabolism	Entner-Doudoroff Pathway	2-dehydro-3-deoxyphosphogluconate aldolase (EC 4.1.2.14)
54.	scaffold11|size4199117_1225052_1226200	Central carbohydrate metabolism	Entner-Doudoroff Pathway	Gluconate dehydratase (EC 4.2.1.39)
55.	scaffold11|size4199117_1460516_1461301; scaffold11|size4199117_3470851_3470087; scaffold11|size4199117_3749540_3748755; scaffold11|size4199117_3965547_3964762	Central carbohydrate metabolism	Entner-Doudoroff Pathway	Glucose 1-dehydrogenase (EC 1.1.1.47)
56.	scaffold11|size4199117_2302158_2301301	Central carbohydrate metabolism	Entner-Doudoroff Pathway	Gluconolactonase (EC 3.1.1.17)
57.	Scaffold9|size151310_110813_109749	Central carbohydrate metabolism	Entner-Doudoroff Pathway	6-phosphogluconolactonase (EC 3.1.1.31)
58.	scaffold11|size4199117_835361_836908; scaffold11|size4199117_4065083_4063542	Central carbohydrate metabolism	Entner-Doudoroff Pathway	Gluconokinase (EC 2.7.1.12)
60.	scaffold11|size4199117_2858057_2856678	Central carbohydrate metabolism	Dehydrogenase complexes	Dihydrolipoamide dehydrogenase of acetoin dehydrogenase (EC 1.8.1.4)
61.	scaffold11|size4199117_450547_451539	Central carbohydrate metabolism	Dehydrogenase complexes	Branched-chain alpha-keto acid dehydrogenase, E1 component, alpha subunit (EC 1.2.4.4)
62.	scaffold11|size4199117_2860360_2859326	Central carbohydrate metabolism	Dehydrogenase complexes	Acetoin dehydrogenase E1 component beta-subunit (EC 1.2.4.-)

63.	scaffold11|size4199117_2861371_2860379	Central carbohydrate metabolism	Dehydrogenase complexes	Acetoin dehydrogenase E1 component alpha-subunit (EC 1.2.4.-)
64.	scaffold11|size4199117_1831856_1833151	Central carbohydrate metabolism	Dehydrogenase complexes	Dihydrolipoamide succinyltransferase component (E2) of 2-oxoglutarate dehydrogenase complex (EC 2.3.1.61)
65.	scaffold11|size4199117_452551_453810	Central carbohydrate metabolism	Dehydrogenase complexes	Dihydrolipoamide acyltransferase component of branched-chain alpha-keto acid dehydrogenase complex (EC 2.3.1.168)
66.	scaffold11|size4199117_451552_452535	Central carbohydrate metabolism	ehydrogenase complexes	Branched-chain alpha-keto acid dehydrogenase, E1 component, beta subunit (EC 1.2.4.4)
67.	scaffold11|size4199117_2859300_2858074	Central carbohydrate metabolism	Dehydrogenase complexes	Dihydrolipoamide acetyltransferase component (E2) of acetoin dehydrogenase complex (EC 2.3.1.-)
68.	scaffold11|size4199117_449105_450526	Central carbohydrate metabolism	Dehydrogenase complexes	Dihydrolipoamide dehydrogenase of branched-chain alpha-keto acid dehydrogenase (EC 1.8.1.4)
69.	scaffold11|size4199117_1828978_1831839	Central carbohydrate metabolism	Dehydrogenase complexes	2-oxoglutarate dehydrogenase E1 component (EC 1.2.4.2)
70.	Scaffold10|size375231_33693_32200	Central carbohydrate metabolism	Dehydrogenase complexes	Cytosol aminopeptidase PepA (EC 3.4.11.1)
71.	scaffold11|size4199117_135027_136313	Central carbohydrate metabolism	TCA Cycle	Isocitrate dehydrogenase [NADP] (EC 1.1.1.42)
72.	scaffold11|size4199117_679607_680509	Central carbohydrate metabolism	TCA Cycle	Succinyl-CoA ligase [ADP-forming] alpha chain (EC 6.2.1.5)
73.	scaffold11|size4199117_182991_184778	Central carbohydrate metabolism	TCA Cycle	Succinate dehydrogenase flavoprotein subunit (EC 1.3.99.1)
74.	scaffold11|size4199117_184782_185555	Central carbohydrate metabolism	TCA Cycle	Succinate dehydrogenase iron-sulfur protein (EC 1.3.99.1)
75.	scaffold11|size4199117_1880532_1882034; scaffold11|size4199117_1977271_1978773	Central carbohydrate metabolism	TCA Cycle	Malate:quinone oxidoreductase (EC 1.1.5.4)
76.	Scaffold9|size151310_30775_29234	Central carbohydrate metabolism	TCA Cycle	Fumarate hydratase class I, aerobic (EC 4.2.1.2)
77.	scaffold11|size4199117_678428_679588	Central carbohydrate metabolism	TCA Cycle	Succinyl-CoA ligase [ADP-forming] beta chain (EC 6.2.1.5)
78.	scaffold11|size4199117_2398346_2396967	Central carbohydrate metabolism	TCA Cycle	Fumarate hydratase class II (EC 4.2.1.2)
79.	scaffold11|size4199117_2785133_2784078	Central carbohydrate metabolism	Pyruvate metabolism I: anaplerotic reactions, PEP	D-malic enzyme (EC 1.1.1.83)
80.	scaffold11|size4199117_38349_36760	Central carbohydrate	Pyruvate metabolism I:	Phosphoenolpyruvate carboxykinase [ATP] (EC

		metabolism	anaplerotic reactions, PEP	4.1.1.49)
81.	scaffold11|size4199117_2670620_2669289	Central carbohydrate metabolism	Pyruvate metabolism I: anaplerotic reactions, PEP	Malolactic enzyme (EC 1.-.-.-)
82.	Scaffold9|size151310_80396_79671; scaffold11|size4199117_2383345_2382638; scaffold11|size4199117_3967331_3966645	Central carbohydrate metabolism	Pyruvate metabolism I: anaplerotic reactions, PEP	Two-component response regulator, malate (EC 2.7.3.-)
83.	Scaffold9|size151310_81990_80389; scaffold11|size4199117_2384955_2383342; scaffold11|size4199117_3951318_3949729; scaffold11|size4199117_3968909_3967344	Central carbohydrate metabolism	Pyruvate metabolism I: anaplerotic reactions, PEP	Two-component sensor histidine kinase, malate (EC 2.7.3.-)
84.	scaffold11|size4199117_2086014_2086970; scaffold11|size4199117_2097706_2098662	Central carbohydrate metabolism	Pyruvate metabolism I: anaplerotic reactions, PEP	Malate permease
85.	scaffold11|size4199117_2691131_2689788; scaffold11|size4199117_2956098_2954692	Central carbohydrate metabolism	Pyruvate metabolism I: anaplerotic reactions, PEP	Malate Na(+) symporter
86.	scaffold11|size4199117_1120559_1122271; scaffold11|size4199117_2095990_2097693; scaffold11|size4199117_2692984_2691275	Central carbohydrate metabolism	Pyruvate metabolism I: anaplerotic reactions, PEP	NAD-dependent malic enzyme (EC 1.1.1.38)
87.	scaffold11|size4199117_3996509_3993741	Central carbohydrate metabolism	Pyruvate metabolism I: anaplerotic reactions, PEP	Phosphoenolpyruvate carboxylase (EC 4.1.1.31)
88.	scaffold11|size4199117_125037_126266	Central carbohydrate metabolism	Pyruvate metabolism I: anaplerotic reactions, PEP	NADP-dependent malic enzyme (EC 1.1.1.40)
89.	scaffold11|size4199117_3970224_3971492	Central carbohydrate metabolism	Pyruvate metabolism I: anaplerotic reactions, PEP	Aerobic C4-dicarboxylate transporter for fumarate, L-malate, D-malate, succunate, aspartate
90.	Scaffold10|size375231_141147_140500	Central carbohydrate metabolism	HPr kinase and hprK operon in Gram-positive organisms	Pyrophosphatase PpaX (EC 3.6.1.1)
91.	Scaffold10|size375231_142041_141208; scaffold11|size4199117_3043111_3043926	Central carbohydrate metabolism	HPr kinase and hprK operon in Gram-positive organisms	Prolipoprotein diacylglyceryl transferase (EC 2.4.99.-)
92.	Scaffold10|size375231_142993_142058	Central carbohydrate metabolism	HPr kinase and hprK operon in Gram-positive organisms	HPr kinase/phosphorylase (EC 2.7.1.-) (EC 2.7.4.-)
93.	scaffold11|size4199117_805541_807547; scaffold11|size4199117_1539536_1541530; scaffold12|size137365_13114_12857; scaffold12|size137365_13384_13160; scaffold12|size137365_13643_13494	Central carbohydrate metabolism	Pentose phosphate pathway	Transketolase (EC 2.2.1.1)
94.	scaffold11|size4199117_3148642_3147809	Central carbohydrate metabolism	Pentose phosphate pathway	Transketolase, N-terminal section (EC 2.2.1.1)
95.	Scaffold6|size49359_30618_29665	Central carbohydrate metabolism	Pentose phosphate pathway	Ribose-phosphate pyrophosphokinase (EC 2.7.6.1)

96.	Scaffold10|size375231_232211_231750; scaffold11|size4199117_3141729_3141265	Central carbohydrate metabolism	Pentose phosphate pathway	Ribose 5-phosphate isomerase B (EC 5.3.1.6)
97.	scaffold11|size4199117_3781596_3780928	Central carbohydrate metabolism	Pentose phosphate pathway	Ribose 5-phosphate isomerase A (EC 5.3.1.6)
98.	scaffold11|size4199117_3147809_3146862	Central carbohydrate metabolism	Pentose phosphate pathway	Transketolase, C-terminal section (EC 2.2.1.1)
99.	scaffold11|size4199117_651590_652246	Central carbohydrate metabolism	Pentose phosphate pathway	Ribulose-phosphate 3-epimerase (EC 5.1.3.1)
100.	Scaffold10|size375231_282360_280948; scaffold11|size4199117_1541561_1542454; scaffold11|size4199117_3460529_3459633; scaffold11|size4199117_4066008_4065109	Central carbohydrate metabolism	Pentose phosphate pathway	6-phosphogluconate dehydrogenase, decarboxylating (EC 1.1.1.44)
101.	Scaffold10|size375231_243524_242880; scaffold11|size4199117_1542504_1543172; scaffold11|size4199117_2388704_2388033; scaffold11|size4199117_2894177_2893509	Central carbohydrate metabolism	Pentose phosphate pathway	Transaldolase (EC 2.2.1.2)
102.	Scaffold10|size375231_47696_46707; scaffold11|size4199117_1325554_1326519	Central carbohydrate metabolism	Glycolate, glyoxylate interconversions	Glyoxylate reductase (EC 1.1.1.26)
103.	scaffold14|size51411_22562_23194	Central carbohydrate metabolism	Glycolate, glyoxylate interconversions	Phosphoglycolate phosphatase (EC 3.1.3.18)
104.	scaffold11|size4199117_1860249_1861610; scaffold11|size4199117_2081210_2082529	Central carbohydrate metabolism	Glycolate, glyoxylate interconversions	Glycolate dehydrogenase (EC 1.1.99.14), iron-sulfur subunit GlcF
105.	Scaffold10|size375231_47696_46707; scaffold11|size4199117_1325554_1326519	Central carbohydrate metabolism	Glycolate, glyoxylate interconversions	Hydroxypyruvate reductase (EC 1.1.1.81)
106.	scaffold11|size4199117_1861594_1863045	Central carbohydrate metabolism	Glycolate, glyoxylate interconversions	Glycolate dehydrogenase (EC 1.1.99.14), subunit GlcD
107.	Scaffold10|size375231_47696_46707; scaffold11|size4199117_1325554_1326519	Central carbohydrate metabolism	Glycolate, glyoxylate interconversions	Glyoxylate reductase (EC 1.1.1.79)
108.	scaffold11|size4199117_1858923_1860248	Central carbohydrate metabolism	Glycolate, glyoxylate interconversions	Glycolate dehydrogenase (EC 1.1.99.14), FAD-binding subunit GlcE
109.	scaffold11|size4199117_3617107_3617844	Aminosugars	Chitin and N-acetylglucosamine utilization	Glucosamine-6-phosphate deaminase (EC 3.5.99.6)
110.	scaffold11|size4199117_3356729_3357976	Aminosugars	Chitin and N-acetylglucosamine utilization	N-Acetyl-D-glucosamine ABC transport system, sugar-binding protein
111.	scaffold11|size4199117_3617902_3618627	Aminosugars	Chitin and N-acetylglucosamine utilization	Predicted transcriptional regulator of N-Acetylglucosamine utilization, GntR family
112.	scaffold11|size4199117_3788814_3787921	Aminosugars	Chitin and N-acetylglucosamine utilization	N-acetylglucosamine kinase of eukaryotic type (EC 2.7.1.59)

113.	scaffold11|size4199117_3615591_3613714	Aminosugars	Chitin and N-acetylglucosamine utilization	PTS system, N-acetylglucosamine-specific IIB component (EC 2.7.1.69)
114.	scaffold11|size4199117_3881849_3880971	Aminosugars	Chitin and N-acetylglucosamine utilization	N-Acetyl-D-glucosamine ABC transport system, permease protein 2
115.	scaffold11|size4199117_3615591_3613714	Aminosugars	Chitin and N-acetylglucosamine utilization	PTS system, N-acetylglucosamine-specific IIC component (EC 2.7.1.69)
116.	scaffold11|size4199117_2311174_2310293	Aminosugars	Chitin and N-acetylglucosamine utilization	N-Acetyl-D-glucosamine ABC transport system, permease protein 1
117.	scaffold11|size4199117_3615920_3617104	Aminosugars	Chitin and N-acetylglucosamine utilization	N-acetylglucosamine-6-phosphate deacetylase (EC 3.5.1.25)
118.	scaffold11|size4199117_1107965_1106736; scaffold11|size4199117_3205412_3204171	Di- and oligosaccharides	Sucrose utilization	Sucrose permease, major facilitator superfamily
119.	scaffold11|size4199117_1568315_1569388; scaffold11|size4199117_3205739_3206722	Di- and oligosaccharides	Sucrose utilization	Sucrose operon repressor ScrR, LacI family
120.	scaffold11|size4199117_877935_879314; scaffold11|size4199117_1245896_1244496	Di- and oligosaccharides	Sucrose utilization	PTS system, sucrose-specific IIC component (EC 2.7.1.69)
121.	scaffold11|size4199117_877935_879314; scaffold11|size4199117_1245896_1244496	Di- and oligosaccharides	Sucrose utilization	PTS system, sucrose-specific IIB component (EC 2.7.1.69)
122.	scaffold11|size4199117_2665525_2664587; scaffold11|size4199117_3634936_3633959	Di- and oligosaccharides	Sucrose utilization	Fructokinase (EC 2.7.1.4)
123.	Scaffold4|size36871_13669_11444; scaffold11|size4199117_1960685_1962007	Di- and oligosaccharides	Fructooligosaccharides(FOS) and Raffinose Utilization	Alpha-galactosidase (EC 3.2.1.22)
124.	Scaffold8|size138119_9947_8658; scaffold11|size4199117_1922773_1924056; scaffold11|size4199117_1957547_1958806	Di- and oligosaccharides	Fructooligosaccharides(FOS) and Raffinose Utilization	Multiple sugar ABC transporter, substrate-binding protein
125.	scaffold11|size4199117_1921875_1922702; scaffold11|size4199117_1959828_1960658	Di- and oligosaccharides	Fructooligosaccharides(FOS) and Raffinose Utilization	Multiple sugar ABC transporter, membrane-spanning permease protein MsmG
126.	scaffold11|size4199117_1920913_1921875; scaffold11|size4199117_1958896_1959828	Di- and oligosaccharides	Fructooligosaccharides(FOS) and Raffinose Utilization	Multiple sugar ABC transporter, membrane-spanning permease protein MsmF
127.	Scaffold8|size138119_53850_54959; scaffold11|size4199117_3355601_3356692	Di- and oligosaccharides	Fructooligosaccharides(FOS) and Raffinose Utilization	Multiple sugar ABC transporter, ATP-binding protein
128.	scaffold11|size4199117_1956201_1957235	Di- and oligosaccharides	Fructooligosaccharides(FOS) and Raffinose Utilization	MSM (multiple sugar metabolism) operon regulatory protein
129.	scaffold11|size4199117_3849017_3848301	Di- and oligosaccharides	Trehalose Uptake and Utilization	Trehalose operon transcriptional repressor
130.	scaffold11|size4199117_3491284_3489230	Di- and oligosaccharides	Trehalose Uptake and Utilization	PTS system, glucose-specific IIB component (EC 2.7.1.69)
131.	scaffold11|size4199117_3852269_3850806	Di- and oligosaccharides	Trehalose Uptake and	PTS system, trehalose-specific IIC component (EC

			Utilization	2.7.1.69)
132.	scaffold11|size4199117_2333676_2332471	Di- and oligosaccharides	Trehalose Uptake and Utilization	Glucose/mannose:H+ symporter GlcP
133.	Scaffold10|size375231_289145_288438	Di- and oligosaccharides	Trehalose Uptake and Utilization	Beta-phosphoglucomutase (EC 5.4.2.6)
134.	scaffold11|size4199117_3850790_3849105	Di- and oligosaccharides	Trehalose Uptake and Utilization	Trehalose-6-phosphate hydrolase (EC 3.2.1.93)
135.	scaffold11|size4199117_876394_876891	Di- and oligosaccharides	Trehalose Uptake and Utilization	PTS system, glucose-specific IIA component (EC 2.7.1.69)
136.	scaffold11|size4199117_3852269_3850806	Di- and oligosaccharides	Trehalose Uptake and Utilization	PTS system, trehalose-specific IIB component (EC 2.7.1.69)
137.	scaffold11|size4199117_3679752_3678682; scaffold11|size4199117_3763526_3762546; scaffold11|size4199117_4125638_4124754; scaffold13|size87933_43617_42649; scaffold13|size87933_64047_65081;	Di- and oligosaccharides	Lactose and Galactose Uptake and Utilization	UDP-glucose 4-epimerase (EC 5.1.3.2)
138.	scaffold11|size4199117_2560151_2563255	Di- and oligosaccharides	Lactose and Galactose Uptake and Utilization	Beta-galactosidase (EC 3.2.1.23)
139.	scaffold11|size4199117_3531550_3532317; scaffold11|size4199117_4129611_4128088	Di- and oligosaccharides	Lactose and Galactose Uptake and Utilization	Galactose-1-phosphate uridylyltransferase (EC 2.7.7.10)
140.	scaffold11|size4199117_4130820_4129642	Di- and oligosaccharides	Lactose and Galactose Uptake and Utilization	Galactokinase (EC 2.7.1.6)
141.	scaffold11|size4199117_2891541_2890486	Di- and oligosaccharides	Lactose and Galactose Uptake and Utilization	Aldose 1-epimerase (EC 5.1.3.3)
142.	scaffold11|size4199117_3254631_3254266; scaffold11|size4199117_3917986_3918351	One-carbon Metabolism	Formaldehyde assimilation: Ribulose monophosphate pathway	Transcriptional regulator HxlR, formaldehyde assimilation
143.	scaffold11|size4199117_1789326_1789874; scaffold11|size4199117_3919260_3919817	One-carbon Metabolism	Formaldehyde assimilation: Ribulose monophosphate pathway	6-phospho-3-hexuloisomerase
144.	scaffold11|size4199117_1728909_1730159	One-carbon Metabolism	Serine-glyoxylate cycle	Serine--pyruvate aminotransferase (EC 2.6.1.51)
145.	Scaffold10|size375231_231045_229801	One-carbon Metabolism	Serine-glyoxylate cycle	Serine hydroxymethyltransferase (EC 2.1.2.1)
146.	scaffold11|size4199117_487525_488193; scaffold11|size4199117_2347840_2347178	One-carbon Metabolism	Serine-glyoxylate cycle	Succinyl-CoA:3-ketoacid-coenzyme A transferase subunit B (EC 2.8.3.5)
147.	scaffold11|size4199117_3563730_3562987	One-carbon Metabolism	Serine-glyoxylate cycle	Acetoacetyl-CoA reductase (EC 1.1.1.36)
148.	scaffold11|size4199117_486815_487504; scaffold11|size4199117_2348498_2347842	One-carbon Metabolism	Serine-glyoxylate cycle	Succinyl-CoA:3-ketoacid-coenzyme A transferase subunit A (EC 2.8.3.5)

149.	scaffold11|size4199117_3564411_3563812	One-carbon Metabolism	Serine-glyoxylate cycle	polyhydroxyalkanoate synthesis repressor PhaR
150.	scaffold11|size4199117_464754_466304	One-carbon Metabolism	Serine-glyoxylate cycle	Propionyl-CoA carboxylase beta chain (EC 6.4.1.3)
151.	Scaffold10|size375231_251983_248741; scaffold11|size4199117_453969_456005; scaffold11|size4199117_455998_458148	One-carbon Metabolism	Serine-glyoxylate cycle	Methylmalonyl-CoA mutase (EC 5.4.99.2)
152.	scaffold11|size4199117_3498398_3500236	One-carbon Metabolism	Serine-glyoxylate cycle	5,10-methylenetetrahydrofolate reductase (EC 1.5.1.20)
153.	scaffold11|size4199117_2625573_2624719	One-carbon Metabolism	Serine-glyoxylate cycle	Methenyltetrahydrofolate cyclohydrolase (EC 3.5.4.9)
154.	scaffold11|size4199117_2768475_2766787	One-carbon Metabolism	Serine-glyoxylate cycle	Formate--tetrahydrofolate ligase (EC 6.3.4.3)
155.	scaffold11|size4199117_588180_589721	One-carbon Metabolism	Serine-glyoxylate cycle	Methylcrotonyl-CoA carboxylase carboxyl transferase subunit (EC 6.4.1.4)
156.	scaffold11|size4199117_391136_391702	One-carbon Metabolism	Serine-glyoxylate cycle	5-formyltetrahydrofolate cyclo-ligase (EC 6.3.3.2)
157.	scaffold11|size4199117_2625573_2624719	One-carbon Metabolism	Serine-glyoxylate cycle	Methylenetetrahydrofolate dehydrogenase (NADP+) (EC 1.5.1.5)
158.	scaffold11|size4199117_970388_969870	One-carbon Metabolism	Serine-glyoxylate cycle	cytosolic long-chain acyl-CoA thioester hydrolase family protein
159.	Scaffold10|size375231_72917_71739; scaffold11|size4199117_485389_486576; scaffold11|size4199117_2224633_2223533; scaffold11|size4199117_2347178_2345988	One-carbon Metabolism	Serine-glyoxylate cycle	Acetyl-CoA acetyltransferase (EC 2.3.1.9)
160.	Scaffold10|size375231_72917_71739; scaffold11|size4199117_485389_486576; scaffold11|size4199117_2224633_2223533; scaffold11|size4199117_2347178_2345988	One-carbon Metabolism	Serine-glyoxylate cycle	3-ketoacyl-CoA thiolase (EC 2.3.1.16)
161.	scaffold11|size4199117_1387191_1387922	One-carbon Metabolism	Serine-glyoxylate cycle	Glycerate kinase (EC 2.7.1.31)
162.	scaffold11|size4199117_837040_837942	One-carbon Metabolism	One-carbon metabolism by tetrahydropterines	Formyltetrahydrofolate deformylase (EC 3.5.1.10)
163.	scaffold11|size4199117_2019404_2020282; scaffold11|size4199117_3647334_3646432; scaffold11|size4199117_4127161_4128045	Organic acids	Glycerate metabolism	2-hydroxy-3-oxopropionate reductase (EC 1.1.1.60)
164.	scaffold11|size4199117_1950543_1951811	Organic acids	Glycerate metabolism	D-glycerate transporter (predicted)
165.	scaffold11|size4199117_2785133_2784078	Organic acids	Glycerate metabolism	Tartrate decarboxylase (EC 4.1.1.73)
166.	scaffold11|size4199117_3552861_3551422	Organic acids	Lactate utilization	Predicted L-lactate dehydrogenase, Iron-sulfur cluster-binding subunit YkgF

167.	Scaffold8|size138119_90632_92206; scaffold11|size4199117_1858540_1856840	Organic acids	Lactate utilization	L-lactate permease
168.	scaffold11|size4199117_3551444_3550731	Organic acids	Lactate utilization	Predicted L-lactate dehydrogenase, hypothetical protein subunit YkgG
169.	scaffold11|size4199117_3553601_3552879	Organic acids	Lactate utilization	Predicted L-lactate dehydrogenase, Fe-S oxidoreductase subunit YkgE
170.	scaffold11|size4199117_3557319_3556600	Organic acids	Lactate utilization	Lactate-responsive regulator LldR in Firmicutes, GntR family
171.	scaffold11|size4199117_4012406_4011540	Organic acids	Malonate decarboxylase	Triphosphoribosyl-dephospho-CoA synthetase (EC 2.7.8.25)
172.	scaffold11|size4199117_4099269_4098508	Organic acids	Malonate decarboxylase	Malonate transporter, MadM subunit
173.	scaffold11|size4199117_4009539_4008931	Organic acids	Malonate decarboxylase	Phosphoribosyl-dephospho-CoA transferase (EC 2.7.7.-)
174.	scaffold11|size4199117_4011516_4011214	Organic acids	Malonate decarboxylase	Malonate decarboxylase delta subunit
175.	scaffold11|size4199117_4011227_4009527	Organic acids	Malonate decarboxylase	Malonate decarboxylase beta subunit
176.	scaffold11|size4199117_4015028_4014102	Organic acids	Malonate decarboxylase	Malonyl CoA acyl carrier protein transacylase (EC 2.3.1.39)
177.	scaffold11|size4199117_4099679_4099266	Organic acids	Malonate decarboxylase	Malonate transporter, MadL subunit
178.	scaffold11|size4199117_4014064_4012412	Organic acids	Malonate decarboxylase	Malonate decarboxylase alpha subunit
179.	scaffold11|size4199117_4011227_4009527	Organic acids	Malonate decarboxylase	Malonate decarboxylase gamma subunit
180.	scaffold11|size4199117_4021613_4022326	Organic acids	Alpha-acetolactate operon	Alpha-acetolactate decarboxylase (EC 4.1.1.5)
181.	scaffold11|size4199117_4019917_4021596	Organic acids	Alpha-acetolactate operon	Acetolactate synthase, catabolic (EC 2.2.1.6)
182.	Scaffold8|size138119_68809_69804; Scaffold8|size138119_93296_92304; scaffold11|size4199117_1148717_1149919; scaffold11|size4199117_1193388_1194917; scaffold11|size4199117_1233453_1234493; scaffold11|size4199117_1367241_1366180; scaffold11|size4199117_1949157_1947973; scaffold11|size4199117_2074581_2075771; scaffold11|size4199117_2473009_2471822; scaffold11|size4199117_2474207_2473038; scaffold11|size4199117_3908075_3907092	Fermentation	Butanol Biosynthesis	Alcohol dehydrogenase (EC 1.1.1.1)
183.	Scaffold10|size375231_21251_20088; scaffold11|size4199117_1911492_1912664; scaffold11|size4199117_2335029_2333857	Fermentation	Butanol Biosynthesis	NADH-dependent butanol dehydrogenase A (EC 1.1.1.-)

184.	Scaffold8|size138119_1254_478; Scaffold8|size138119_42290_41487; scaffold11|size4199117_174389_175162; scaffold11|size4199117_584185_584952; scaffold11|size4199117_2352356_2351583; scaffold11|size4199117_2357944_2357162	Fermentation	Butanol Biosynthesis	Enoyl-CoA hydratase (EC 4.2.1.17)
185.	Scaffold10|size375231_255791_254937; scaffold11|size4199117_2358825_2357941	Fermentation	Butanol Biosynthesis	3-hydroxybutyryl-CoA dehydrogenase (EC 1.1.1.157)
186.	scaffold11|size4199117_204286_206004; scaffold11|size4199117_3077198_3075603; scaffold11|size4199117_3855293_3853536	Fermentation	Acetolactate synthase subunits	Acetolactate synthase large subunit (EC 2.2.1.6)
187.	scaffold11|size4199117_206001_206513	Fermentation	Acetolactate synthase subunits	Acetolactate synthase small subunit (EC 2.2.1.6)
188.	Scaffold8|size138119_89607_90563	Fermentation	Fermentations: Lactate	L-lactate dehydrogenase (EC 1.1.1.27)
189.	scaffold11|size4199117_2904407_2902995	Fermentation	Acetyl-CoA fermentation to Butyrate	Acetoacetate metabolism regulatory protein AtoC
190.	scaffold11|size4199117_1005746_1006261	Fermentation	Acetyl-CoA fermentation to Butyrate	3-hydroxybutyryl-CoA dehydratase (EC 4.2.1.55)
191.	scaffold11|size4199117_447991_449076	Fermentation	Acetyl-CoA fermentation to Butyrate	Butyrate kinase (EC 2.7.2.7)
192.	scaffold11|size4199117_175988_176968	Fermentation	Acetyl-CoA fermentation to Butyrate	Electron transfer flavoprotein, alpha subunit
193.	scaffold11|size4199117_856089_856751	Fermentation	Acetyl-CoA fermentation to Butyrate	Acetyl-CoA:acetoacetyl-CoA transferase, beta subunit (EC 2.8.3.8)
194.	scaffold11|size4199117_175181_175954	Fermentation	Acetyl-CoA fermentation to Butyrate	Electron transfer flavoprotein, beta subunit
195.	Scaffold10|size375231_75308_72930; scaffold11|size4199117_2358825_2357941	Fermentation	Acetyl-CoA fermentation to Butyrate	3-hydroxyacyl-CoA dehydrogenase (EC 1.1.1.35)
196.	scaffold11|size4199117_855403_856104	Fermentation	Acetyl-CoA fermentation to Butyrate	Acetyl-CoA:acetoacetyl-CoA transferase, alpha subunit (EC 2.8.3.8)
197.	scaffold11|size4199117_1420874_1419828	Fermentation	Acetoin, butanediol metabolism	2,3-butanediol dehydrogenase, R-alcohol forming, (R)- and (S)-acetoin-specific (EC 1.1.1.4)
198.	scaffold11|size4199117_2856414_2854537	Fermentation	Acetoin, butanediol metabolism	Transcriptional activator of acetoin dehydrogenase operon AcoR
199.	Scaffold10|size375231_322258_320825	CO2 fixation	Photorespiration (oxidative C2 cycle)	2-oxoglutarate/malate translocator
200.	scaffold11|size4199117_408627_410105	CO2 fixation	Photorespiration (oxidative C2 cycle)	Glycine dehydrogenase [decarboxylating] (glycine cleavage system P2 protein) (EC 1.4.4.2)

201.	scaffold11|size4199117_407288_408634	CO2 fixation	Photorespiration (oxidative C2 cycle)	Glycine dehydrogenase [decarboxylating] (glycine cleavage system P1 protein) (EC 1.4.4.2)
202.	Scaffold10|size375231_69323_68940	CO2 fixation	Photorespiration (oxidative C2 cycle)	Glycine cleavage system H protein
203.	Scaffold10|size375231_317129_318589	CO2 fixation	Photorespiration (oxidative C2 cycle)	Catalase (EC 1.11.1.6)
204.	scaffold11|size4199117_406167_407267	CO2 fixation	Photorespiration (oxidative C2 cycle)	Aminomethyltransferase (glycine cleavage system T protein) (EC 2.1.2.10)
205.	Scaffold4|size36871_7415_6603	Sugar alcohols	Glycerol and Glycerol-3-phosphate Uptake and Utilization	Glycerol-3-phosphate ABC transporter, permease protein UgpE (TC 3.A.1.1.3)
206.	scaffold11|size4199117_884985_884167	Sugar alcohols	Glycerol and Glycerol-3-phosphate Uptake and Utilization	Glycerophosphoryl diester phosphodiesterase, periplasmic (EC 3.1.4.46)
207.	scaffold11|size4199117_545743_546774	Sugar alcohols	Glycerol and Glycerol-3-phosphate Uptake and Utilization	Glycerol-3-phosphate dehydrogenase [NAD(P)+] (EC 1.1.1.94)
208.	Scaffold4|size36871_8347_7412	Sugar alcohols	Glycerol and Glycerol-3-phosphate Uptake and Utilization	Glycerol-3-phosphate ABC transporter, permease protein UgpA (TC 3.A.1.1.3)
209.	Scaffold8|size138119_78914_77424	Sugar alcohols	Glycerol and Glycerol-3-phosphate Uptake and Utilization	Glycerol kinase (EC 2.7.1.30)
210.	Scaffold5|size51851_12994_13596	Sugar alcohols	Glycerol and Glycerol-3-phosphate Uptake and Utilization	GlpG protein (membrane protein of glp regulon)
211.	Scaffold4|size36871_6589_5249	Sugar alcohols	Glycerol and Glycerol-3-phosphate Uptake and Utilization	Glycerol-3-phosphate ABC transporter, periplasmic glycerol-3-phosphate-binding protein (TC 3.A.1.1.3)
212.	Scaffold4|size36871_5162_4425; scaffold11|size4199117_442396_443124; scaffold11|size4199117_2059950_2059075	Sugar alcohols	Glycerol and Glycerol-3-phosphate Uptake and Utilization	Glycerophosphoryl diester phosphodiesterase (EC 3.1.4.46)
213.	Scaffold8|size138119_77237_75558	Sugar alcohols	Glycerol and Glycerol-3-phosphate Uptake and Utilization	Aerobic glycerol-3-phosphate dehydrogenase (EC 1.1.5.3)
214.	Scaffold8|size138119_79808_78981	Sugar alcohols	Glycerol and Glycerol-3-phosphate Uptake and Utilization	Glycerol uptake facilitator protein

215.	Scaffold4|size36871_9459_8344	Sugar alcohols	Glycerol and Glycerol-3-phosphate Uptake and Utilization	Glycerol-3-phosphate ABC transporter, ATP-binding protein UgpC (TC 3.A.1.1.3)
216.	scaffold11|size4199117_3488945_3487224	Sugar alcohols	Mannitol Utilization	Phosphoenolpyruvate-protein phosphotransferase of PTS system (EC 2.7.3.9)
217.	Scaffold5|size51851_36820_34730	Sugar alcohols	Mannitol Utilization	Mannitol operon activator, BglG family
218.	Scaffold5|size51851_38773_36881	Sugar alcohols	Mannitol Utilization	PTS system, mannitol-specific IIC component (EC 2.7.1.69)
219.	Scaffold5|size51851_34724_33582	Sugar alcohols	Mannitol Utilization	Mannitol-1-phosphate 5-dehydrogenase (EC 1.1.1.17)
220.	Scaffold5|size51851_38773_36881	Sugar alcohols	Mannitol Utilization	PTS system, mannitol-specific IIB component (EC 2.7.1.69)
221.	Scaffold8|size138119_122069_121047	Sugar alcohols	Inositol catabolism	Myo-inositol 2-dehydrogenase 1 (EC 1.1.1.18)
222.	scaffold11|size4199117_1369552_1370970; scaffold12|size137365_81124_82467; scaffold12|size137365_97205_98629	Sugar alcohols	Inositol catabolism	Major myo-inositol transporter IolT
223.	Scaffold8|size138119_115331_114336; Scaffold8|size138119_124842_123838; scaffold11|size4199117_1383436_1384485; scaffold12|size137365_77254_78261; scaffold12|size137365_98819_99814	Sugar alcohols	Inositol catabolism	Predicted transcriptional regulator of the myo-inositol catabolic operon
224.	scaffold11|size4199117_1382302_1383183	Sugar alcohols	Inositol catabolism	Glyceraldehyde-3-phosphate ketol-isomerase (EC 5.3.1.1)
225.	scaffold11|size4199117_1373115_1373870	Sugar alcohols	Inositol catabolism	Transcriptional repressor of the myo-inositol catabolic operon DeoR family
226.	Scaffold8|size138119_116288_115392; scaffold11|size4199117_1368638_1369531; scaffold11|size4199117_3880777_3880043	Sugar alcohols	Inositol catabolism	Inosose dehydratase (EC 4.2.1.44)
227.	scaffold11|size4199117_1370993_1371850; scaffold11|size4199117_2580502_2579534	Sugar alcohols	Inositol catabolism	Inosose isomerase (EC 5.3.99.-)
228.	Scaffold8|size138119_119877_118420; scaffold11|size4199117_1378353_1379810	Sugar alcohols	Inositol catabolism	Methylmalonate-semialdehyde dehydrogenase [inositol] (EC 1.2.1.27)
229.	scaffold11|size4199117_1381175_1382212; scaffold11|size4199117_2582546_2581521; scaffold12|size137365_79064_80083; scaffold12|size137365_95974_97026	Sugar alcohols	Inositol catabolism	Myo-inositol 2-dehydrogenase (EC 1.1.1.18)
230.	Scaffold8|size138119_114171_113383;	Sugar alcohols	Inositol catabolism	5-deoxy-glucuronate isomerase (EC 5.3.1.-)

	scaffold11|size4199117_1376487_1377305			
231.	Scaffold8|size138119_120970_119963; scaffold11|size4199117_1377321_1378328	Sugar alcohols	Inositol catabolism	5-keto-2-deoxygluconokinase (EC 2.7.1.92)
232.	Scaffold8|size138119_118307_116373; scaffold11|size4199117_1374470_1376392	Sugar alcohols	Inositol catabolism	Epi-inositol hydrolase (EC 3.7.1.-)
233.	Scaffold10|size375231_181271_180849	Carbohydrates - no subcategory	Carbon storage regulator	Flagellar assembly factor FliW
234.	Scaffold10|size375231_180824_180594	Carbohydrates - no subcategory	Carbon storage regulator	Carbon storage regulator
235.	scaffold11|size4199117_2474_3625	Polysaccharides	Glycogen metabolism	Glucose-1-phosphate adenylyltransferase (EC 2.7.7.27)
236.	scaffold11|size4199117_562_2523	Polysaccharides	Glycogen metabolism	1,4-alpha-glucan (glycogen) branching enzyme, GH-13-type (EC 2.4.1.18)
237.	scaffold11|size4199117_3648_4673	Polysaccharides	Glycogen metabolism	Glycogen biosynthesis protein GlgD, glucose-1-phosphate adenylyltransferase family
238.	scaffold11|size4199117_6114_8525	Polysaccharides	Glycogen metabolism	Glycogen phosphorylase (EC 2.4.1.1)
239.	scaffold11|size4199117_63000_65141	Polysaccharides	Glycogen metabolism	Glycogen debranching enzyme (EC 3.2.1.-)
240.	scaffold11|size4199117_386104_388422	Polysaccharides	Glycogen metabolism	Glycogen branching enzyme, GH-57-type, archaeal (EC 2.4.1.18)
241.	scaffold11|size4199117_4670_6100	Polysaccharides	Glycogen metabolism	Glycogen synthase, ADP-glucose transglucosylase (EC 2.4.1.21)
242.	Scaffold10|size375231_310969_310193	Polysaccharides	Alpha-Amylase locus in Streptocococcus	putative esterase
243.	scaffold11|size4199117_2312476_2311190	Polysaccharides	Alpha-Amylase locus in Streptocococcus	Maltose/maltodextrin ABC transporter, substrate binding periplasmic protein MalE
244.	Scaffold8|size138119_75393_73639 scaffold11|size4199117_388419_390698; scaffold11|size4199117_3693806_3692082	Monosaccharides	Mannose Metabolism	Phosphomannomutase (EC 5.4.2.8)
245.	scaffold11|size4199117_3195075_3194581	Monosaccharides	Mannose Metabolism	PTS system, mannose-specific IIB component (EC 2.7.1.69)
246.	scaffold11|size4199117_1353530_1352319	Monosaccharides	Mannose Metabolism	D-mannose isomerase (EC 5.3.1.7)
247.	scaffold11|size4199117_3195075_3194581	Monosaccharides	Mannose Metabolism	PTS system, mannose-specific IIA component (EC 2.7.1.69)
248.	scaffold11|size4199117_942354_942713	Monosaccharides	Mannose Metabolism	Mannose-6-phosphate isomerase (EC 5.3.1.8)
249.	scaffold11|size4199117_388419_390698	Monosaccharides	Mannose Metabolism	Mannose-1-phosphate guanylyltransferase (EC 2.7.7.13)

250.	scaffold11|size4199117_1783823_1784566	Monosaccharides	2-Ketogluconate Utilization	Epimerase KguE
251.	scaffold11|size4199117_1785525_1786805	Monosaccharides	2-Ketogluconate Utilization	2-ketogluconate transporter
252.	scaffold11|size4199117_1787578_1788588	Monosaccharides	2-Ketogluconate Utilization	2-ketogluconate utilization repressor PtxS
253.	scaffold11|size4199117_1784563_1785522	Monosaccharides	2-Ketogluconate Utilization	2-ketogluconate kinase (EC 2.7.1.13)
254.	scaffold11|size4199117_3891555_3890677	Monosaccharides	Deoxyribose and Deoxynucleoside Catabolism	Ribokinase (EC 2.7.1.15)
255.	scaffold11|size4199117_493271_494452	Monosaccharides	Deoxyribose and Deoxynucleoside Catabolism	Phosphopentomutase (EC 5.4.2.7)
256.	scaffold11|size4199117_1933618_1934919	Monosaccharides	Deoxyribose and Deoxynucleoside Catabolism	Pyrimidine-nucleoside phosphorylase (EC 2.4.2.2)
257.	scaffold11|size4199117_494469_495299; scaffold11|size4199117_869616_870320; scaffold11|size4199117_2141505_2141963	Monosaccharides	Deoxyribose and Deoxynucleoside Catabolism	Purine nucleoside phosphorylase (EC 2.4.2.1)
258.	scaffold11|size4199117_1932156_1933106	Monosaccharides	Deoxyribose and Deoxynucleoside Catabolism	Deoxyribonucleoside regulator DeoR (transcriptional repressor)
259.	scaffold11|size4199117_1081250_1082782	Monosaccharides	Deoxyribose and Deoxynucleoside Catabolism	Predicted nucleoside ABC transporter, ATP-binding component
260.	scaffold11|size4199117_1934941_1935615	Monosaccharides	Deoxyribose and Deoxynucleoside Catabolism	Deoxyribose-phosphate aldolase (EC 4.1.2.4)
261.	scaffold11|size4199117_1080158_1081168	Monosaccharides	Deoxyribose and Deoxynucleoside Catabolism	Predicted nucleoside ABC transporter, substrate-binding component
262.	scaffold11|size4199117_3644889_3643531	Monosaccharides	D-gluconate and ketogluconates metabolism	Gluconate transporter family protein
263.	scaffold11|size4199117_4061465_4060137	Monosaccharides	D-gluconate and ketogluconates metabolism	Gluconate permease
264.	scaffold11|size4199117_2599946_2598075	Monosaccharides	Fructose utilization	PTS system, fructose-specific IIB component (EC 2.7.1.69)
265.	scaffold11|size4199117_2599946_2598075	Monosaccharides	Fructose utilization	PTS system, fructose-specific IIA component (EC 2.7.1.69)
266.	scaffold11|size4199117_2599946_2598075	Monosaccharides	Fructose utilization	PTS system, fructose-specific IIC component (EC 2.7.1.69)
267.	scaffold11|size4199117_58324_58103; scaffold11|size4199117_2601622_2600870	Monosaccharides	Fructose utilization	Transcriptional repressor of the fructose operon, DeoR family
268.	scaffold11|size4199117_2600873_2599959	Monosaccharides	Fructose utilization	1-phosphofructokinase (EC 2.7.1.56)
269.	scaffold11|size4199117_2837495_2836791	Monosaccharides	D-galactarate, D-glucarate and D-glycerate catabolism - gjo	Predicted 5-dehydro-4-deoxyglucarate regulator YcbG

270.	scaffold11|size4199117_2840503_2839124	Monosaccharides	D-galactarate, D-glucarate and D-glycerate catabolism - gjo	D-glucarate permease
271.	scaffold11|size4199117_2839087_2837714	Monosaccharides	D-galactarate, D-glucarate and D-glycerate catabolism - gjo	Glucarate dehydratase (EC 4.2.1.40)
272.	scaffold11|size4199117_1949316_1950416	Monosaccharides	D-galactarate, D-glucarate and D-glycerate catabolism - gjo	Sugar diacid utilization regulator SdaR
273.	scaffold11|size4199117_2845345_2846880	Monosaccharides	D-galactarate, D-glucarate and D-glycerate catabolism - gjo	D-galactarate dehydratase (EC 4.2.1.42)
274.	scaffold11|size4199117_2843055_2842123	Monosaccharides	D-galactarate, D-glucarate and D-glycerate catabolism - gjo	5-dehydro-4-deoxyglucarate dehydratase (EC 4.2.1.41)
275.	scaffold11|size4199117_1866332_1867126	Monosaccharides	D-Galacturonate and D-Glucuronate Utilization	Transcriptional regulator KdgR, KDG operon repressor
276.	scaffold11|size4199117_1109579_1111288; scaffold11|size4199117_3363625_3361958	Monosaccharides	D-Galacturonate and D-Glucuronate Utilization	Alpha-glucosidase (EC 3.2.1.20)
277.	scaffold11|size4199117_2825960_2824497	Monosaccharides	D-Galacturonate and D-Glucuronate Utilization	Altronate oxidoreductase (EC 1.1.1.58)
278.	scaffold11|size4199117_2822951_2821554	Monosaccharides	D-Galacturonate and D-Glucuronate Utilization	Polygalacturonase (EC 3.2.1.15)
279.	scaffold11|size4199117_1903725_1904486	Monosaccharides	D-Galacturonate and D-Glucuronate Utilization	2-deoxy-D-gluconate 3-dehydrogenase (EC 1.1.1.125)
280.	scaffold11|size4199117_2828575_2827193	Monosaccharides	D-Galacturonate and D-Glucuronate Utilization	Glucuronide transporter UidB
281.	scaffold11|size4199117_2830044_2828623	Monosaccharides	D-Galacturonate and D-Glucuronate Utilization	Uronate isomerase (EC 5.3.1.12)
282.	scaffold11|size4199117_2827090_2826077	Monosaccharides	D-Galacturonate and D-Glucuronate Utilization	Hexuronate utilization operon transcriptional repressor ExuR
283.	scaffold11|size4199117_3538032_3536527; scaffold11|size4199117_3890272_3888791	Monosaccharides	D-ribose utilization	Ribose ABC transport system, ATP-binding protein RbsA (TC 3.A.1.2.1)
284.	scaffold11|size4199117_2584425_2583394; scaffold11|size4199117_3892537_3891548	Monosaccharides	D-ribose utilization	Ribose operon repressor
285.	scaffold11|size4199117_3536530_3535496; scaffold11|size4199117_3888789_3887830	Monosaccharides	D-ribose utilization	Ribose ABC transport system, permease protein RbsC (TC 3.A.1.2.1)
286.	scaffold11|size4199117_3887818_3886898	Monosaccharides	D-ribose utilization	Ribose ABC transport system, periplasmic ribose-binding protein RbsB (TC 3.A.1.2.1)
287.	scaffold11|size4199117_3890676_3890287	Monosaccharides	D-ribose utilization	Ribose ABC transport system, high affinity permease RbsD (TC 3.A.1.2.1)
288.	scaffold11|size4199117_2884871_2883534	Monosaccharides	Xylose utilization	Xylose isomerase (EC 5.3.1.5)

289.	scaffold11|size4199117_2883475_2881973	Monosaccharides	Xylose utilization	Xylulose kinase (EC 2.7.1.17)
290.	scaffold11|size4199117_2885097_2886263	Monosaccharides	Xylose utilization	Xylose-responsive transcription regulator, ROK family
	scaffold11|size4199117_2881671_2880250	Monosaccharides	Xylose utilization	D-xylose proton-symporter XylE
291.	scaffold12|size137365_16269_15217	Monosaccharides	D-Sorbitol(D-Glucitol) and L-Sorbose Utilization	PTS system, glucitol/sorbitol-specific IIB component and second of two IIC components (EC 2.7.1.69)
292.	scaffold12|size137365_15184_14825	Monosaccharides	D-Sorbitol(D-Glucitol) and L-Sorbose Utilization	PTS system, glucitol/sorbitol-specific IIA component (EC 2.7.1.69)
293.	scaffold12|size137365_16814_16287	Monosaccharides	D-Sorbitol(D-Glucitol) and L-Sorbose Utilization	PTS system, glucitol/sorbitol-specific IIC component (EC 2.7.1.69)
294.	scaffold11|size4199117_2385161_2385931; scaffold12|size137365_18634_17351	Monosaccharides	D-Sorbitol(D-Glucitol) and L-Sorbose Utilization	Sorbitol-6-phosphate 2-dehydrogenase (EC 1.1.1.140)
295.	scaffold12|size137365_14768_13830	Monosaccharides	D-Sorbitol(D-Glucitol) and L-Sorbose Utilization	Sorbitol operon transcription regulator
296.	scaffold11|size4199117_1310309_1311007	Monosaccharides	L-Arabinose utilization	L-ribulose-5-phosphate 4-epimerase (EC 5.1.3.4)
297.	scaffold11|size4199117_1308684_1310315	Monosaccharides	L-Arabinose utilization	Ribulokinase (EC 2.7.1.16)
298.	scaffold11|size4199117_1311038_1312462	Monosaccharides	L-Arabinose utilization	L-arabinose isomerase (EC 5.3.1.4)
299.	scaffold11|size4199117_1308344_1307238	Monosaccharides	L-Arabinose utilization	Transcriptional repressor of arabinoside utilization operon, GntR family
300.	Scaffold10|size375231_311160_312545; scaffold11|size4199117_1312608_1313987	Monosaccharides	L-Arabinose utilization	Arabinose-proton symporter

Table S14. Phosphorous metabolism genes in *B.aryabhattai* AB211

No	Contig_id/Size/start-end	Subcategory	Subsystem	Function (EC No.)
1	scaffold11|size4199117_4024708_4022867	Phosphorus Metabolism - no subcategory	Phosphoenolpyruvatephosphomutase	2-aminoethylphosphonate:pyruvate aminotransferase (EC 2.6.1.37)
2	scaffold1|size41991 17_4027341_4025824	Phosphorus Metabolism - no subcategory	Phosphoenolpyruvatephosphomutase	Phosphoenolpyruvatephosphomutase (EC 5.4.2.9)
3	scaffold11|size4199117_4025831_4024701	Phosphorus Metabolism - no subcategory	Phosphoenolpyruvatephosphomutase	Phosphonopyruvate decarboxylase (EC 4.1.1.82)
4	scaffold11|size4199117_286524_287411	Phosphorus Metabolism - no subcategory	High affinity phosphate transporter and control of PHO regulon	Phosphate transport system permease protein PstC (TC 3.A.1.7.1)
5	scaffold11|size4199117_383932_384741	Phosphorus Metabolism - no subcategory	High affinity phosphate transporter and control of PHO regulon	Phosphate transport ATP-binding protein PstB (TC 3.A.1.7.1)
6	scaffold11|size4199117_287408_288337	Phosphorus Metabolism - no subcategory	High affinity phosphate transporter and control of PHO regulon	Phosphate transport system permease protein PstA (TC 3.A.1.7.1)
7	scaffold11|size4199117_384764_385423	Phosphorus Metabolism - no subcategory	High affinity phosphate transporter and control of PHO regulon	Phosphate transport system regulatory protein PhoU
8	scaffold11|size4199117_138048_138785; scaffold11|size4199117_1837122_1837874	Phosphorus Metabolism - no subcategory	High affinity phosphate transporter and control of PHO regulon	Alkaline phosphatase synthesis transcriptional regulatory protein PhoP
9	scaffold11|size4199117_285515_286399	Phosphorus Metabolism - no subcategory	High affinity phosphate transporter and control of PHO regulon	Phosphate ABC transporter, periplasmic phosphate-binding protein PstS (TC 3.A.1.7.1)
10	scaffold11|size4199117_3474489_3472366	Phosphorus Metabolism - no subcategory	High affinity phosphate transporter and control of PHO regulon	Polyphosphate kinase (EC 2.7.4.1)
11	scaffold11|size4199117_138778_140550; scaffold11|size4199117_1403484_1404947	Phosphorus Metabolism - no subcategory	High affinity phosphate transporter and control of PHO regulon	Phosphate regulon sensor protein PhoR (SphS) (EC 2.7.13.3)
12	scaffold11|size4199117_3476089_3474563	Phosphorus Metabolism - no subcategory	Phosphate metabolism	Exopolyphosphatase (EC 3.6.1.11)

13	scaffold11|size4199117_349670_350644	Phosphorus Metabolism - no subcategory	Phosphate metabolism	Phosphate starvation-inducible protein PhoH, predicted ATPase
14	scaffold11|size4199117_2207686_2208612	Phosphorus Metabolism - no subcategory	Phosphate metabolism	Manganese-dependent inorganic pyrophosphatase (EC 3.6.1.1)
15	scaffold11|size4199117_3432243_3430915	Phosphorus Metabolism - no subcategory	Phosphate metabolism	Predicted ATPase related to phosphate starvation-inducible protein PhoH
16	Scaffold10|size375231_328586_330484; scaffold11|size4199117_3574812_3573157	Phosphorus Metabolism - no subcategory	Phosphate metabolism	Alkaline phosphatase (EC 3.1.3.1)
18	scaffold11|size4199117_3119956_3121266; scaffold11|size4199117_4192229_4191612	Phosphorus Metabolism - no subcategory	Phosphate metabolism	Alkaline phosphatase like protein
19	Scaffold10|size375231_141147_140500	Phosphorus Metabolism - no subcategory	Phosphate metabolism	PyrophosphatasePpaX (EC 3.6.1.1)

Table S15. Tryptophan/Auxin/Acetoin/2,3-butandiol biosynthesis genes in *B.aryabhattai* AB211

No	Contig_id/Size/start-end	Subcategory	Subsystem	Function (EC No.)
1	scaffold11|size4199117_558425_559939	Aromatic amino acids and derivatives	Tryptophan synthesis/auxin	Anthranilate synthase, aminase component (EC 4.1.3.27)
2	scaffold11|size4199117_559932_560957; scaffold11|size4199117_1770755_1771795	Aromatic amino acids and derivatives	Tryptophan synthesis/auxin	Anthranilatephosphoribosyltransferase (EC 2.4.2.18)
3	scaffold11|size4199117_561704_562330	Aromatic amino acids and derivatives	Tryptophan synthesis/auxin	Phosphoribosylanthranilateisomerase (EC 5.3.1.24)
4	scaffold11|size4199117_560947_561714	Aromatic amino acids and derivatives	Tryptophan synthesis/auxin	Indole-3-glycerol phosphate synthase (EC 4.1.1.48)
5	scaffold11|size4199117_563528_564343	Aromatic amino acids and derivatives	Tryptophan synthesis/auxin	Tryptophan synthase alpha chain (EC 4.2.1.20)
6	scaffold11|size4199117_563528_564343	Aromatic amino acids and derivatives	Tryptophan synthesis/auxin	Tryptophan synthase beta chain (EC 4.2.1.20)
7	scaffold11|size4199117_562327_563541	IAA production	Tryptophan synthesis/auxin	Nitrilase/cyanide hydratase and apolipoprotein N-acyltransferase (EC:3.5.5.1)
8	scaffold11|size4199117_3783159_3782401	Fermentation	Acetoin, butanediol metabolism	Acetolactate synthase, catabolic (EC 2.2.1.6)
9	scaffold11|size4199117_2860360_2859326	Fermentation	Acetoin, butanediol metabolism	Acetoin dehydrogenase E1 component beta-subunit (EC 1.2.4.-)
10	scaffold11|size4199117_2861371_2860379	Fermentation	Acetoin, butanediol metabolism	Acetoin dehydrogenase E1 component alpha-subunit (EC 1.2.4.-)
11	scaffold11|size4199117_1420874_1419828	Fermentation	Acetoin, butanediol metabolism	2,3-butanediol dehydrogenase, R-alcohol forming, (R)- and (S)-acetoin-specific (EC 1.1.1.4)
12	scaffold11|size4199117_2856414_2854537	Fermentation	Acetoin, butanediol metabolism	Transcriptional activator of acetoin dehydrogenase operon AcoR
13	scaffold11|size4199117_204286_206004; scaffold11|size4199117_3077198_3075603; scaffold11|size4199117_3855293_3853536	Fermentation	Acetoin, butanediol metabolism	Acetolactate synthase large subunit (EC 2.2.1.6)
14	scaffold11|size4199117_4021613_4022326	Fermentation	Acetoin, butanediol metabolism	Alpha-acetolactate decarboxylase (EC 4.1.1.5)
15	scaffold11|size4199117_2859300_2858074	Fermentation	Acetoin, butanediol metabolism	Dihydrolipoamide acetyltransferase component (E2) of acetoin dehydrogenase complex (EC 2.3.1.-)

16	scaffold11|size4199117_206001_206513	Fermentation	Acetoin, butanediol metabolism	Acetolactate synthase small subunit (EC 2.2.1.6)
17	scaffold11|size4199117_2858057_2856678	Central carbohydrate metabolism	Dehydrogenase complexes	Dihydrolipoamide dehydrogenase of acetoin dehydrogenase (EC 1.8.1.4)
